# DBU/H_2_O: a probing medium for diastereoselective Diels–Alder reactions of (arylvinyl)cyclohex-2-en-1-one dienes with a symmetrical *trans* dienophile

**DOI:** 10.1039/d6ra03633c

**Published:** 2026-08-12

**Authors:** M. Saeed Abaee, Zahra Shabani, Mohammad M. Mojtahedi

**Affiliations:** a Organic Chemistry Department, Chemistry and Chemical Engineering Research Center of Iran P.O. Box 14335-186 Tehran Iran abaee@ccerci.ac.ir

## Abstract

A DBU/H_2_O medium was used to explore the reaction of isophorone 1 with aromatic aldehydes at room temperature. The reaction gave adducts 3*via* selective substitution at the exocyclic methyl position γ to the C=O group of 1, as opposed to reacting with one of the two available active endocyclic methylenic sites. A slight temperature increase in the same mixture produced the respective diene 4. Stepwise treatment of 4 with dimethyl fumarate in refluxing toluene gave a mixture of the two possible *endo* Diels–Alder adducts 5 and 5′ in an approximately 5 : 1 ratio, through a highly stereospecific *endo* pathway, while addition of SnCl_4_ to the same mixture solely produced 5. Alternatively, one-pot combination of 1, the aldehydes, and the dienophile gave a 19 : 1 mixture of 5 and 5′ within 2 h treatment of the reactants in DBU/H_2_O mixture. NMR analysis and X-ray crystallography of the adducts confirmed the proposed structures for 5 and 5′.

## Abstract 2

A DBU/H_2_O medium was used to assess the stereoselectivity of the DA reactions of isophorone-derived dienes with DMF, giving the major *endo* adduct only (19 : 1). In contrast, the thermal reaction in PhMe produced a 5 : 1 mixture of two adducts.

## Graphical abstract

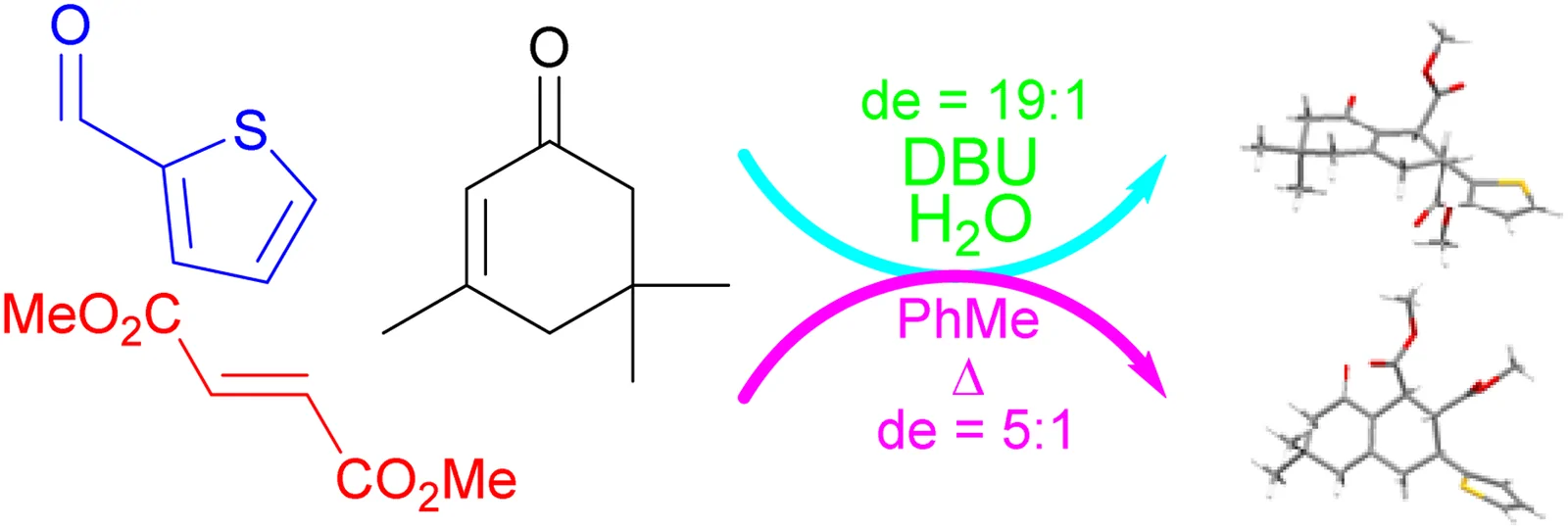

## Introduction

The combination of various reactions into a single one-pot process in a way that the product of each step behaves as the reactant for the next step, namely tandem, cascade, or domino reactions,^1,2^ constitutes a key strategy in modern organic chemistry by finding an increasing number of applications^3–5^ in the construction of complex synthetic^6,7^ and natural^8,9^ molecular targets. As a consequence, numerous articles,^10–12^ reviews,^13–15^ monographs,^16^ and books^17,18^ have been published in recent decades to highlight and summarize the overwhelming volume of results obtained from various investigations and applications of this strategy in today's chemistry.

**Fig. 1:**
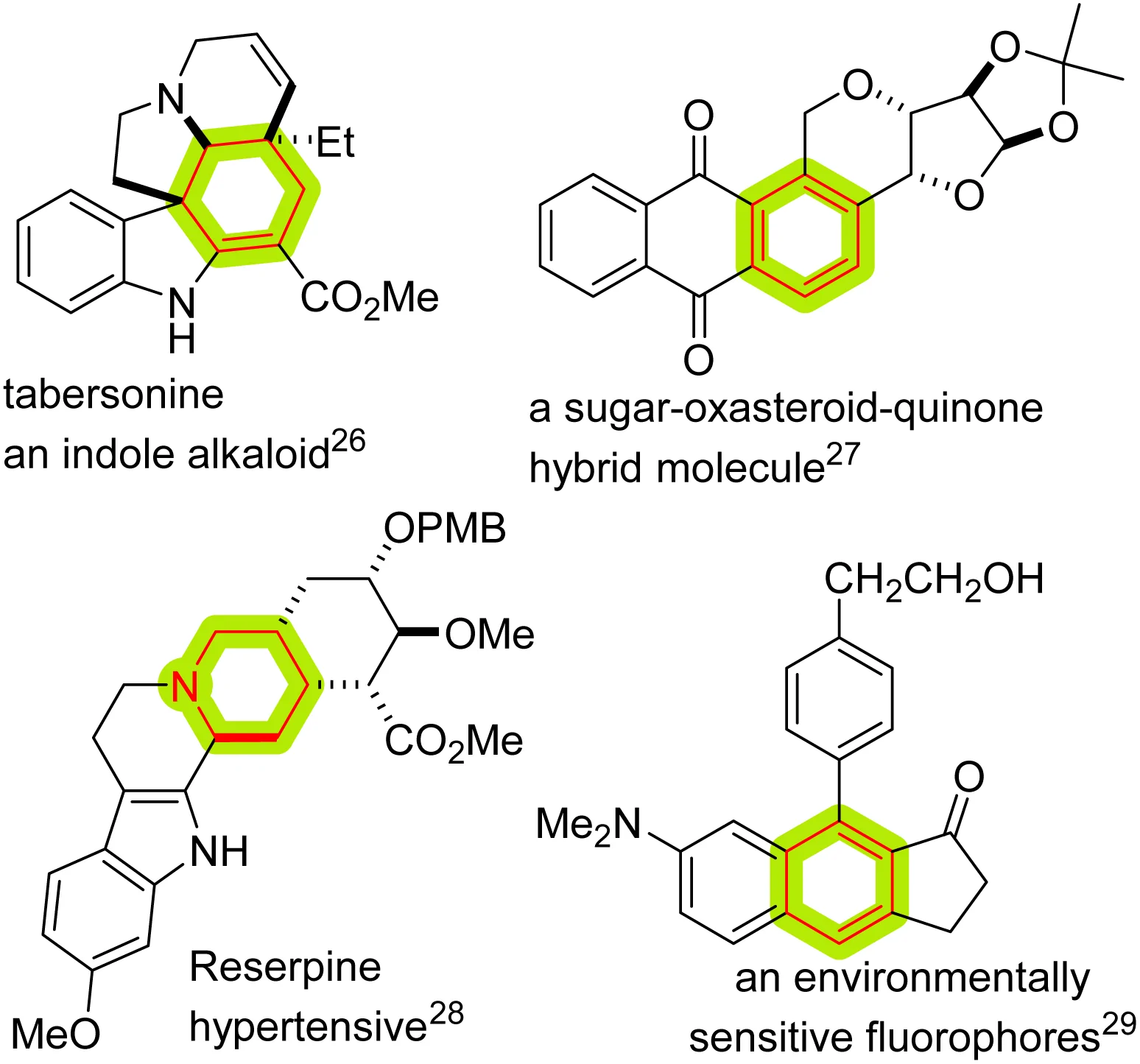
Some important structures synthesized *via* one-pot reactions. The central rings prepared *via* a key DA step are highlighted.

The Diels–Alder (DA) reaction is arguably the most important synthetic transformation in organic chemistry,^19^ evidenced by the non-stopping growth in the number of every day scientific reports^20,21^ being communicated. This feature has kept the DA process alive and progressive through the nearly past 100 years. The process involves the [4 + 2] cycloaddition of a conjugated diene to an activated alkene to form a cyclohexene ring^22^ having up to four stereogenic centers.^23^ Inclusion of the reaction as a determining step in tandem processes has resulted in even broader scopes of interesting synthetic manures.^24,25^ A few instances of important synthetic and natural structures that are prepared *via* one-pot and total synthesis procedures involving the DA reaction as the key step are presented in Fig. 1.^26–29^

**Fig. 2:**
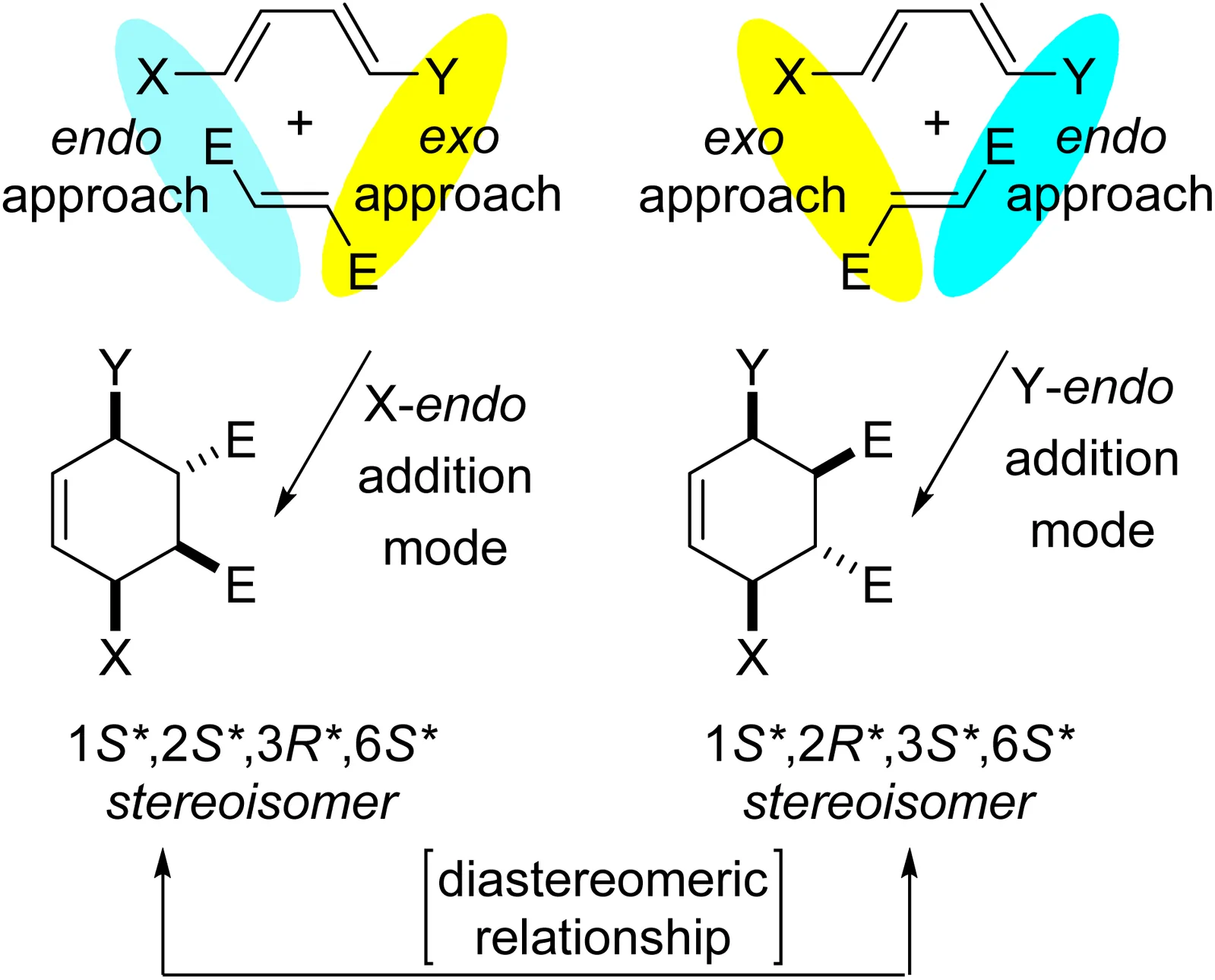
A typical DA reaction of an unsymmetrically substituted diene with a symmetrical *trans* dienophile (*e.g.*, DMF).

An outstanding feature of the DA reaction is the predictability of the stereoselectivity of the process by maintaining the relative steric configuration of dienes and dienophiles in the structures of their respective products.^30^ In this context, when an unsymmetrically substituted diene reacts with a *trans* dienophile, two diastereomeric *endo* products (SSRS *vs.* SRSS) would be expected to form,^31^ even if the dienophile substituents would be the same (as exemplified in Fig. 2 for the cycloaddition of dimethyl fumarate (DMF)^32^ with an unsymmetrically substituted 1,4-disubstituted butadiene). Thus, selective formation of one of the two possible *endo* isomers, now would be an issue of synthetic challenge.

**Scheme 1:**
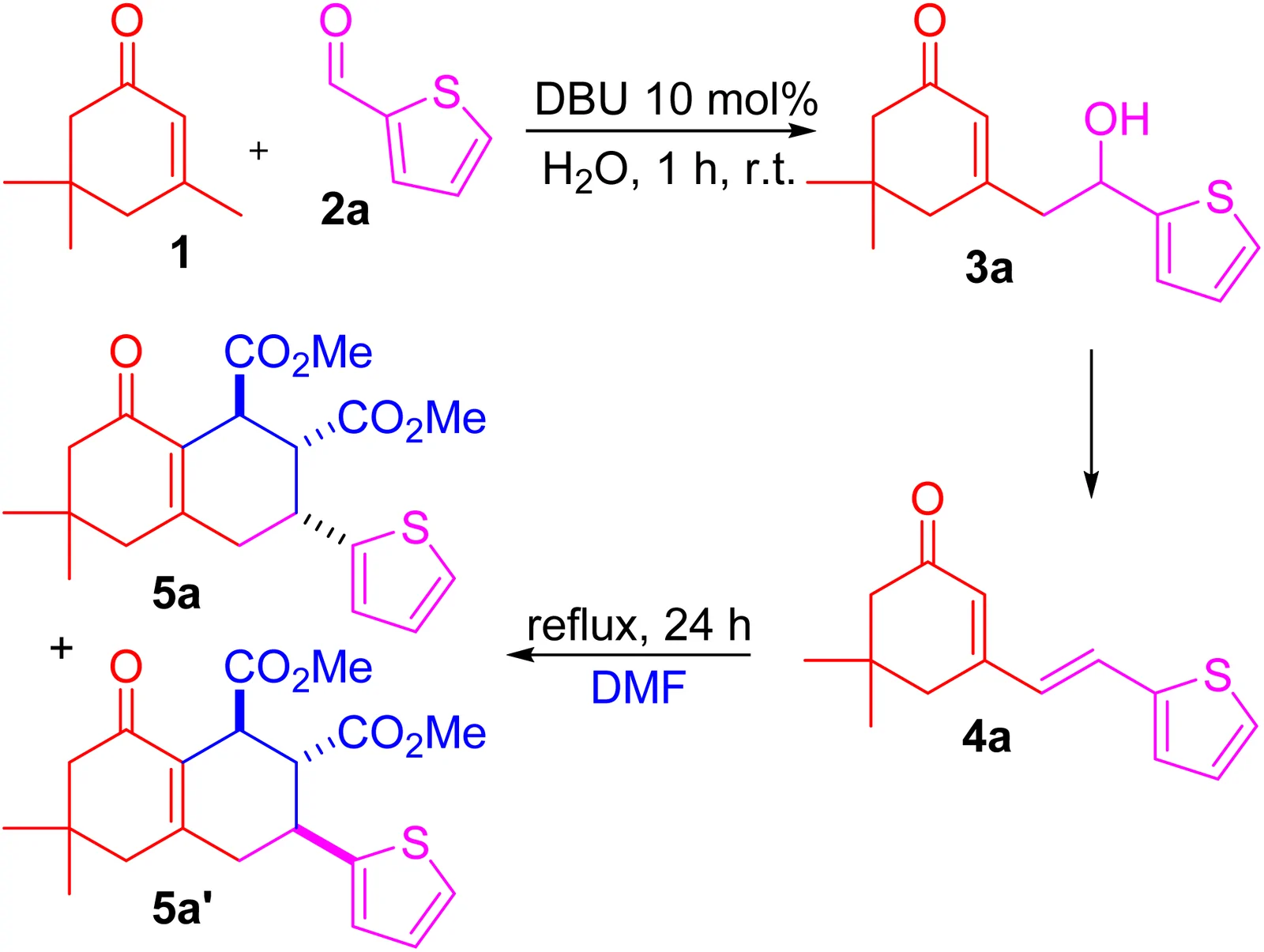
General reaction pathways.

We have been investigating the synthesis and DA reactivity of different diene systems,^33^ including the studies on isophorone-based dienones.^34–36^ In continuation, we are persuaded now to look into the stereoselectivity of the reaction of dienones 4 with DMF (Scheme 1), where the reaction basically has the potential to produce two DA adducts, in any of which the CO_2_Me group of the dienophile that ends up with *endo* position would become *exo* in the other product. As a result of this work, either the stepwise (1 + 2 → 4 and then 4 + DMF → 5) or the one-pot process (1 + 2 + DMF → 5) in aqueous DBU would produce different derivatives of the final products with preferential formation of the adducts with the CO_2_Me group *endo* to the aromatic ring.

**Fig. 3:**
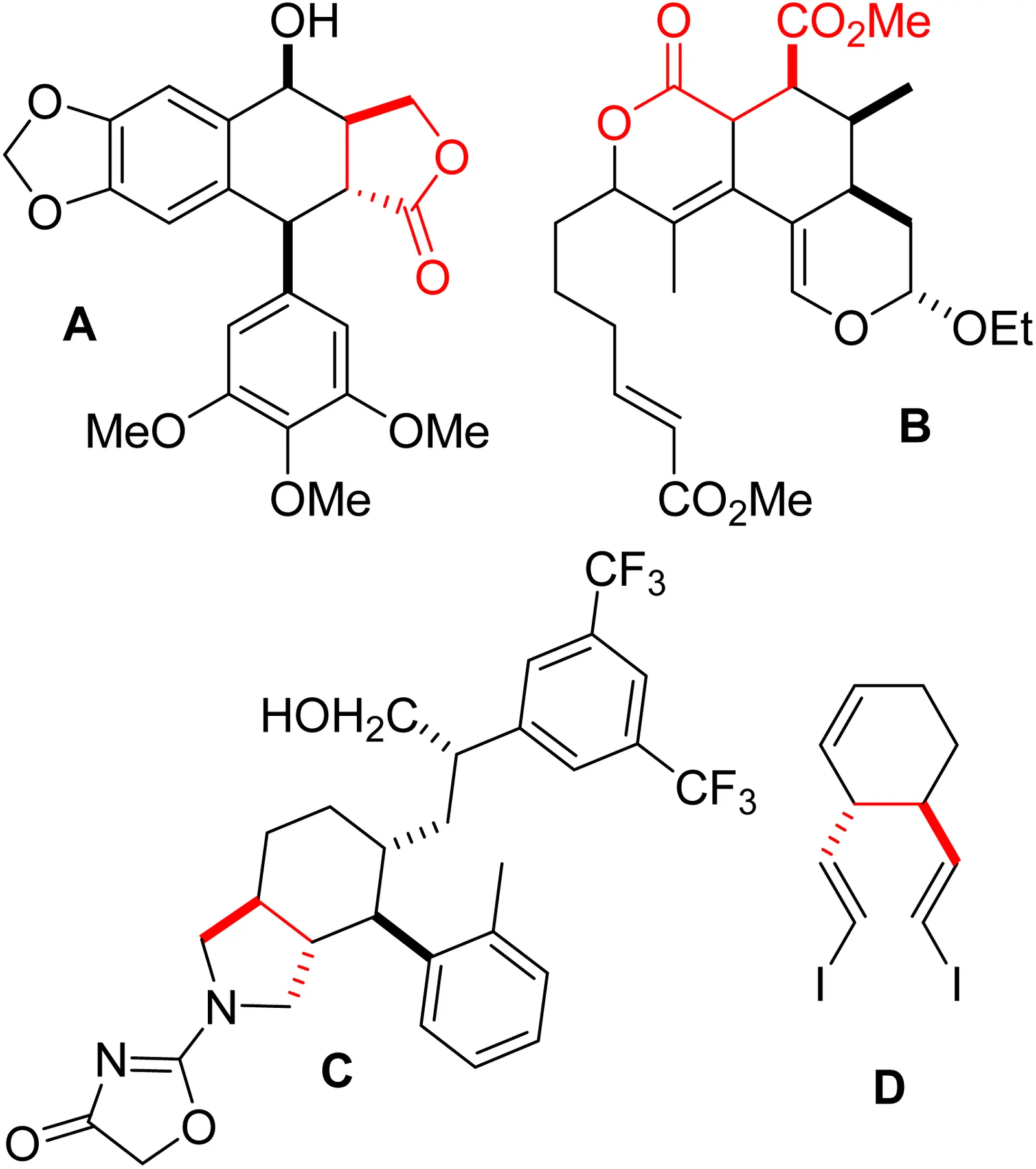
Some important natural or synthetic molecular targets containing DMF fragment in their structure.

It is noteworthy to mention that DMF is an important building block in organic synthesis and serves as a key dienophile in DA cycloadditions. To highlight the applications of DMF, a few of natural or synthetic molecules which include DMF in their structures are depicted in Fig. 3 (A: epiisopodophyllotoxin, a naturally occurring lignan lactone;^37^B: a precursor to quassinoid skeletons;^38^C: a synthetic receptor antagonist and a potent human NK1 receptor antagonist;^39^D: polycephalin C, a natural product responsiblefor yellow colour of plasmodia of *physarum polycepahlum*^40^).

## Results and discussion

We primarily optimized the first step reaction between isophorone and the aldehyde (Table 1). Thus, reaction of 1 with 2a in the presence of aqueous DBU gave aldol adduct 3a in high yield after 1 h treatment of the mixture at rt (entry 1). The same reaction in the absence of DBU (entry 2) or water (entry 3) either was hindered almost completely or had slow progress. This was more or less the case when water was replaced with other solvents (entries 4–5), different quantities of the catalyst was used (entries 6–7), or other organocatalysts were added instead of DBU (entries 8–11). Treatment of the same H_2_O/DBU (10%) mixture at 60 °C directly gave the aldol condensation product 4a after 2 h (entry 12).

**Table 1:** Optimization of the synthesis of 3a and 4a

Entry	Reactants	Conditions^a^	Temperature	Time (h)	Product	Yield (%)^b^
1	1a + 2a	DBU (10 mol%), H_2_O	rt	1	3a	84
2	1a + 2a	H_2_O	rt	6	3a	>5
3	1a + 2a	DBU (10 mol%)	rt	1	3a	21
4	1a + 2a	DBU (10 mol%), MeOH	rt	1	3a	>10
5	1a + 2a	DBU (10 mol%), EtOH	rt	1	3a	>10
6	1a + 2a	DBU (25 mol%), H_2_O	rt	1	3a	70
7	1a + 2a	DBU (50 mol%), H_2_O	rt	1	3a	60
8	1a + 2a	Et_2_NH (10 mol%), H_2_O	rt	1	3a	42
9	1a + 2a	Et_3_N (10 mol%), H_2_O	rt	1	3a	45
10	1a + 2a	DABCO (10 mol%), H_2_O	rt	1	3a	33
11	1a + 2a	Morpholine (10 mol%), H_2_O	rt	1	3a	29
12	1a + 2a	DBU (10 mol%), H_2_O	60 °C	2	4a	85

a 10 mol% DBU was used in all reactions.

b Isolated yields.

Next, we evaluated the reaction of the diene with DMF (Table 2). Thus, a 1.0 : 1.0 mixture of 4a and DMF gave a mixture of both of the two possible *endo* structures 5a and 5a′ after 18 h in refluxing toluene (entry 1). The ratio of the adducts was determined by the integration of the respective peaks in the ^1^H NMR spectrum of the reaction mixture to be 5 : 1 and verified by their quantitative isolation by column chromatography. In contrast, no product was obtained for the uncatalyzed (entry 2) or SnCl_4_-mediated reactions (entry 3) at room-temperature. Alternatively, 5a was exclusively formed by refluxing the 4a/DMF mixture in a SnCl_4_/toluene solution (entry 4). Use of other catalysts did not improve the outcome of the process (entries 5–7). Finally, the one-pot edition of the process was attempted, where initial 2 h treatment of 1 and 2a in DBU/H_2_O mixture followed by addition of the dienophile and 24 h reflux gave the DA adduct 5a exclusively (entry 8).

**Table 2:** Optimization of stepwise and one-pot synthesis of 5a and 5a′

Entry	Reactants	Conditions	5a : 5a′	Yield (%)^c^
1	4a + DMF	Toluene, reflux	5 : 1^a^	82
2	4a + DMF	Toluene, rt	—	0
3	4a + DMF	Toluene, SnCl_4_, rt	—	0
4	4a + DMF	Toluene, SnCl_4_, reflux	19 : 1^b^	90
5	4a + DMF	Toluene, TiCl_4_, reflux	—	40
6	4a + DMF	Toluene, AlCl_3_, reflux	—	35
7	4a + DMF	Toluene, ZrCl_4_, reflux	—	82
8	1 + 2a + DMF	(1) DBU/H_2_O, rt.	19** **: 1^b^	84
		(2) DBU/H_2_O, reflux		

a Determined by chromatography separation.

b Determined by ^1^H NMR integration of the reaction mixture.

c Isolated yields.

**Fig. 4:**
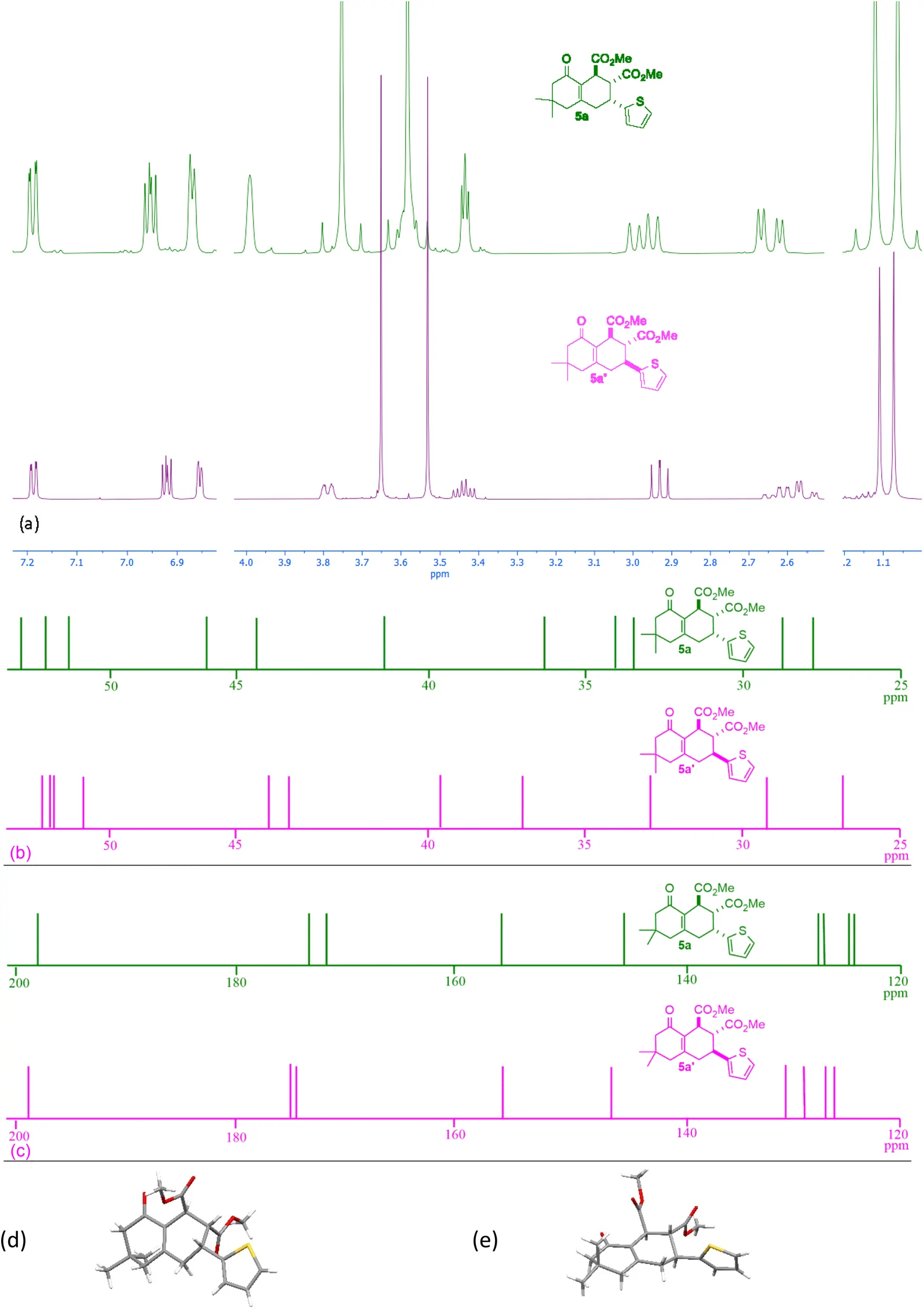
Comparative mapping of (a) ^1^H, (b) ^13^C high field, and (c) ^13^C low field NMR signals of 5a and 5a′. Ball and stick representation of crystal structures of (d) 5a (CCDC 2475957) and (e) 5a′ (CCDC 2475958), for more clarity, disorder of thiophene rings in both structures have been omitted.

Products 5a and 5a′ were identified based on the analysis of their NMR spectra. In the case of 5a, a singlet signal at 4.00 (H1) and three dd signals at 3.45 (H2), 3.00 (H4), and 2.65 ppm (H4′) were indicatives of the formation of the cyclohexene ring as the result of the DA cycloaddition. For 5a′, the signal at 4.00 moved to about 3.90 ppm, appearing as a dt due to coupling with the neighbouring H1 and the remote CH_2_ groups, respectively (Fig. 2a). In the ^13^C NMR spectrum of each of the adducts, twenty distinguishing signals appeared with different chemical shifts for each of the corresponding peaks (Fig. 4b and c). Furthermore, the structures of the two *endo* DA adducts were confirmed by X-ray crystallography analyses, as depicted in Fig. 4d–e. Relevant crystallographic data are presented in Table 4.^41^

To demonstrate the synthetic scope of the process, we then examined the one-pot combination of 1 with various derivatives of 2 and DMF under the one-pot optimized conditions (Table 3). Thus, when monitoring of 1.0 : 1.0 mixtures of isophorone and aromatic aldehydes in DBU/H_2_O showed nearly complete formation of their corresponding diene, addition of DMF to the same vessels and reflux of the mixtures produced the final *endo* adducts 5b–i in high yields. ^1^H NMR analysis of crude reaction mixtures prior to separation of the isomers showed that the diastereoselectivity excess (de) were approximately 19 : 1 (Table 4).

**Table 3:** One-pot synthesis of various derivatives of 5

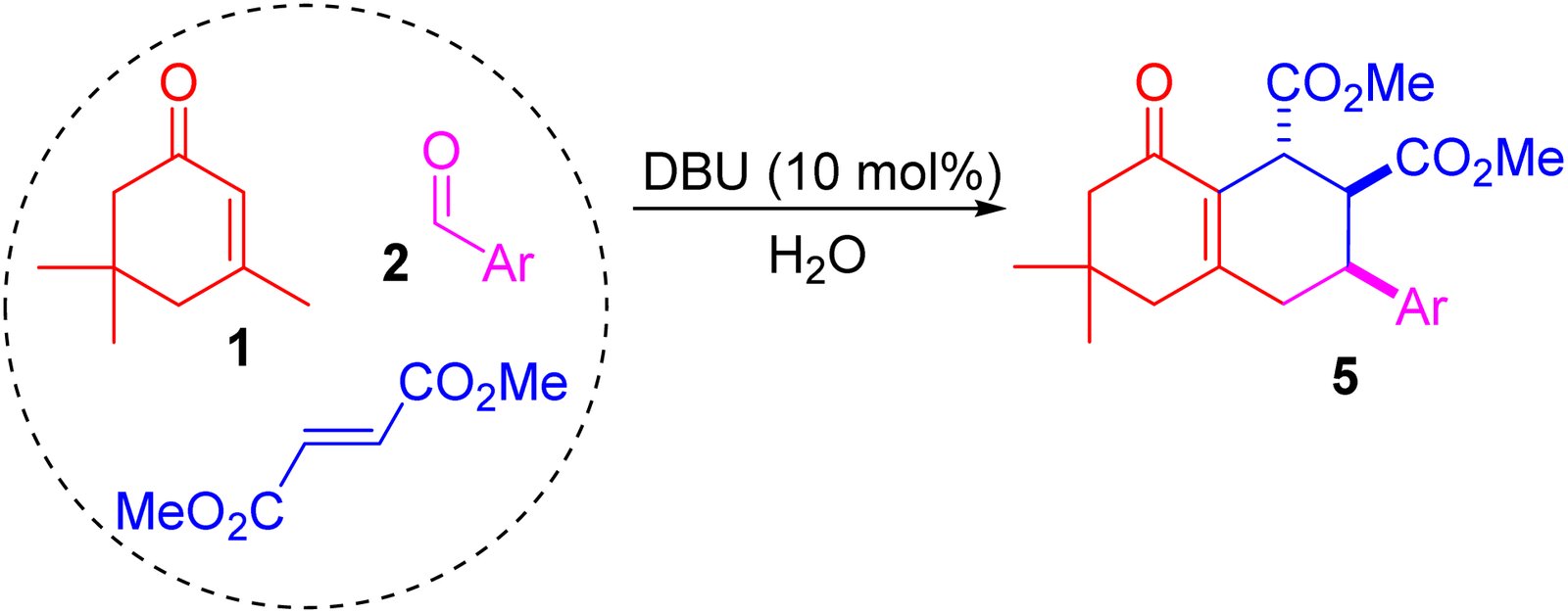			
Entry	Aldehyde (2)	Product	Yield (%)^a^^,^^b^
1	C_6_H_5_CHO	5b	80
2	4-MeC_6_H_4_CHO	5c	77
3	2,4-Me_2_C_6_H_3_CHO	5d	86
4	4-MeOC_6_H_4_CHO	5e	87
5	2,3,4-(MeO)_3_C_6_H_2_CHO	5f	78
6	2-ClC_6_H_4_CHO	5g	90
7	3-MeOC_6_H_4_CHO	5h	86
8	4-O_2_NC_6_H_4_CHO	5i	89

a Isolated yields.

b de for all reactions was 19 : 1.

**Table 4:** Crystallographic and structure refinement data for 5a and 5a′

	5a	5a′
Empirical formula	C_20_H_24_O_5_S	C_20_H_24_O_5_S
Formula weight/g mol^−1^	376.45	376.45
Crystal color, habit	Colorless, plate	Colorless, plate
Temperature/K	298(2)	298(2)
Wavelength *λ*/Å	0.71073	0.71073
Crystal system	Triclinic	Monoclinic
Space group	*P*1̄	*P*2_1_/*c*
Crystal size/mm	0.50 × 0.40 × 0.150	0.50 × 0.30 × 0.20
*a*/Å	8.7275(17)	15.337(3)
*b*/Å	10.798(2)	10.372(2)
*c*/Å	11.241(2)	12.200(2)
*α*/°	107.19(3)	90
*β*/°	93.42(3)	96.25(3)
*γ*/°	108.18(3)	90
Volume/Å^3^	948.0(4)	1929.0(7)
*Z*	2	4
*d*_calcd_/g cm^−3^	1.319	1.296
2*θ* range for data collection/deg	3.848 to 49.996	4.75 to 49.99
*F*(000)	400.0	800.0
Absorption coefficient/mm^−1^	0.198	0.195
Index ranges	−10 ≤ h ≤ 9	−17 ≤ h ≤ 18
	−12 ≤ k ≤ 12	−12 ≤ k ≤ 12
	−13 ≤ l ≤ 13	−14 ≤ l ≤ 14
Data collected	7185	11 748
Unique data (*R*_int_)	3325 (0.1557)	3389 (0.2097)
Parameter, restraints	252, 12	253, 12
*R*_1_ a, w*R*_2_^b^ [*I* > 2*σ*(*I*)]	0.0800, 0.1833	0.0739, 0.1589
GOF on *F*^2^ (S)	0.987	0.989
Largest diff peak, hole/e Å^−3^	0.28, −0.29	0.26, −0.23
CCDC no.	2475957	2475958

a *R*_1_ = Σ||*F*_0_| − |*F*_0_||/Σ|*F*_*0*_|.

b w*R*_2_ = [Σ(w(*F*_0_^2^ − *F*_c_^2^)^2^)/Σw(*F*_0_^2^)^2^]^1/2^.

**Fig. 5:**
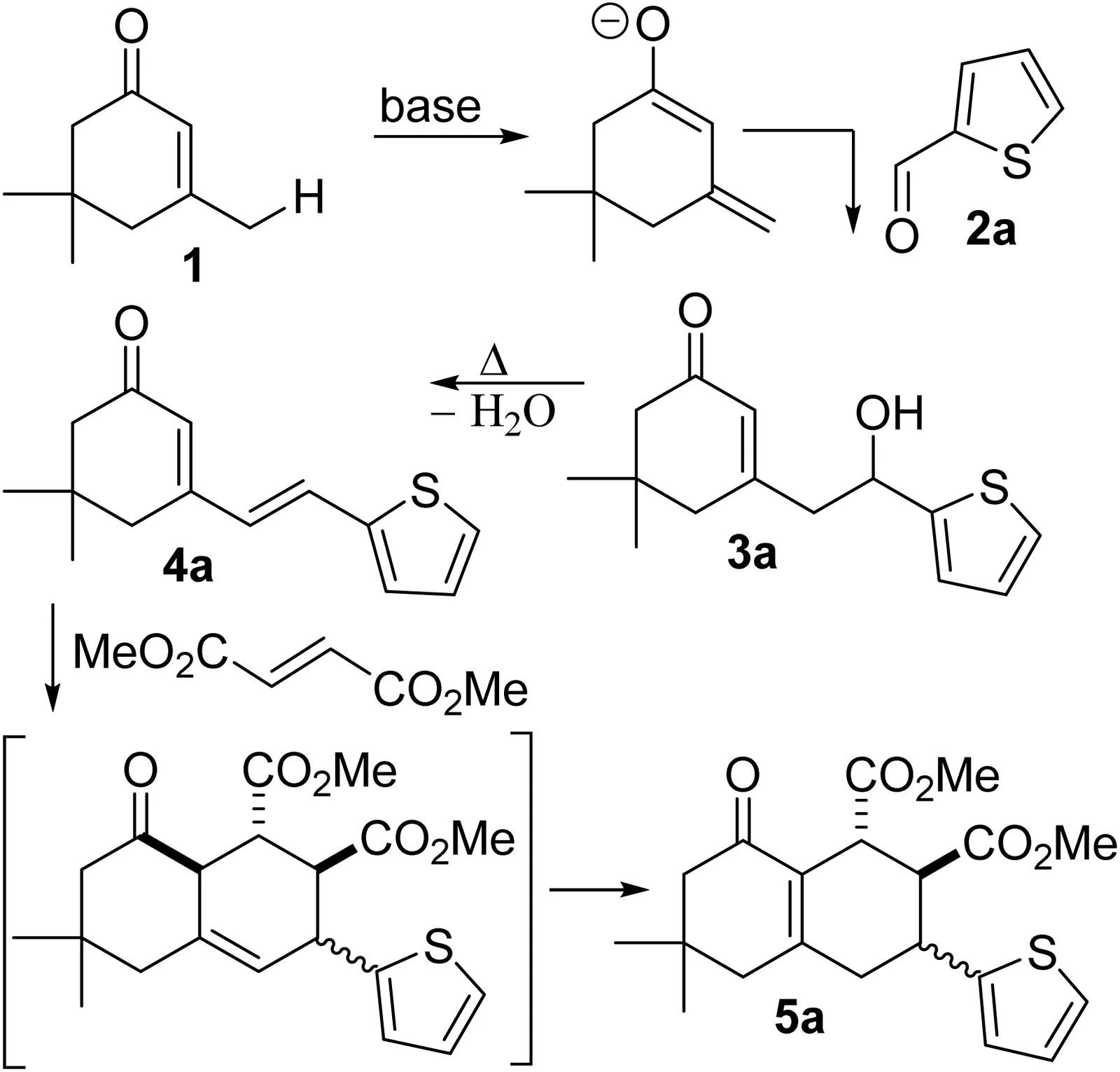
The proposed mechanism for the reaction steps.

Based on the results, a mechanism would be proposed for the process, as shown in Fig. 5 for the reaction of 1 with thiophene-2-carbaldehyde and DMF. In the presence of aqueous DBU, the γ acidic proton (Me group) is preferably removed to give the dienolate intermediate. This intermediate then attacks the carbonyl of 2a to complete the aldol reaction step and give 3a, which in turn produces 4a upon *in situ* heating or the use of a stronger basic medium. Cycloaddition of 4a to DMF followed by *in situ* rearrangement of the double bond to the more stable endocyclic position gives the final product 5a.

## Experimental

### General

Tin tetrachloride (SnCl_4_), is a corrosive, fuming liquid reagent that requires careful handling and safety precautions due to its hazardous nature. Safety precautions include wearing appropriate protective equipment such as gloves and eye protection. It is also recommended to carry out SnCl_4_-involved reactions under the fume hood. Melting points are uncorrected. FT-IR spectra were recorded using KBr disks on a Bruker Vector-22. NMR spectra were obtained on a FT-NMR Bruker Ultra Shield™ as CDCl_3_ solutions using TMS as internal standard reference. Elemental analyses were performed using a Thermo Finnigan Flash EA 1112 instrument. MS spectra were obtained on a Finnigan Mat 8430 apparatus at an ionization potential of 70 eV. TLC experiments were carried out on pre-coated silica gel plates using petroleum hexanes/EtOAc (5 : 1) as the eluent. All reagents and starting materials were purchased from the Merck Company.

### Stepwise thermal procedure for the synthesis of 5a and 5c

A mixture of 1.0 mmol of 4 (4a: Ar = 2-thienyl; 4c: 4-MeC_6_H_4_) and DMF (173 mg, 1.2 mmol) was refluxed in PhMe (5.0 mL) for 24 h (monitored by TLC using EtOAc/hexanes (1 : 5) as the eluent). The mixture was cooled to rt and concentrated under reduced pressure. The residue was fractionated by column chromatography (using silica gel and EtOAc/hexanes (1 : 6) as the eluent) to obtain the respective DA adducts 5a (256 mg, 68%) and 5a′ (51 mg, 13%) or 5c (296 mg, 77%) and 5c′ (58 mg, 15%).

### SnCl_4_-mediated procedure for the synthesis of 5a

A mixture of 4a (232 mg, 1.0 mmol), DMF (173 mg, 1.2 mmol), and SnCl_4_ (0.25 mmol) was refluxed in PhMe (5.0 mL) for 18 h (monitored by TLC using EtOAc/hexanes (1 : 5) as the eluent). The mixture was cooled to rt, diluted with EtOAC, and washed with saturated NaHCO_3_ solution. The organic layer was dried over Na_2_SO_4_ and concentrated under reduced pressure. The residue was fractionated by column chromatography (using silica gel and EtOAc/hexanes (1 : 6) as the eluent) to obtain the respective DA adduct 5a (338 mg, 90%).

### Sample spectral data of new products

#### Dimethyl (1R*,2R*,3S*)-6,6-dimethyl-8-oxo-3-(thiophen-2-yl)-1,2,3,4,5,6,7,8-octahydronaphthalene-1,2-dicarboxylate (5a)

White crystal, mp 140–141 °C; ^1^H NMR (500 MHz, CDCl_3_) *δ* 7.18 (dd, *J* = 1.5, 4.5 Hz, 1H), 6.95 (dd, *J* = 3.0, 4.5 Hz, 1H), 6.86 (dd, *J* = 1.5, 3.0 Hz, 1H), 4.00 (s, 1H), 3.75 (s, 3H), 3.60 (s, 3H), 3.44 (dd, *J* = 4.0, 6.0 Hz, 1H), 2.97 (dd, *J* = 6.0, 14.5 Hz, 1H), 2.65 (dd, *J* = 4.0, 14.5 Hz, 1H), 2.38 (d, 14.5 Hz, 1H), 2.36 (d, 14.5 Hz, 1H), 2.33–2.30 (m, 3H), 1.12 (s, 3H), 1.07 (s, 3H) ppm; ^13^C NMR (125 MHz, CDCl_3_) *δ* 197.8, 173.2, 171.8, 155.9, 144.7, 127.2, 126.7, 124.4, 123.9, 52.6, 51.9, 51.1, 46.8, 45.2, 41.3, 36.1, 33.9, 33.4, 28.7, 27.7 ppm; IR (KBr) 2954, 1737, 1667 cm^−1^; MS (70 eV) *m*/*z* 376 [M^+^], 344, 284, 257; anal. calcd for C_20_H_24_O_5_S: C, 63.81; H, 6.43; S, 8.52. Found: C, 63.70; H, 6.67; S, 8.81.

#### Dimethyl (1S*,2S*,3S*)-6,6-dimethyl-8-oxo-3-(thiophen-2-yl)-1,2,3,4,5,6,7,8-octahydronaphthalene-1,2-dicarboxylate (5a′)

White crystal, mp 140–142 °C; ^1^H NMR (500 MHz, CDCl_3_) *δ* 7.17 (dd, *J* = 1.5, 4.5 Hz, 1H), 6.92 (dd, *J* = 3.0, 4.5 Hz, 1H), 6.86 (dd, *J* = 1.5, 3.0 Hz, 1H), 3.79 (dt, *J* = 2.0, 10.0 Hz, 1H), 3.65 (s, 3H), 3.53 (s, 3H), 3.44 (ddd, *J* = 6.0, 11.0, 11.0 Hz, 1H), 2.93 (dd, *J* = 11.0, 11.0 Hz, 1H), 2.63 (ddd, *J* = 5.0, 11.0, 15.5 Hz, 1H), 2.57 (dd, 6.5, 15.0 Hz, 1H), 2.36 (d, 15.0 Hz, 2H), 2.30 (d, 15.0 Hz, 2H), 1.11 (s, 3H), 1.07 (s, 3H) ppm; ^13^C NMR (125 MHz, CDCl_3_) *δ* 197.1, 173.3, 172.8, 154.2, 144.2, 128.4, 126.6, 124.8, 124.0, 52.0, 51.8, 51.7, 50.7, 44.9, 44.3, 39.5, 36.9, 32.9, 29.2, 26.8 ppm; IR (KBr) 2988. 1762, 1243 cm^−1^; MS (70 eV) *m*/*z* 376 [M^+^], 344, 284, 257; anal. calcd for C_20_H_24_O_5_S: C, 63.81; H, 6.43; S, 8.52. Found: C, 63.70; H, 6.67; S, 8.81.

#### Dimethyl (1R*,2S*,3S*)-6,6-dimethyl-8-oxo-3-phenyl-1,2,3,4,5,6,7,8-octahydronaphthalene-1,2-dicarboxylate (5b)

White crystal, mp 105–107 °C; ^1^H NMR (500 MHz, CDCl_3_) *δ* 7.31 (t, *J* = 7.5 Hz, 2H), 7.26 (t, *J* = 1.5, 7.5 Hz, 1H), 7.19 (d, *J* = 7.5 Hz, 2H), 3.97 (s, 1H), 3.75 (s, 3H), 3.50 (s, 3H), 3.38 (dd, *J* = 2.5, 3.5 Hz, 1H), 3.24 (ddd, *J* = 4.5, 5.0, 11.0 Hz, 1H), 3.03 (dd, *J* = 11.0, 18.0 Hz, 1H), 2.45 (dd, *J* = 11.0, 18.0 Hz, 1H), 2.40 (d, 18.0 Hz, 1H), 2.38 (d, 18.0 Hz, 1H), 2.35–2.29 (m, 2H), 1.12 (s, 3H), 1.06 (s, 3H) ppm; ^13^C NMR (125 MHz, CDCl_3_) *δ* 197.8, 173.0, 171.9, 156.4, 141.0, 130.0, 128.3, 127.2, 126.9, 52.4, 51.3, 51.0, 46.5, 45.2, 41.6, 37.7, 33.7, 33.3, 28.7, 27.3 ppm; IR (KBr) 2954. 1734, 1666 cm^−1^; MS (70 eV) *m*/*z* 370 [M^+^], 338, 278, 251; anal. calcd for C_22_H_26_O_5_: C, 71.33; H, 7.07. Found: C, 71.46; H, 7.20.

#### Dimethyl (1R*,2S*,3S*)-6,6-dimethyl-8-oxo-3-(p-tolyl)-1,2,3,4,5,6,7,8-octahydronaphthalene-1,2-dicarboxylate (5c)

White crystal, mp 137–139 °C; ^1^H NMR (500 MHz, CDCl_3_) *δ* 7.12 (d, *J* = 8.0 Hz, 2H), 7.07 (d, *J* = 8.0 Hz, 2H), 3.98 (s, 1H), 3.75 (s, 3H), 3.50 (s, 3H), 3.35 (dd, *J* = 3.0, 3.0 Hz, 1H), 3.19 (ddd, *J* = 5.5, 5.0, 11.0 Hz, 1H), 3.00 (ddd, *J* = 1.0, 11.0, 18.0 Hz, 1H), 2.43 (dd, *J* = 6.0, 18.5 Hz, 1H), 2.39 (d, 16.0 Hz, 1H), 2.36 (d, 16.0 Hz, 1H), 2.34 (d, *J* = 15.0 Hz, 1H), 2.32 (s, 3H), 2.31 (d, *J* = 15.0 Hz, 1H), 1.11 (s, 3H), 1.05 (s, 3H) ppm; ^13^C NMR (125 MHz, CDCl_3_) *δ* 197.8, 173.1, 172.0, 156.5, 137.0, 136.5, 129.1, 127.1, 126.9, 52.4, 51.4, 51.0, 46.5, 45.2, 41.6, 37.3, 33.9, 33.3, 28.7, 27.3, 20.9 ppm; IR (KBr) 2953, 1732, 1664 cm^−1^; MS (70 eV) *m*/*z* 384 [M^+^], 352, 292, 265; anal. calcd for C_23_H_28_O_5_: C, 71.85; H, 7.34. Found: C, 71.93; H, 7.51.

#### Dimethyl (1S*,2R*,3S*)-6,6-dimethyl-8-oxo-3-(p-tolyl)-1,2,3,4,5,6,7,8-octahydronaphthalene-1,2-dicarboxylate (5c′)

White crystal, mp 137–140 °C; ^1^H NMR (500 MHz, CDCl_3_) *δ* 7.11 (d, *J* = 8.0 Hz, 2H), 7.08 (d, *J* = 8.0 Hz, 2H), 3.76 (d, *J* = 10.0 Hz, 1H), 3.65 (s, 3H), 3.43 (s, 3H), 3.03 (ddd, *J* = 5.0, 11.0, 14.0 Hz, 1H), 2.97 (d, *J* = 11.0 Hz, 1H), 2.94 (d, *J* = 11.0 Hz, 1H), 2.54 (ddd, *J* = 3.0, 11.0, 18.0 Hz, 1H), 2.42 (d, 16.0 Hz, 1H), 2.37 (d, 16.0 Hz, 1H), 2.31 (s, 3H), 2.30 (d, *J* = 15.0 Hz, 1H), 2.29 (d, *J* = 15.0 Hz, 1H), 1.10 (s, 3H), 1.07 (s, 3H) ppm ^13^C NMR (125 MHz, CDCl_3_) *δ* 197.4, 173.9, 173.1, 155.2, 138.1, 136.9, 129.3, 128.6, 127.3, 52.1, 51.7, 50.9, 50.5, 45.2, 44.7, 41.5, 39.2, 32.0, 29.4, 26.9, 21.0 ppm; IR (KBr) 3445, 2928, 1738, 1667 cm^−1^; MS (70 eV) *m*/*z* 384 [M^+^], 352, 292, 265, 207; anal. calcd for C_23_H_28_O_5_: C, 71.85; H, 7.34. Found: C, 71.93; H, 7.51.

#### Dimethyl (1R*,2S*,3S*)-3-(2,4-dimethylphenyl)-6,6-dimethyl-8-oxo-1,2,3,4,5,6,7,8-octahydronaphthalene-1,2-dicarboxylate (5d)

White crystal, mp 139–141 °C; ^1^H NMR (500 MHz, CDCl_3_) *δ* 7.06 (d, *J* = 7.5 Hz, 1H), 6.99 (d, *J* = 7.5 Hz, 1H), 6.96 (s, 1H), 3.96 (s, 1H), 3.74 (s, 3H), 3.48 (s, 3H), 3.40 (ddd, *J* = 4.0, 7.5, 14.0 Hz, 1H), 3.22 (d, *J* = 3.5 Hz, 1H), 3.10 (dd, *J* = 12.0, 18.0 Hz, 1H), 2.40 (d, *J* = 15.5 Hz, 1H), 2.37 (d, 15.5 Hz, 1H), 2.36–2.33 (m, 2H), 2.29 (s, 3H), 2.28–2.26 (m, 1H), 2.20 (s, 3H), 1.12 (s, 3H), 1.07 (s, 3H) ppm; ^13^C NMR (125 MHz, CDCl_3_) *δ* 198.1, 173.1, 172.1, 157.3, 136.4, 135.7, 131.7, 126.9, 126.6, 126.1, 52.4, 51.4, 51.1, 45.4, 44.9, 42.0, 33.6, 33.5, 33.3, 29.1, 27.3, 20.9, 18.9 ppm; IR (KBr) 2952, 1736, 1667 cm^−1^; MS (70 eV) *m*/*z* 398 [M^+^], 366, 306, 279; anal. calcd for C_24_H_30_O_5_: C, 72.34; H, 7.59. Found: C, 72.49; H, 7.68.

#### Dimethyl (1R*,2S*,3S*)-3-(4-methoxyphenyl)-6,6-dimethyl-8-oxo-1,2,3,4,5,6,7,8-octahydronaphthalene-1,2-dicarboxylate (5e)

White crystal, mp 153–155 °C; ^1^H NMR (500 MHz, CDCl_3_) *δ* 7.10 (d, *J* = 8.5 Hz, 2H), 6.84 (d, *J* = 8.5 Hz, 2H), 3.95 (s, 1H), 3.79 (s, 3H), 3.74 (s, 3H), 3.50 (s, 3H), 3.33 (dd, *J* = 2.5, 3.5 Hz, 1H), 3.19 (ddd, *J* = 4.5, 5.0, 11.0 Hz, 1H), 2.99 (dd, *J* = 11.0, 18.0 Hz, 1H), 2.46–2.38 (m, 2H), 2.36–2.30 (m, 3H), 1.11 (s, 3H), 1.06 (s, 3H) ppm; ^13^C NMR (125 MHz, CDCl_3_) *δ* 197.7, 173.1, 172.0, 158.4, 156.5, 133.1, 128.2, 126.9, 113.7, 55.1, 52.3, 51.3, 51.0, 46.6, 45.2, 41.6, 37.0, 34.1, 33.3, 28.7, 27.4 ppm; IR (KBr) 3418, 2924, 1731, 1664 cm^−1^; MS (70 eV) *m*/*z* 400 [M^+^], 368, 308, 281; anal. calcd for C_23_H_28_O_6_: C, 68.98; H, 7.05. Found: C, 69.12; H, 6.85.

#### Dimethyl (1R*,2S*,3S*)-6,6-dimethyl-8-oxo-3-(2,3,4-trimethoxyphenyl)-1,2,3,4,5,6,7,8-octahydronaphthalene-1,2-dicarboxylate (5f)

White crystal, mp 155–157 °C; ^1^H NMR (500 MHz, CDCl_3_) *δ* 6.86 (d, *J* = 8.0 Hz, 1H), 6.61 (d, *J* = 8.0 Hz, 1H), 3.97 (s, 1H), 3.88 (s, 3H), 3.86 (s, 3H), 3.84 (s, 3H), 3.77 (s, 3H), 3.53–3.50 (m, 1H), 3.47 (s, 3H), 3.42 (dd, *J* = 2.5, 3.5 Hz, 1H), 2.99 (dd, *J* = 14.0, 18.0 Hz, 1H), 2.40 (d, 14.5 Hz, 1H), 2.36 (d, 14.5 Hz, 1H), 2.32–2.25 (m, 3H), 1.12 (s, 3H), 1.06 (s, 3H) ppm; ^13^C NMR (125 MHz, CDCl_3_) *δ* 198.0, 173.1, 172.3, 156.9, 152.8, 151.7, 142.1, 127.2, 126.8, 121.4, 107.3, 61.1, 60.7, 55.9, 52.4, 51.7, 51.4, 51.1, 45.4, 42.1, 33.4, 33.1, 30.8, 29.1, 27.4 ppm; IR (KBr) 2952, 1734, 1663 cm^−1^; MS (70 eV) *m*/*z* 460 [M^+^], 368, 341, 207; anal. calcd for C_25_H_32_O_8_: C, 65.20; H, 7.00. Found: C, 65.33; H, 7.15.

#### Dimethyl (1R*,2S*,3S*)-3-(2-chlorophenyl)-6,6-dimethyl-8-oxo-1,2,3,4,5,6,7,8-octahydronaphthalene-1,2-dicarboxylate (5g)

White crystal, mp 138–141 °C; ^1^H NMR (500 MHz, CDCl_3_) *δ* 7.38 (dd, *J* = 1.5, 7.5 Hz, 1H), 7.30 (dd, *J* = 1.5, 7.5 Hz, 1H), 7.24 (ddd, *J* = 1.5, 7.5, 7.5 Hz, 1H), 7.20 (ddd, *J* = 1.5, 7.5, 7.5 Hz, 1H), 4.01 (s, 1H), 3.75 (s, 3H), 3.71–3.67 (m, 1H), 3.49 (d, *J* = 3.0 Hz, 1H), 3.46 (s, 3H), 3.12 (dd, *J* = 12.0, 18.5 Hz, 1H), 2.41 (d, 14.5 Hz, 1H), 2.37 (d, 14.5 Hz, 1H), 2.33–2.28 (m, 3H), 1.13 (s, 3H), 1.05 (s, 3H) ppm; ^13^C NMR (125 MHz, CDCl_3_) *δ* 197.9, 172.7, 171.9, 156.1, 138.0, 134.2, 129.8, 128.3, 128.1, 127.2, 126.7, 52.4, 51.4, 51.1, 45.4, 44.1, 41.9, 34.2, 33.4, 32.6, 28.9, 27.4 ppm; IR (KBr) 4440, 2925, 1734, 1661 cm^−1^; MS (70 eV) *m*/*z* 404 [M^+^], 372, 312, 285; anal. calcd for C_22_H_25_ClO_5_: C, 65.26; H, 6.22. Found: C, 65.40; H, 6.37.

#### Dimethyl (1R*,2S*,3S*)-3-(2-methoxyphenyl)-6,6-dimethyl-8-oxo-1,2,3,4,5,6,7,8-octahydronaphthalene-1,2-dicarboxylate (5h)

White crystal, mp 103–105 °C; ^1^H NMR (500 MHz, CDCl_3_) *δ* 7.23 (dd, *J* = 8.0, 8.0 Hz, 1H), 6.80–6.76 (m, 2H), 6.73 (dd, *J* = 1.5, 8.0 Hz, 1H), 3.96 (s, 1H), 3.79 (s, 3H), 3.75 (s, 3H), 3.51 (s, 3H), 3.38 (dd, *J* = 2.5, 3.5 Hz, 1H), 3.23–3.18 (m, 1H), 3.01 (dd, *J* = 12.0, 18.0 Hz, 1H), 2.45 (dd, 5.5, 18.0 Hz, 1H), 2.40 (d, 15.5 Hz, 1H), 2.366–2.29 (m, 3H), 1.11 (s, 3H), 1.06 (s, 3H) ppm; ^13^C NMR (125 MHz, CDCl_3_) *δ* 197.7, 173.0, 171.9, 159.6, 156.4, 142.8, 129.3, 126.9, 119.5, 113.4, 111.9, 55.1, 52.4, 51.4, 51.0, 46.3, 45.2, 41.6, 37.7, 33.9, 33.3, 28.7, 27.3 ppm; IR (KBr) 3431, 2926, 1726, 1600 cm^−1^; MS (70 eV) *m*/*z* 400 [M^+^], 368, 308, 281; anal. calcd for C_23_H_28_O_6_: C, 68.98; H, 7.05. Found: C, 68.11; H, 7.13.

#### Dimethyl (1R*,2S*,3S*)-6,6-dimethyl-3-(4-nitrophenyl)-8-oxo-1,2,3,4,5,6,7,8-octahydronaphthalene-1,2-dicarboxylate (5i)

White crystal, mp 147–149 °C; ^1^H NMR (500 MHz, CDCl_3_) *δ* 8.11 (d, *J* = 8.5 Hz, 2H), 7.33 (d, *J* = 8.5 Hz, 2H), 4.02 (s, 1H), 3.76 (s, 1H), 3.69 (s, 3H), 3.43 (s, 3H), 3.37 (dd, *J* = 2.5, 3.5 Hz, 1H), 3.29–3.24 (m, 1H), 3.02 (dd, *J* = 12.0, 18.0 Hz, 1H), 2.41 (dd, *J* = 7.0, 18.0 Hz, 1H), 2.41 (d, *J* = 18.0 Hz, 1H), 2.31–2.23 (m, 2H), 1.05 (s, 3H), 0.98 (s, 3H) ppm; ^13^C NMR (125 MHz, CDCl_3_) *δ* 197.7, 173.1, 172.0, 158.4, 156.5, 133.1, 128.2, 126.9, 113.7, 55.1, 52.3, 51.3, 51.0, 46.6, 45.2, 41.6, 37.0, 34.1, 33.3, 28.7, 27.4 ppm; ^13^C NMR (125 MHz, CDCl_3_) *δ* 197.6, 172.9, 171.4, 155.3, 148.9, 147.0, 128.4, 127.2, 123.7, 52.7, 51.8, 51.1, 46.2, 45.3, 41.6, 37.6, 33.5, 33.2, 28.8, 27.4 ppm; IR (KBr) 344-, 2924, 1733, 1660 cm^−1^; MS (70 eV) *m*/*z* 415 [M^+^], 383, 323, 296; anal. calcd for C_22_H_25_NO_7_: C, 63.61; H, 6.07. Found: C, 63.58; H, 6.23.

## Conclusions

In summary, the aqueous DBU medium is basic enough to cause the aldol reaction between 1 and 2 to give the intermediate 3, which easily converts to the corresponding diene 4 in a short time period by mild heating. Consequently, the DA product would be obtained efficiently either through direct heating with DMF in toluene or using catalytic amounts of SnCl_4_ or DBU. The cycloaddition is stereoselective and produces the respective *endo* adducts in high yields. In addition, the results suggest that the *endo* directing effect of an aromatic group is higher than a carbonyl moiety, when both unsaturated groups exist on the terminal positions of the diene.

To rationalize the observed stereoselectivity in the reactions of the dienes with DMF, we reviewed nearly two hundreds of literature reports regarding [4 + 2] cycloadditions of DMF with various unsymmetrically substituted dienes. The results summarized in Table 5 show that for the dienes with one of the two terminal groups being hydrogen (G = H), the preferential or the dominant product would be the *endo*-G in the majority of cases (entries 1–6). However, when *G* is an aryl group, regardless of what *G*′ is, the preferred product would be the corresponding *exo*-G stereoisomer (entries 7–11). In contrast to these observations, in the current work we have obtained the products in which the CO_2_Me group is *endo* to the aryl substituents. These observations would be further explored in practice and theory in our laboratory in the near future.

**Table 5:** Stereoselectivity overview of the reactions of unsymmetrically substituted 1,2-dienes with DMF

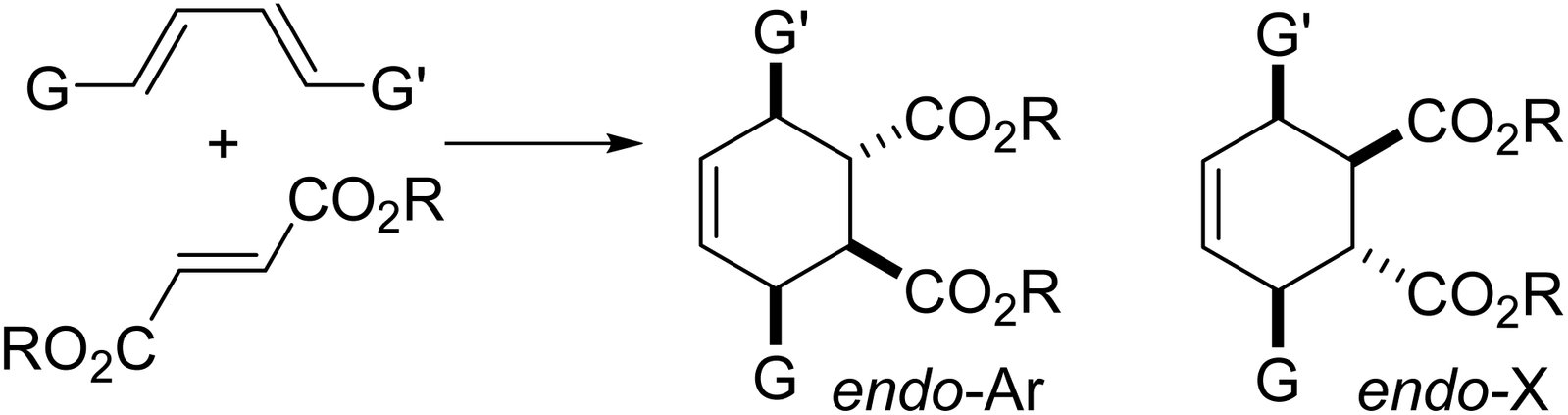				
Entry	*G*	*G*′	Major product	References
1	H	CONR_2_	*endo*-G	42
2	H	OH	*endo*-G	43
3	H	OR	*endo*-G	44
4	H	NMe_2_	*endo*-G	45
5	H	SO_2_R	*endo*-G	46
6	H	CH_2_SePh	*endo*-G	47
7	Ar	H	*exo*-G	48
8	Ar	H	*exo*-G	39
9	Ar	OMe	*exo*-G	49
10	Ar	OTBDMS	*exo*-G	50
11	Ar	COR	*exo*-G	This work

## Author contributions

Conceptualization, methodology, supervision and writing the original draft, M. S. A.; investigation and experiments, Z. S.; validation, M. M. M. All authors have given approval to the final version of the manuscript.

## Conflicts of interest

There are no conflicts to declare.

## Supplementary Material

RA-016-D6RA03633C-s001

RA-016-D6RA03633C-s002

## Data Availability

CCDC 2475957 and 2475958 contain the supplementary crystallographic data for this paper.^51*a*,*b*^ The data supporting this article have been included as part of the supplementary information (SI). Supplementary information: ^1^H NMR, ^13^C NMR, IR, and mass spectra for all new products, and the crystal data as well. See DOI: https://doi.org/10.1039/d6ra03633c.
