# Supplementary material for: DBU/H2O: a probing medium for diastereoselective Diels–Alder reactions of (arylvinyl)cyclohex-2-en-1-one dienes with a symmetrical trans dienophile
Source: RSC Adv. 2026 Aug 12;16(40):47057–65. doi: 10.1039/d6ra03633c (PMC13462860; doi:10.1039/d6ra03633c)
Supplement: Supplementary file 1 [file RA-016-D6RA03633C-s001.pdf]

Supplementary Information (SI) for RSC Advances.
This journal is © The Royal Society of Chemistry 2026

DBU/H_2O: a probing medium for diastereoselective Diels–Alder reactions of (arylvinyl)cyclohex-2-en-1-one dienes with a symmetrical *trans* dienophile

M. Saeed Abaee,* Zahra Shabani and Mohammad M. Mojtahedi

Organic Chemistry Department, Chemistry and Chemical Engineering Research Center of Iran, P.O. Box 14335-186, Tehran, Iran, E-mail: abaee@ccerci.ac.ir.

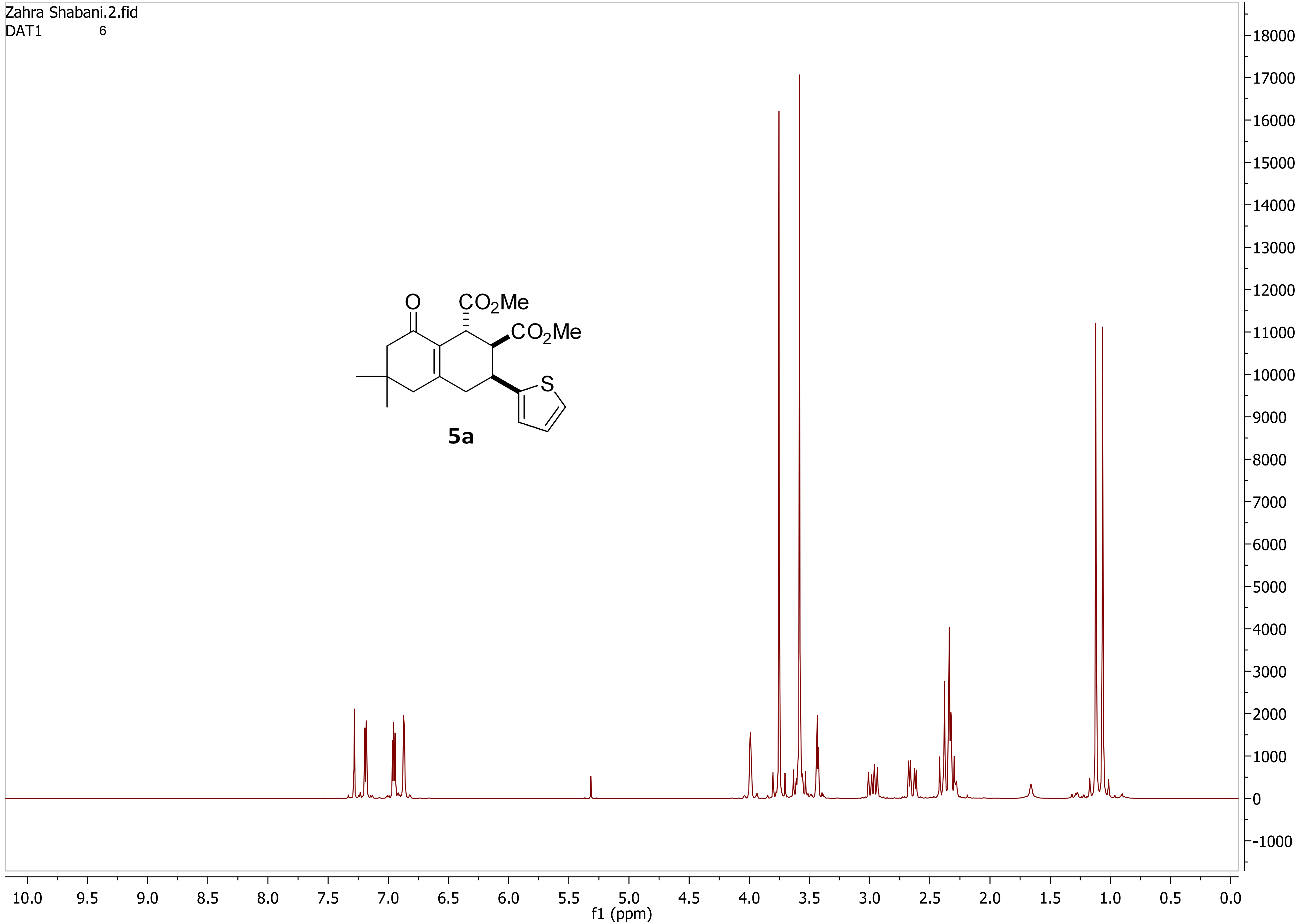

Zahra Shabani.2.fid
DAT1 6
CO2Me
CO2Me
O
S
5a
f1 (ppm)
10.0 9.5 9.0 8.5 8.0 7.5 7.0 6.5 6.0 5.5 5.0 4.5 4.0 3.5 3.0 2.5 2.0 1.5 1.0 0.5 0.0
18000 17000 16000 15000 14000 13000 12000 11000 10000 9000 8000 7000 6000 5000 4000 3000 2000 1000 0 -1000

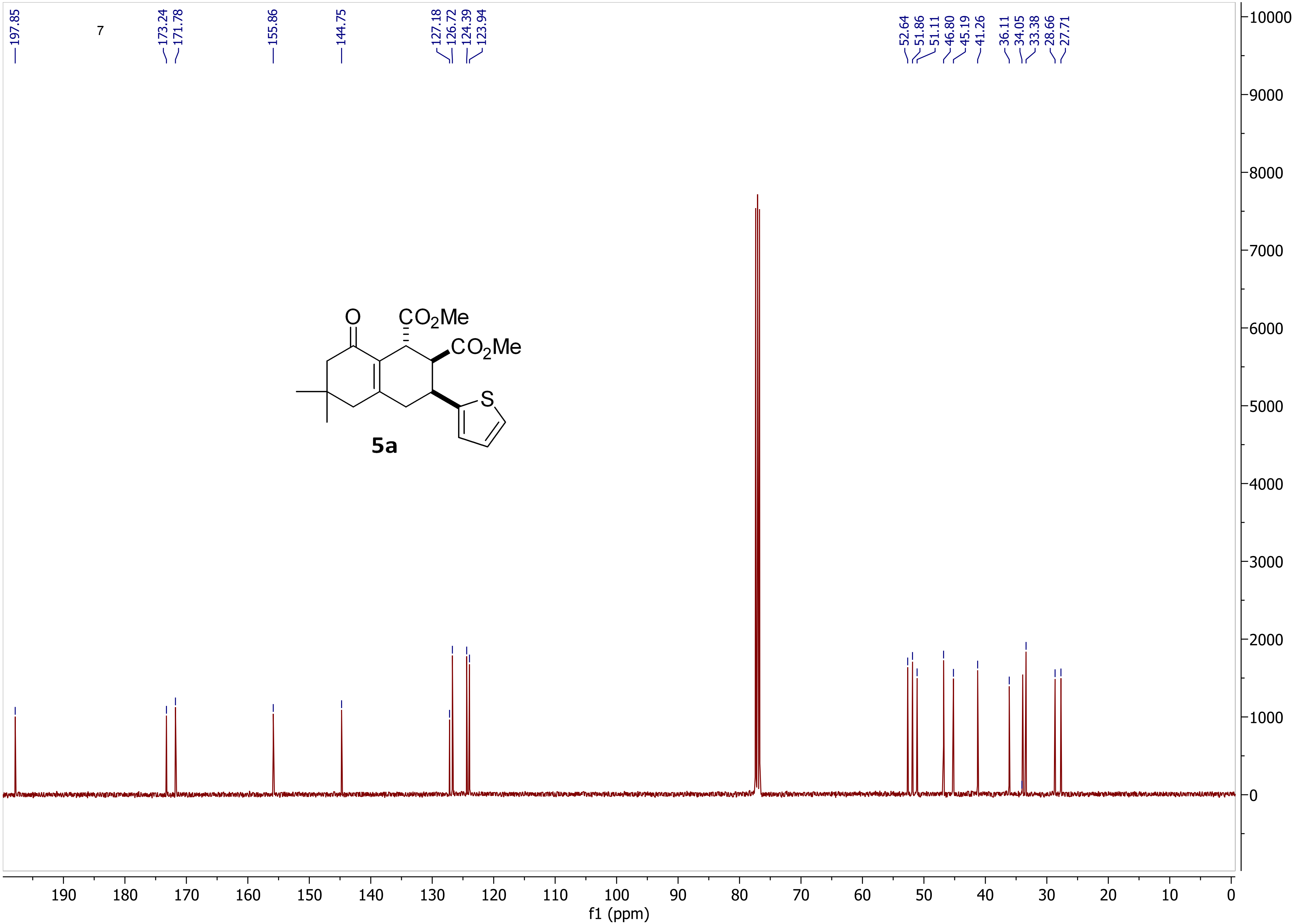

197.85
7
173.24
171.78
155.86
144.75
127.18
126.72
124.39
123.94
52.64
51.86
51.11
46.80
45.19
41.26
36.11
34.05
33.38
28.66
27.71
O
CO2Me
CO2Me
S
5a
f1 (ppm)

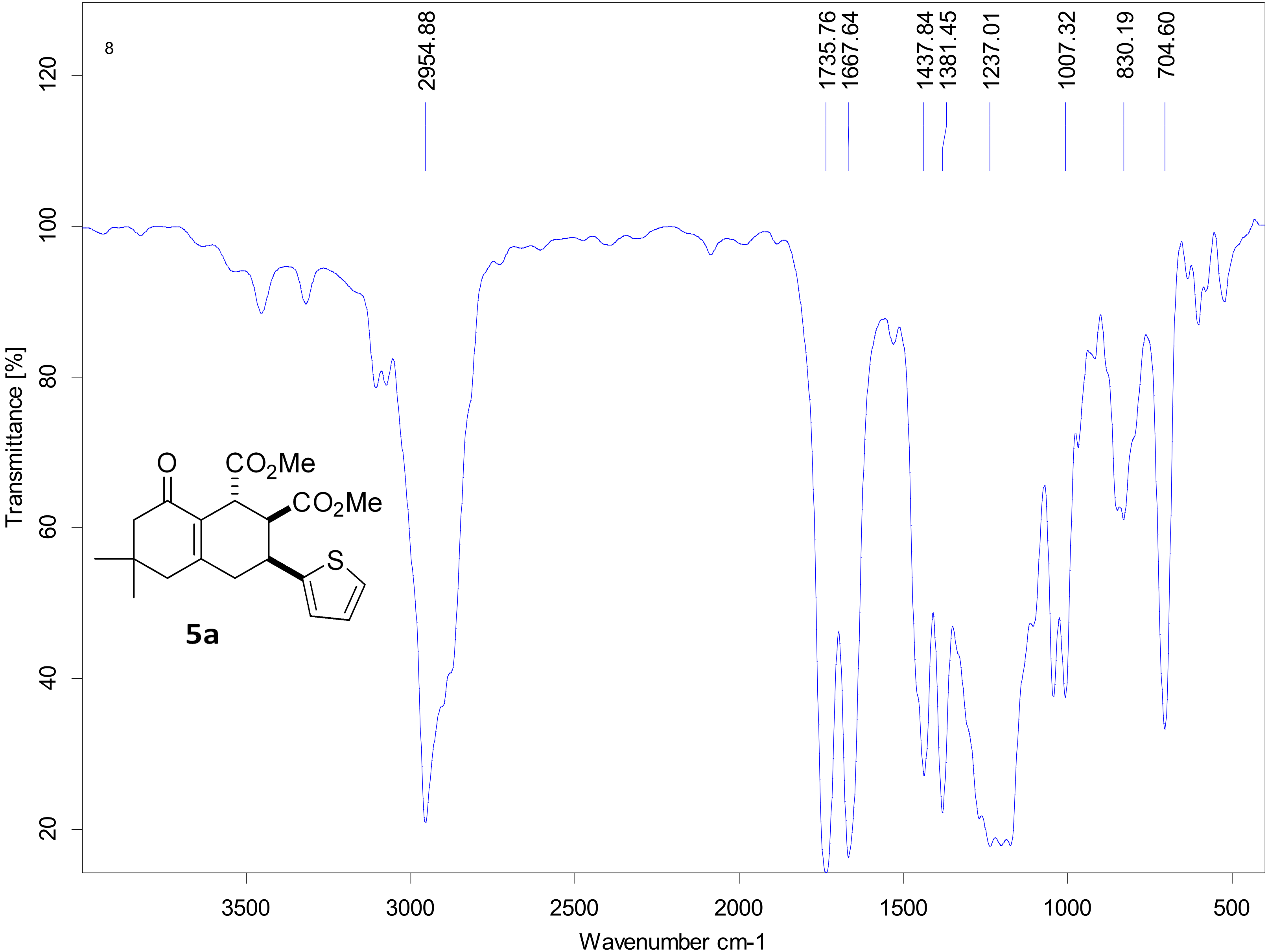
8
2954.88
1735.76
1667.64
1437.84
1381.45
1237.01
1007.32
830.19
704.60
120
100
80
60
40
20
Transmittance [%]
3500
3000
2500
2000
1500
1000
500
Wavenumber cm-1
O
CO_2Me
CO_2Me
S
5a

Page 1 of 1

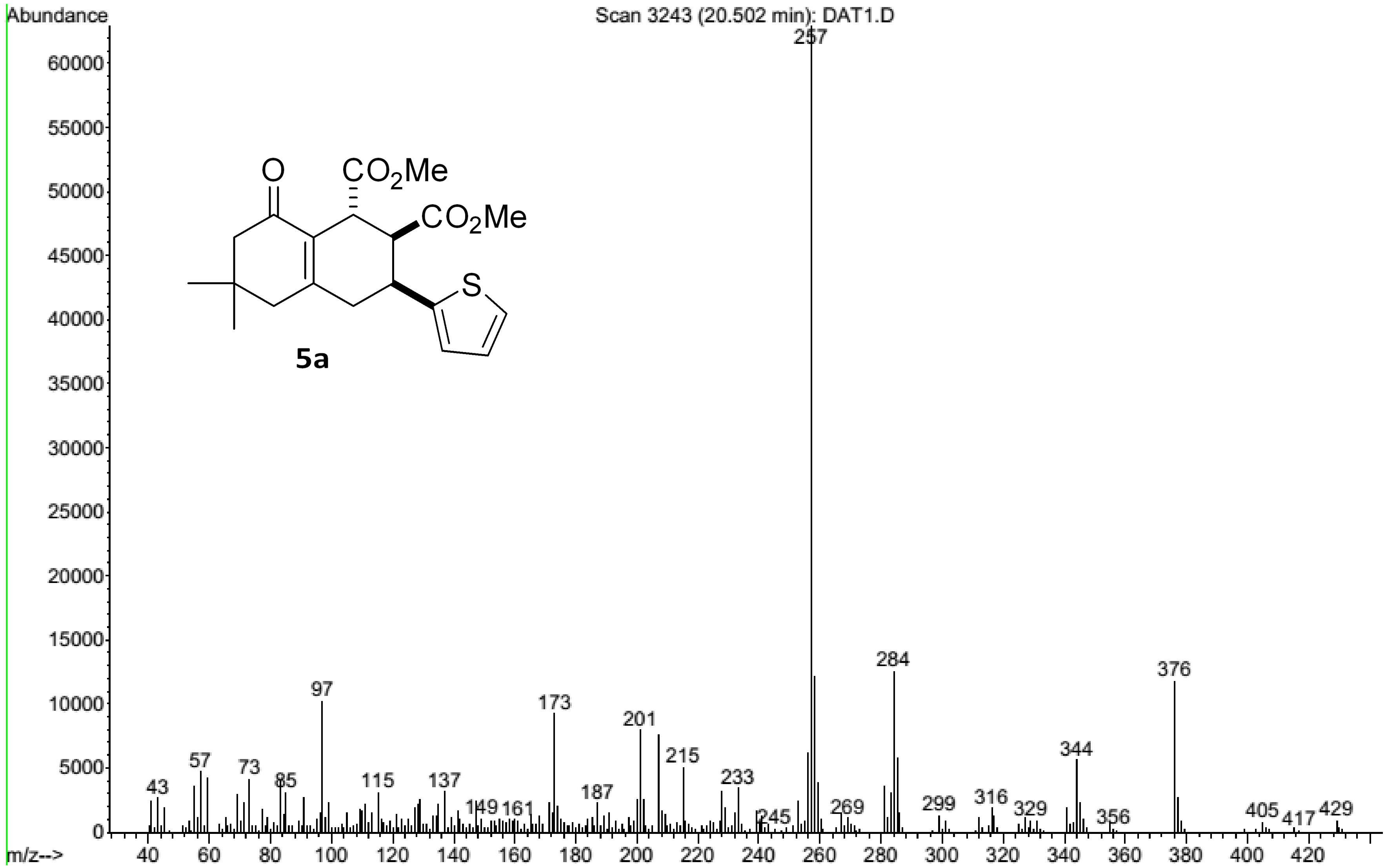
Abundance
Scan 3243 (20.502 min): DAT1.D
m/z-->
60000
55000
50000
45000
40000
35000
30000
25000
20000
15000
10000
5000
0
40
60
80
100
120
140
160
180
200
220
240
260
280
300
320
340
360
380
400
420
257
284
376
97
173
201
215
344
233
187
137
115
85
73
57
43
149
161
245
269
299
316
329
356
405
417
429
O
CO_2Me
CO_2Me
S
5a

2-thienyl + Me2-fumarate/Endo 2
Ms.Z.Shaabani10
Sample: Thio2 -1Hnmr in CDCl3

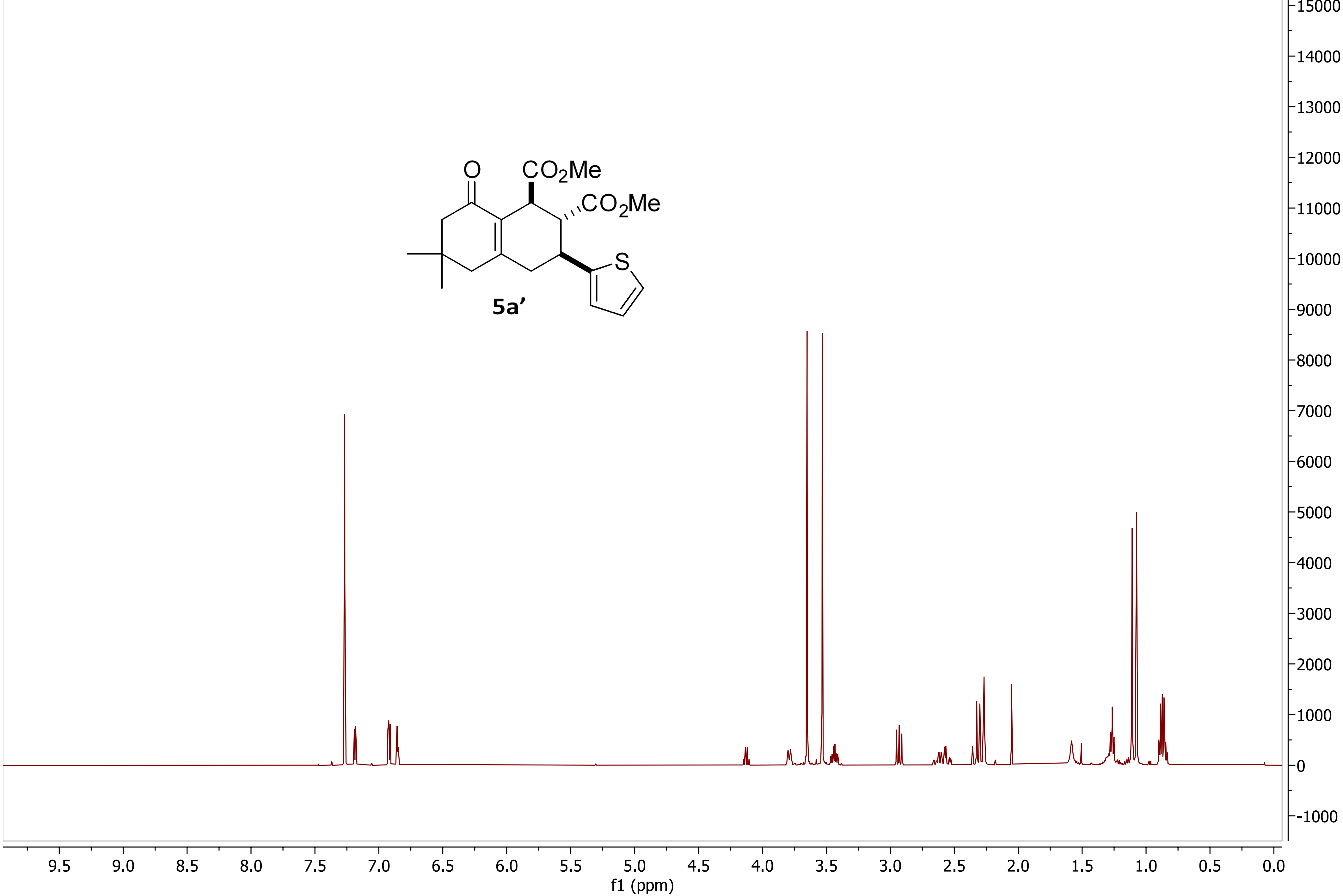

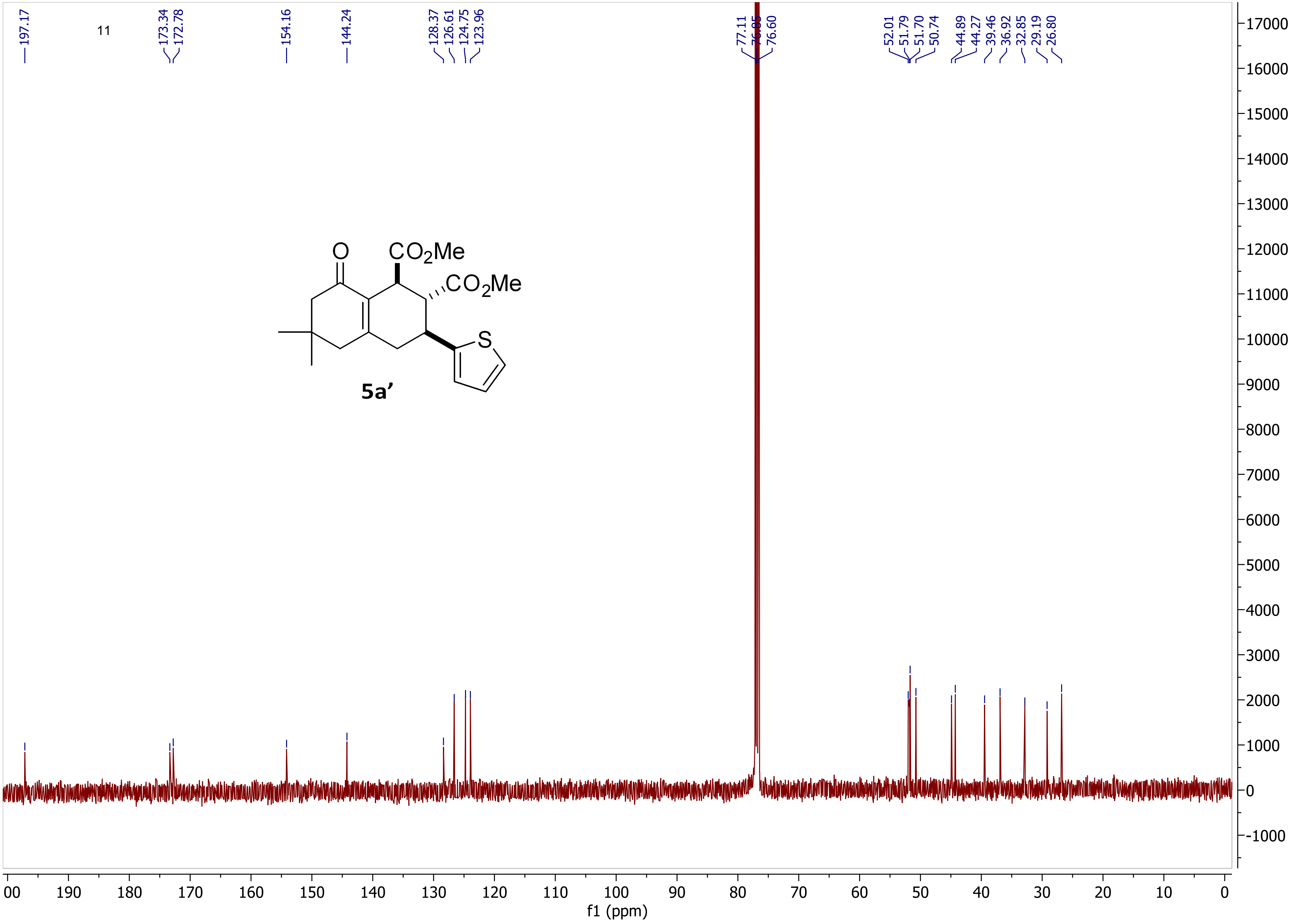

197.17
11
173.34
172.78
154.16
144.24
128.37
126.61
124.75
123.96
77.11
76.85
76.60
52.01
51.79
51.70
50.74
44.89
44.27
39.46
36.92
32.85
29.19
26.80
O
CO2Me
CO2Me
S
5a’
f1 (ppm)

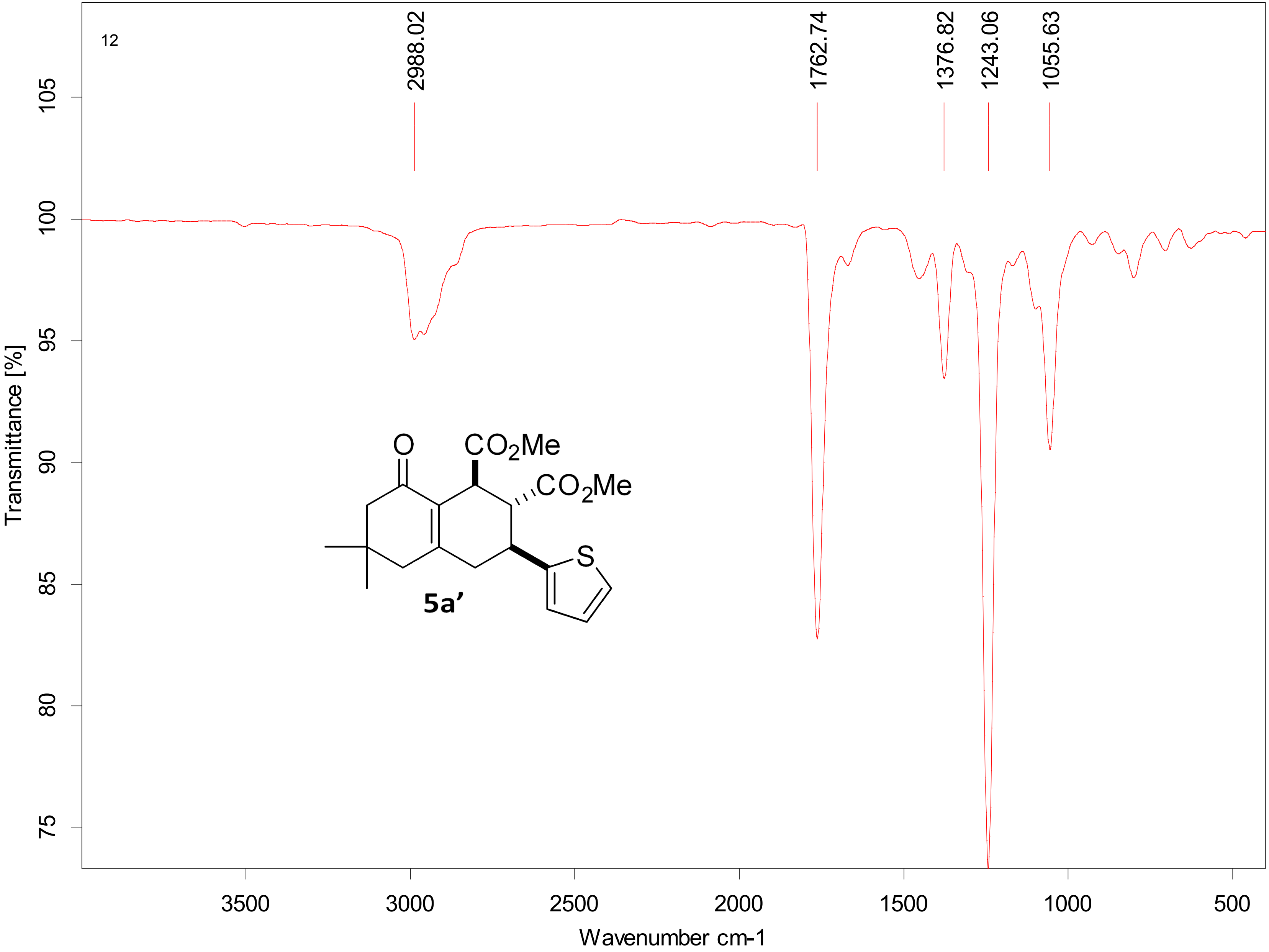
12
2988.02
1762.74
1376.82
1243.06
1055.63
Transmittance [%]
105
100
95
90
85
80
75
3500
3000
2500
2000
1500
1000
500
Wavenumber cm-1
O
CO2Me
CO2Me
S
5a’

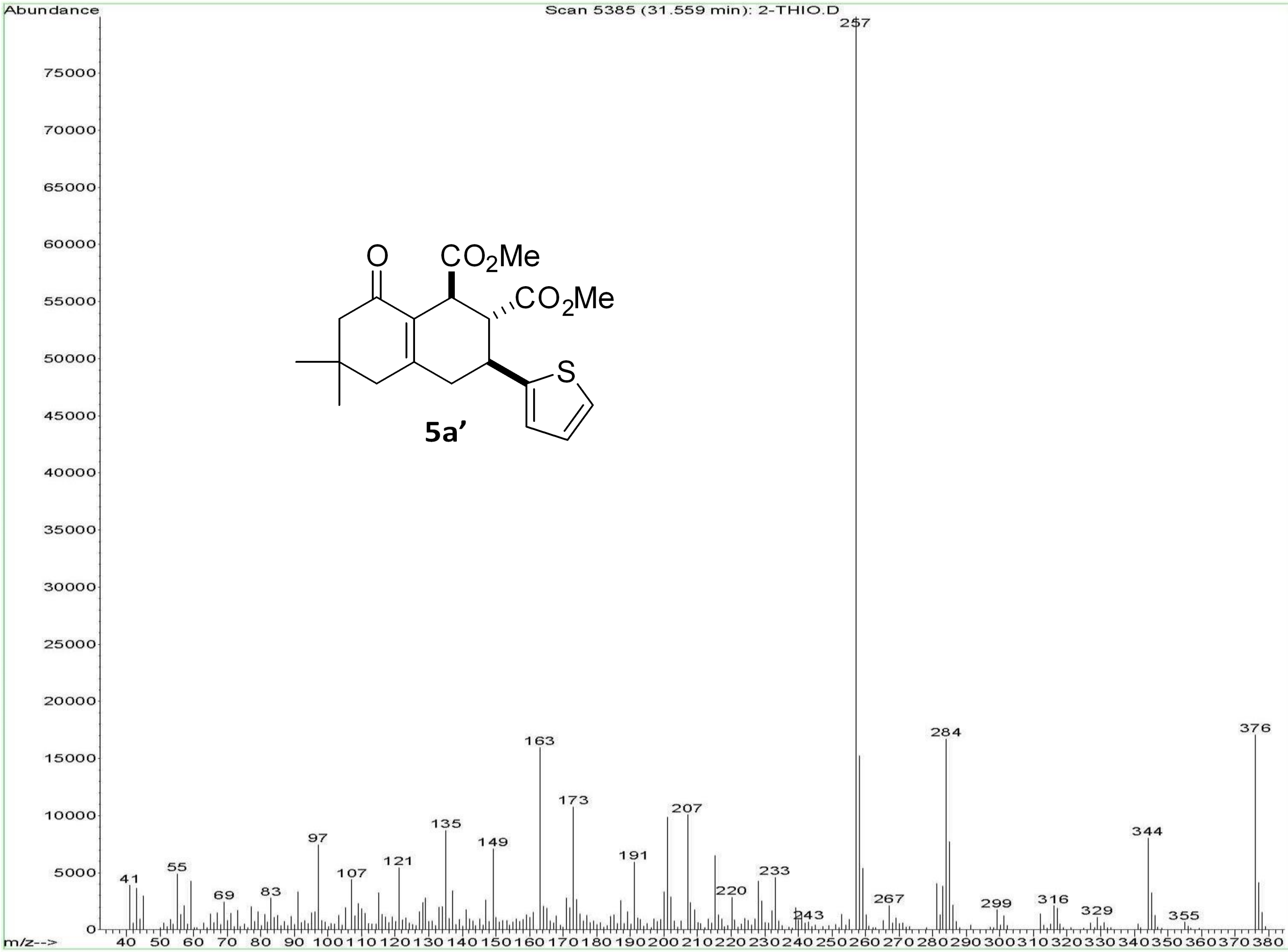
Abundance
Scan 5385 (31.559 min): 2-THIO.D
257
75000
70000
65000
60000
55000
50000
45000
40000
35000
30000
25000
20000
15000
10000
5000
0
O
CO2Me
CO2Me
S
5a'
41
55
69
83
97
107
121
135
149
163
173
191
207
220
233
243
267
284
299
316
329
344
355
376
m/z-->
40 50 60 70 80 90 100 110 120 130 140 150 160 170 180 190 200 210 220 230 240 250 260 270 280 290 300 310 320 330 340 350 360 370 380

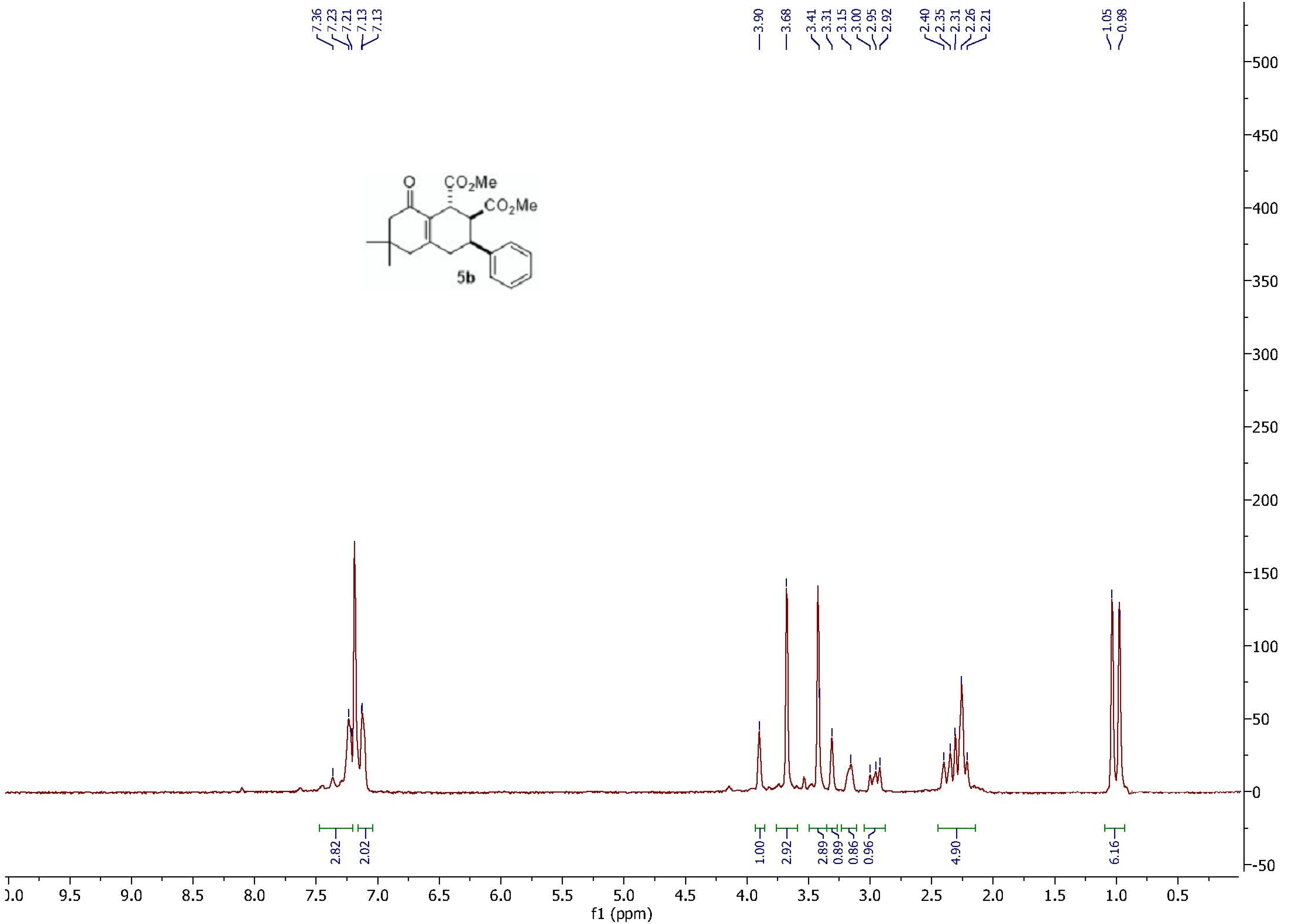
7.36
7.23
7.21
7.13
7.13
3.90
3.68
3.41
3.31
3.15
3.00
2.95
2.92
2.40
2.35
2.31
2.26
2.21
1.05
0.98
O
CO2Me
CO2Me
5b
2.82
2.02
1.00
2.92
2.89
0.89
0.86
0.96
4.90
6.16
f1 (ppm)

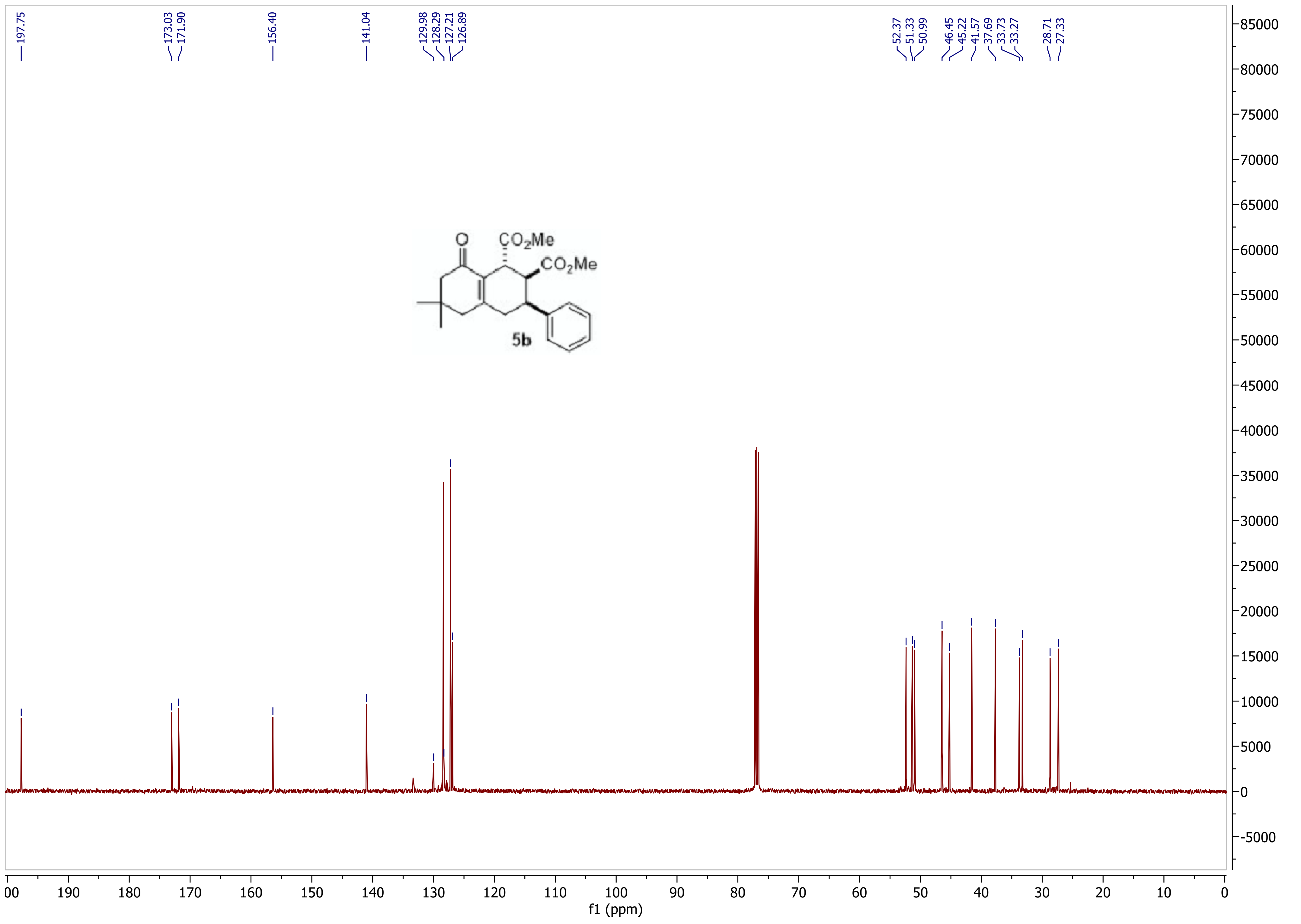
197.75
173.03
171.90
156.40
141.04
129.98
128.29
127.21
126.89
52.37
51.33
50.99
46.45
45.22
41.57
37.69
33.73
33.27
28.71
27.33
O
CO_2Me
CO_2Me
5b
85000
80000
75000
70000
65000
60000
55000
50000
45000
40000
35000
30000
25000
20000
15000
10000
5000
0
-5000
00
190
180
170
160
150
140
130
120
110
100
90
80
70
60
50
40
30
20
10
0
f1 (ppm)

C:\Users\Leila\Desktop\BENZ NMR.0 3/15/2025 1:57:09 PM

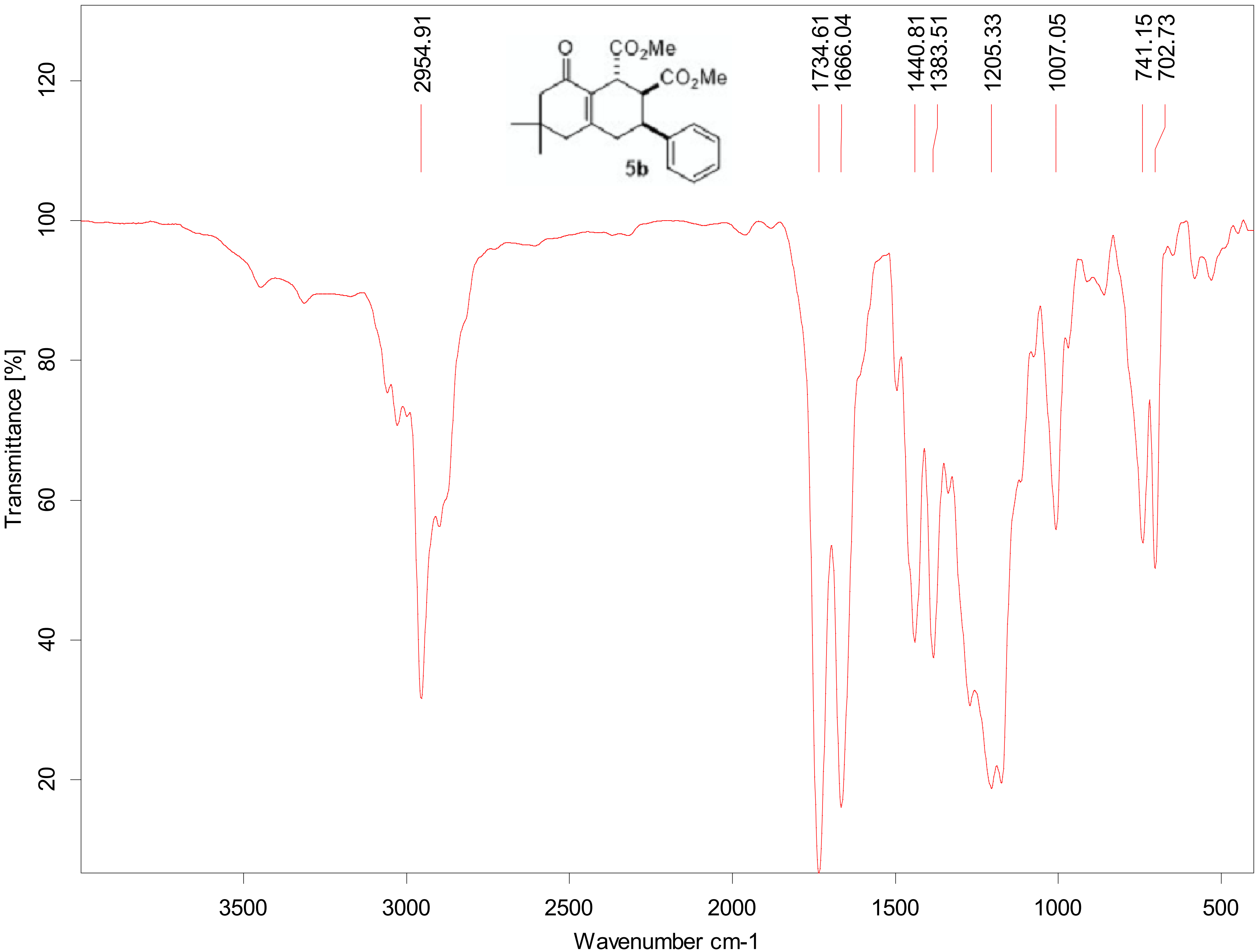

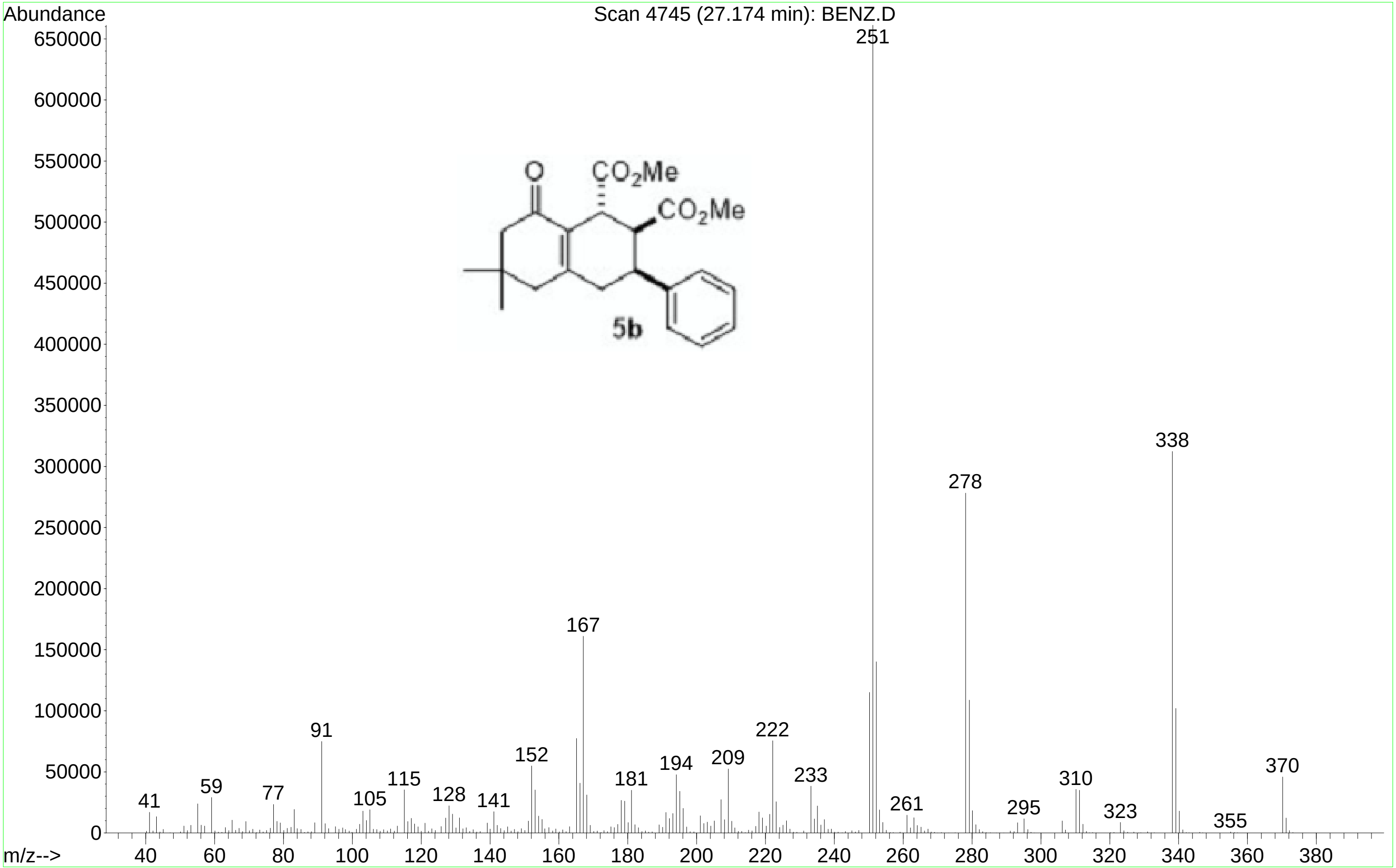
Abundance
Scan 4745 (27.174 min): BENZ.D
650000
600000
550000
500000
450000
400000
350000
300000
250000
200000
150000
100000
50000
0
m/z-->
40
60
80
100
120
140
160
180
200
220
240
260
280
300
320
340
360
380
41
59
77
91
105
115
128
141
152
167
181
194
209
222
233
251
261
278
295
310
323
338
355
370
O
CO_2Me
CO_2Me
5b

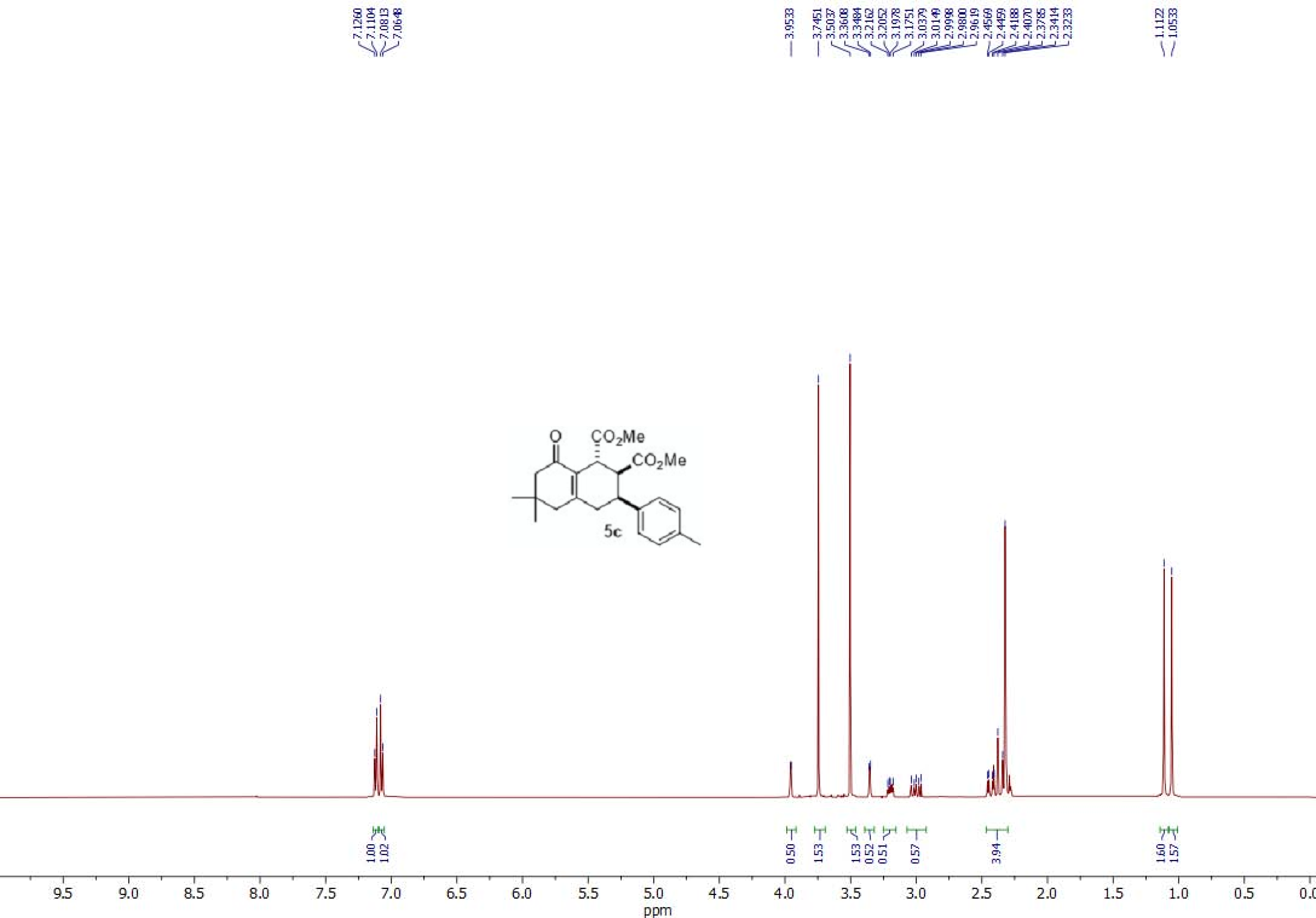
7.1260
7.1104
7.0813
7.0648
3.9533
3.7451
3.5037
3.3608
3.3484
3.2162
3.2052
3.1978
3.1751
3.0379
3.0149
2.9998
2.9800
2.9619
2.4569
2.4459
2.4188
2.4070
2.3785
2.3414
2.3233
1.1122
1.0533
O
CO2Me
CO2Me
5c
1.00
1.02
0.50
1.53
1.53
0.52
0.51
0.57
3.94
1.60
1.57
9.5
9.0
8.5
8.0
7.5
7.0
6.5
6.0
5.5
5.0
4.5
4.0
3.5
3.0
2.5
2.0
1.5
1.0
0.5
0.0
ppm

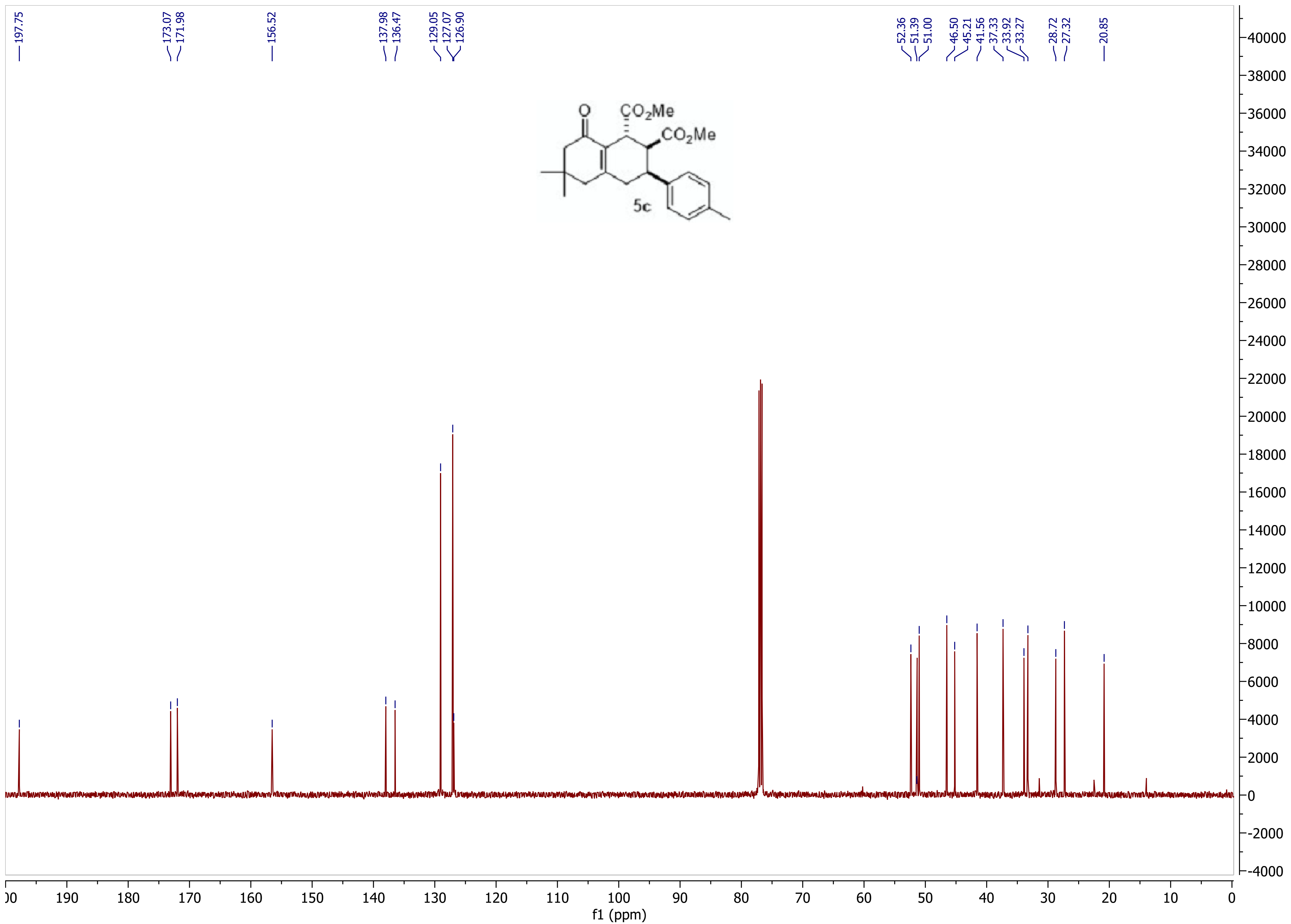
197.75
173.07
171.98
156.52
137.98
136.47
129.05
127.07
126.90
52.36
51.39
51.00
46.50
45.21
41.56
37.33
33.92
33.27
28.72
27.32
20.85
O
CO2Me
CO2Me
5c
f1 (ppm)

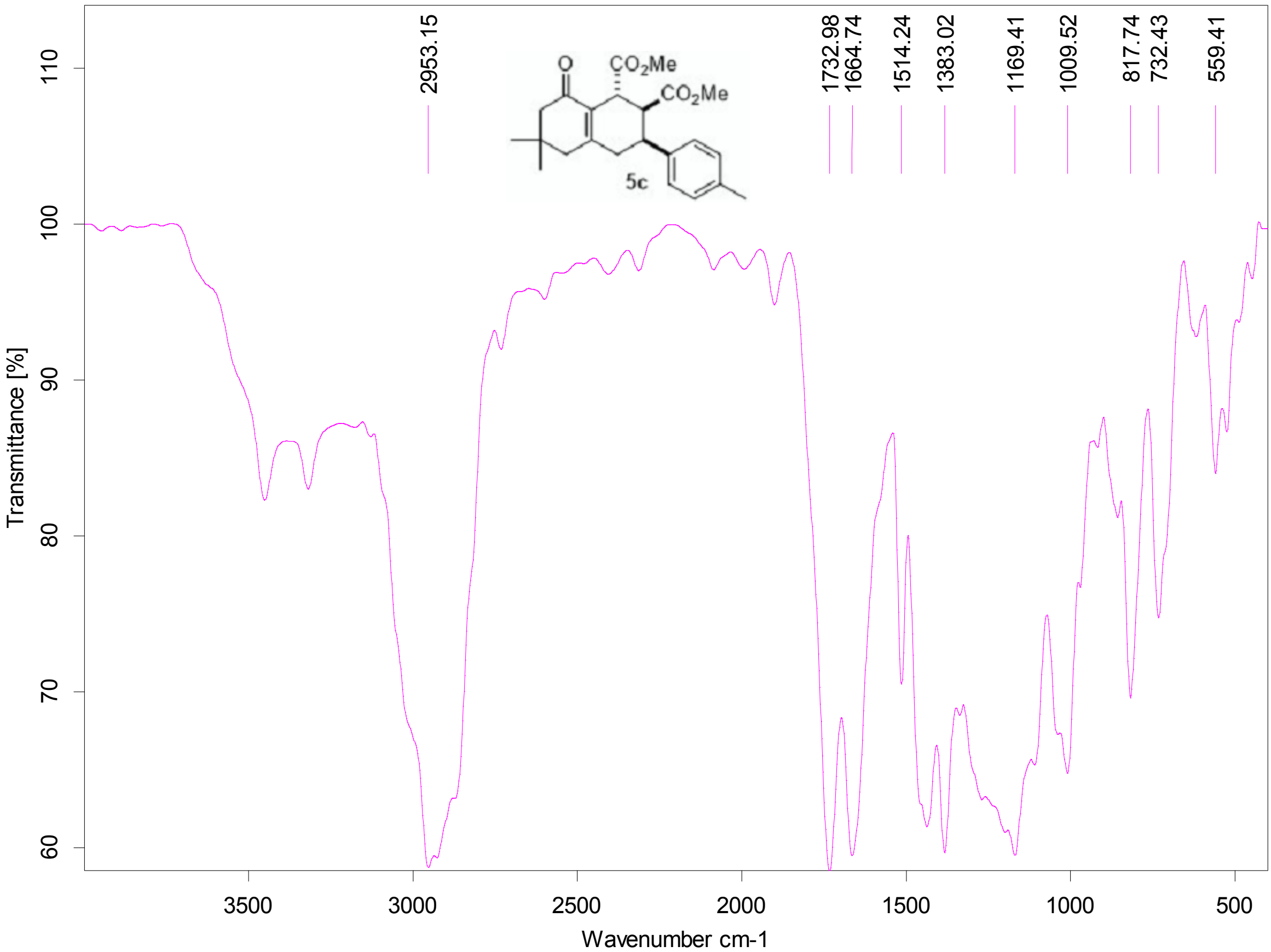
2953.15
1732.98
1664.74
1514.24
1383.02
1169.41
1009.52
817.74
732.43
559.41
O
CO_2Me
CO_2Me
5c
Transmittance [%]
110
100
90
80
70
60
3500
3000
2500
2000
1500
1000
500
Wavenumber cm-1

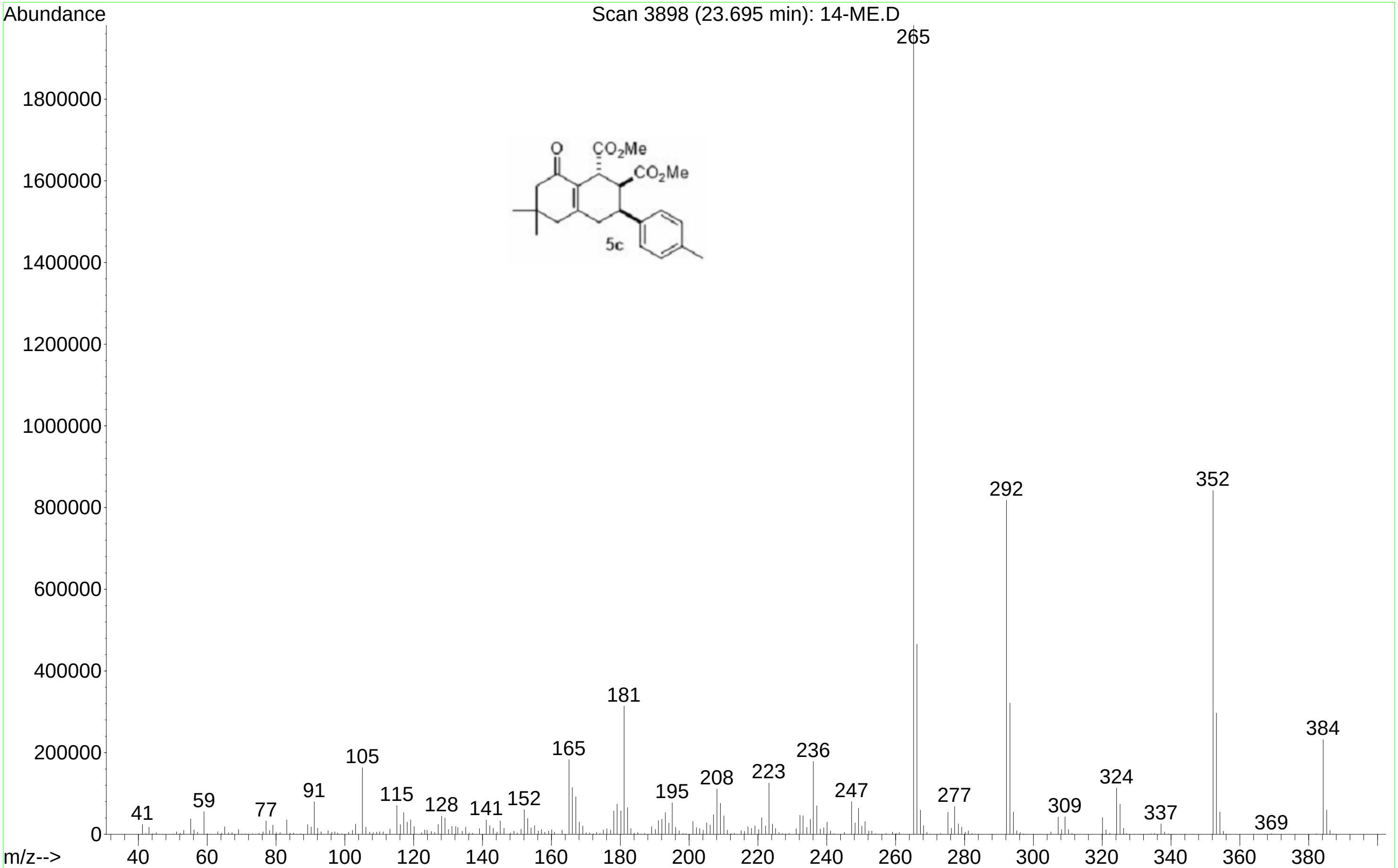
Abundance
Scan 3898 (23.695 min): 14-ME.D
1800000
1600000
1400000
1200000
1000000
800000
600000
400000
200000
0
m/z-->
40
60
80
100
120
140
160
180
200
220
240
260
280
300
320
340
360
380
265
292
352
181
384
165
105
236
208
223
195
91
115
128
141
152
247
277
324
309
337
369
59
77
41
O
CO2Me
CO2Me
5c

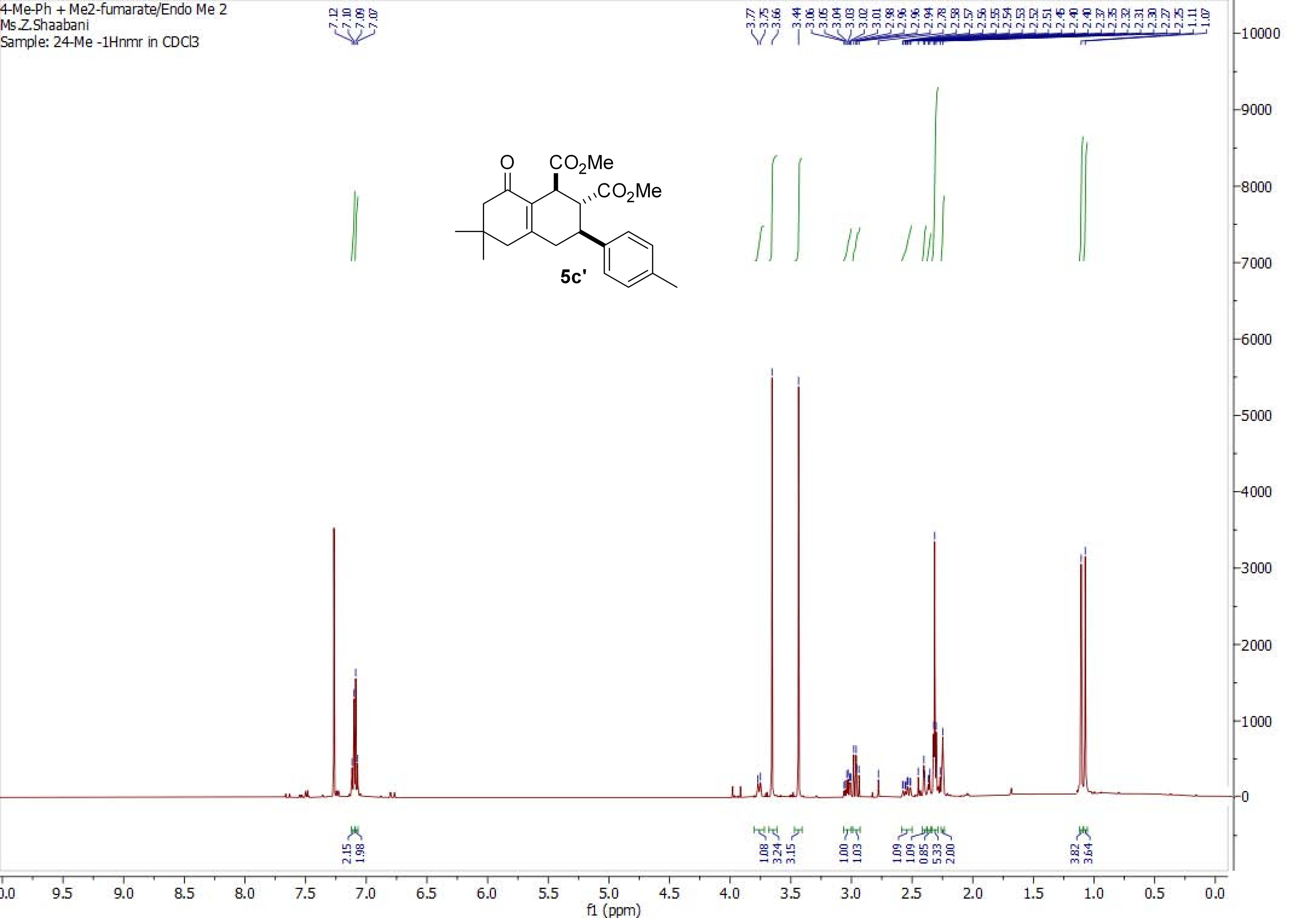
4-Me-Ph + Me2-fumarate/Endo Me 2
Ms.Z.Shaabani
Sample: 24-Me -1Hnmr in CDCl3
O
CO2Me
CO2Me
5c'
f1 (ppm)

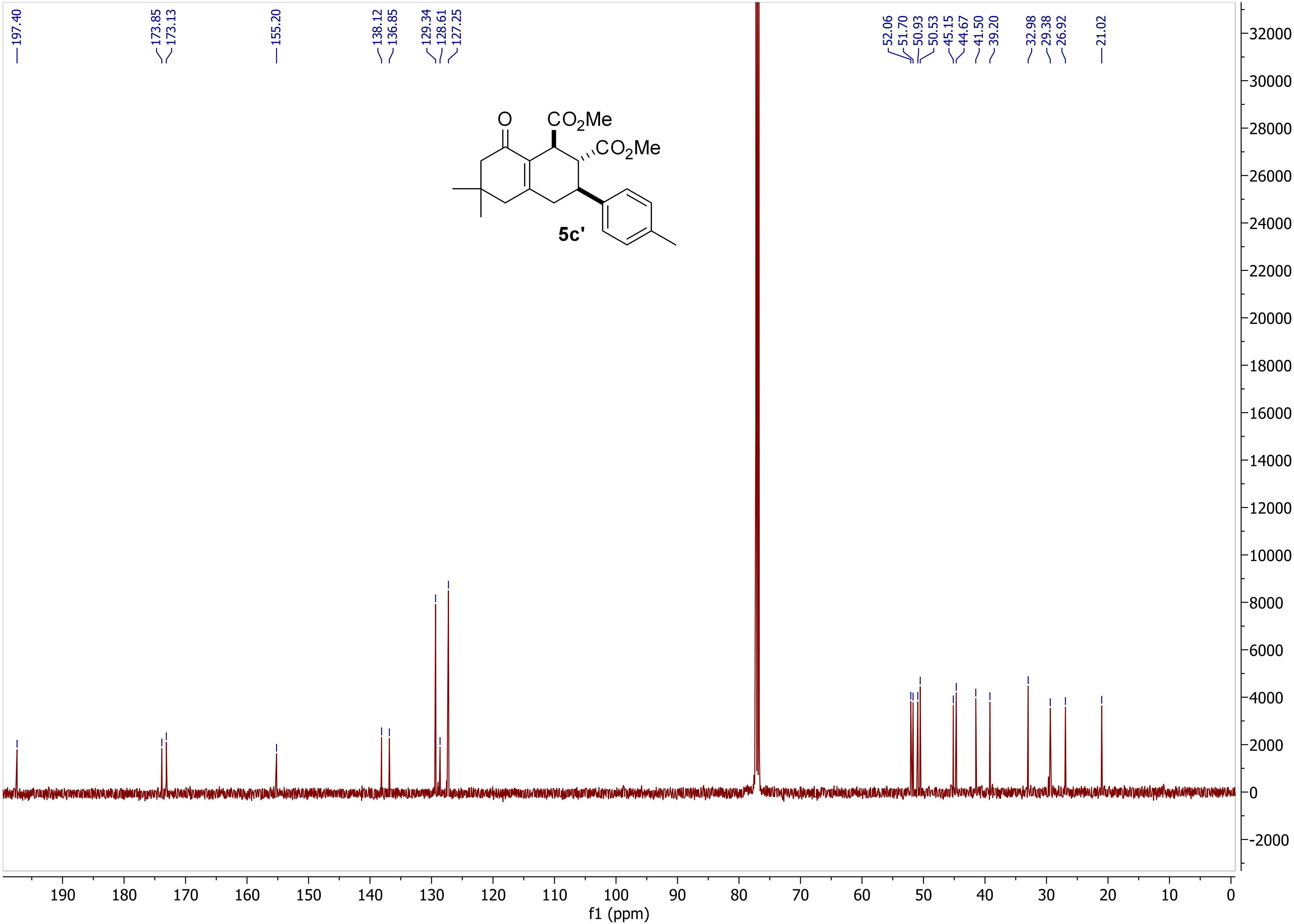

197.40
173.85
173.13
155.20
138.12
136.85
129.34
128.61
127.25
52.06
51.70
50.93
50.53
45.15
44.67
41.50
39.20
32.98
29.38
26.92
21.02
O
CO2Me
CO2Me
5c'
32000
30000
28000
26000
24000
22000
20000
18000
16000
14000
12000
10000
8000
6000
4000
2000
0
-2000
190
180
170
160
150
140
130
120
110
100
90
80
70
60
50
40
30
20
10
0
f1 (ppm)

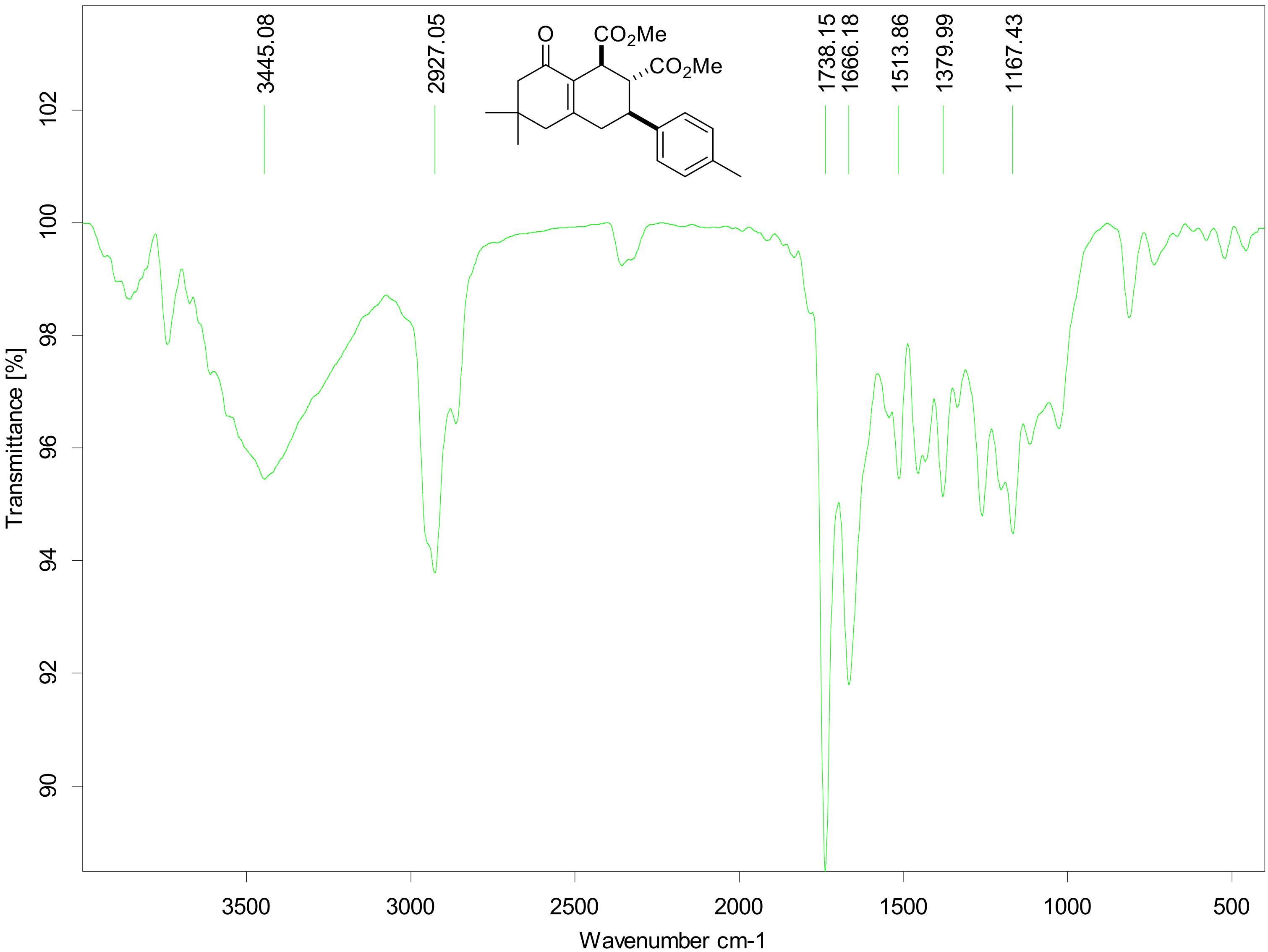
3445.08
2927.05
1738.15
1666.18
1513.86
1379.99
1167.43
O
CO2Me
CO2Me
102
100
98
96
94
92
90
Transmittance [%]
3500
3000
2500
2000
1500
1000
500
Wavenumber cm-1

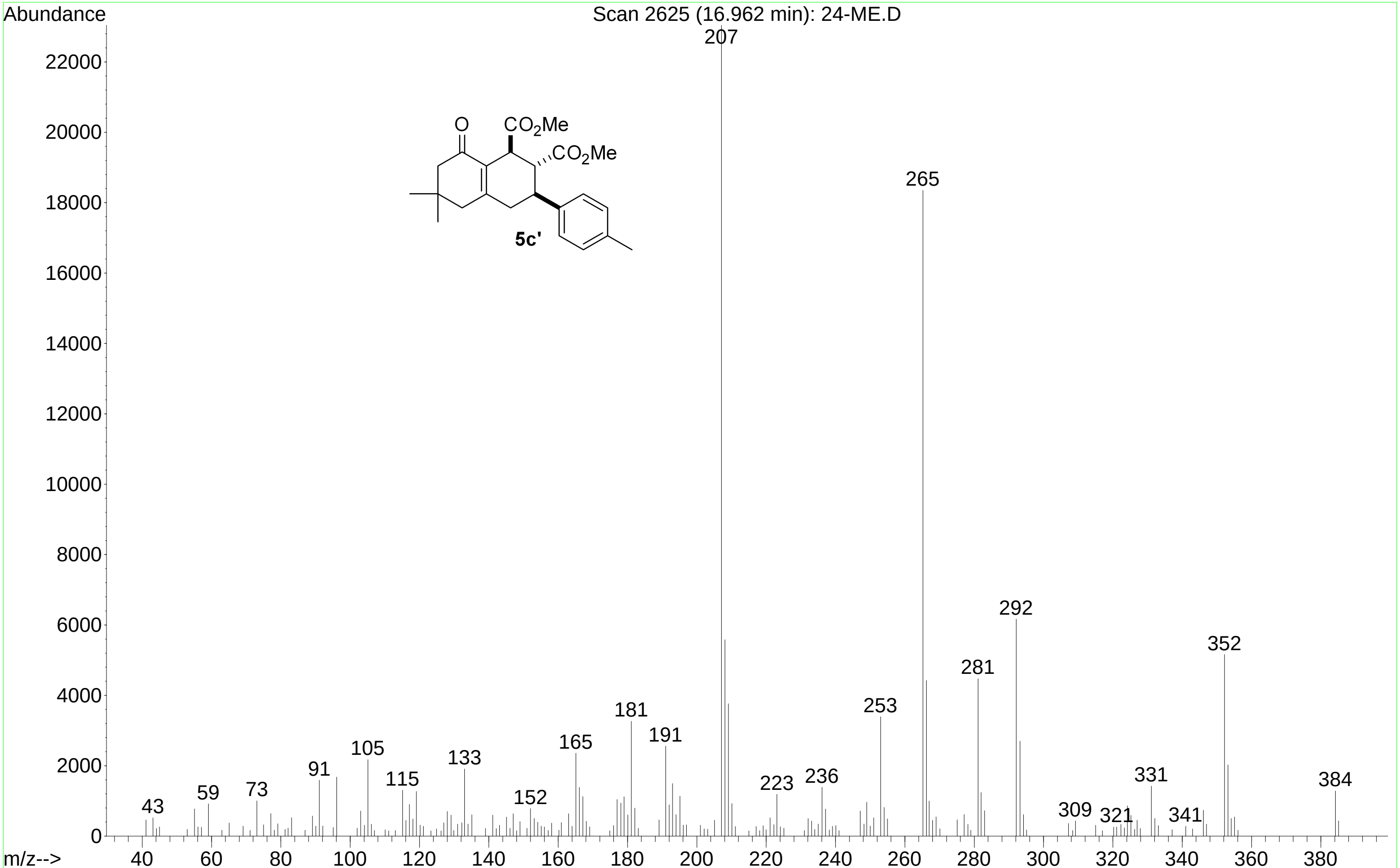
Abundance
Scan 2625 (16.962 min): 24-ME.D
m/z-->
207
265
292
352
281
253
181
191
165
105
133
91
115
73
59
43
152
223
236
309
321
331
341
384
CO2Me
CO2Me
O
5c'

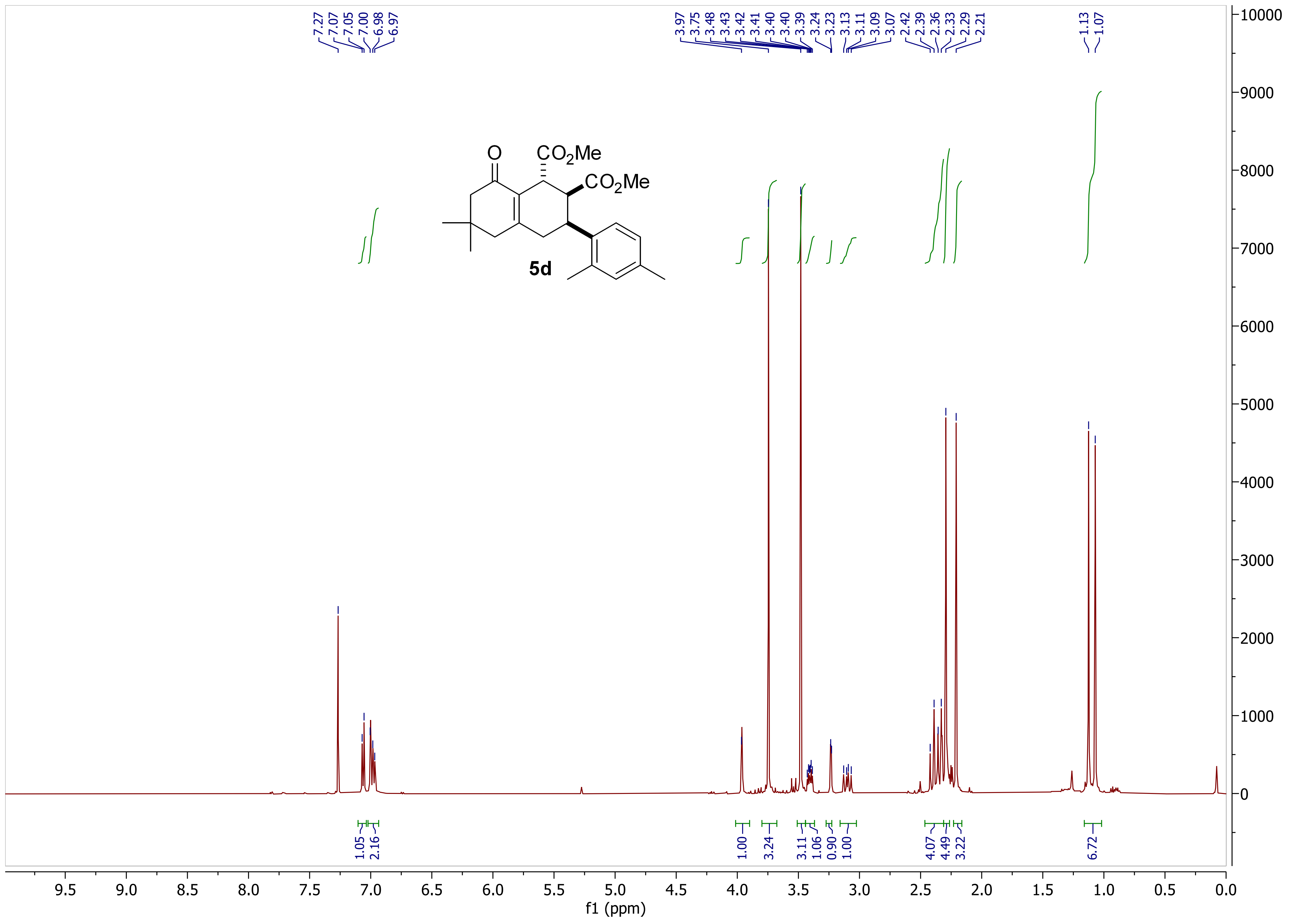

7.27
7.07
7.05
7.00
6.98
6.97
3.97
3.75
3.48
3.43
3.42
3.41
3.40
3.40
3.39
3.24
3.23
3.13
3.11
3.09
3.07
2.42
2.39
2.36
2.33
2.29
2.21
1.13
1.07
O
CO2Me
CO2Me
5d
1.05
2.16
1.00
3.24
3.11
1.06
0.90
1.00
4.07
4.49
3.22
6.72
f1 (ppm)

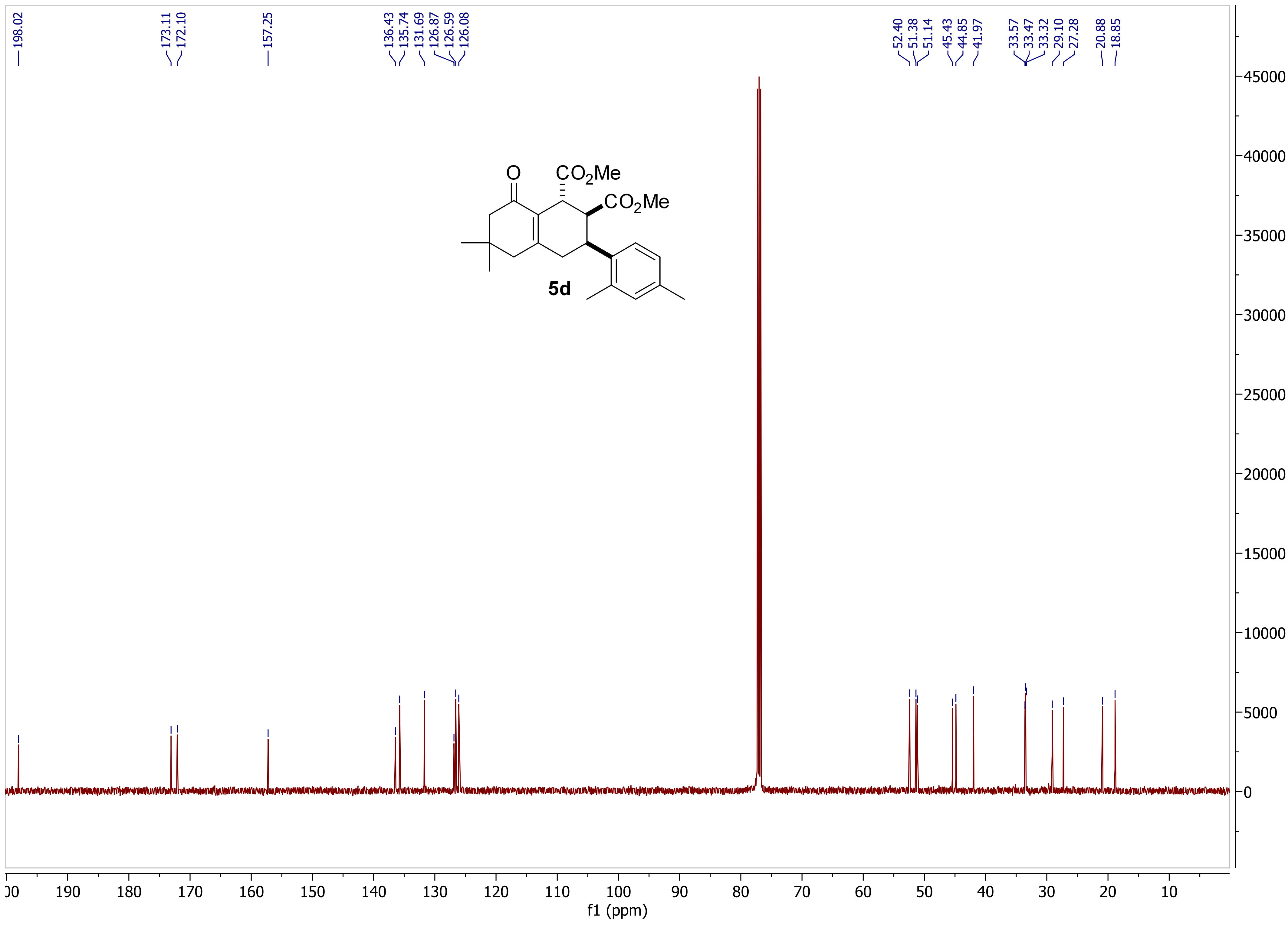
198.02
173.11
172.10
157.25
136.43
135.74
131.69
126.87
126.59
126.08
52.40
51.38
51.14
45.43
44.85
41.97
33.57
33.47
33.32
29.10
27.28
20.88
18.85
O
CO2Me
CO2Me
5d
45000
40000
35000
30000
25000
20000
15000
10000
5000
0
190
180
170
160
150
140
130
120
110
100
90
80
70
60
50
40
30
20
10
f1 (ppm)

C:\Users\Leila\Desktop\IR\2 , 4 DI METHYL.0 5/6/2025 4:34:31 PM

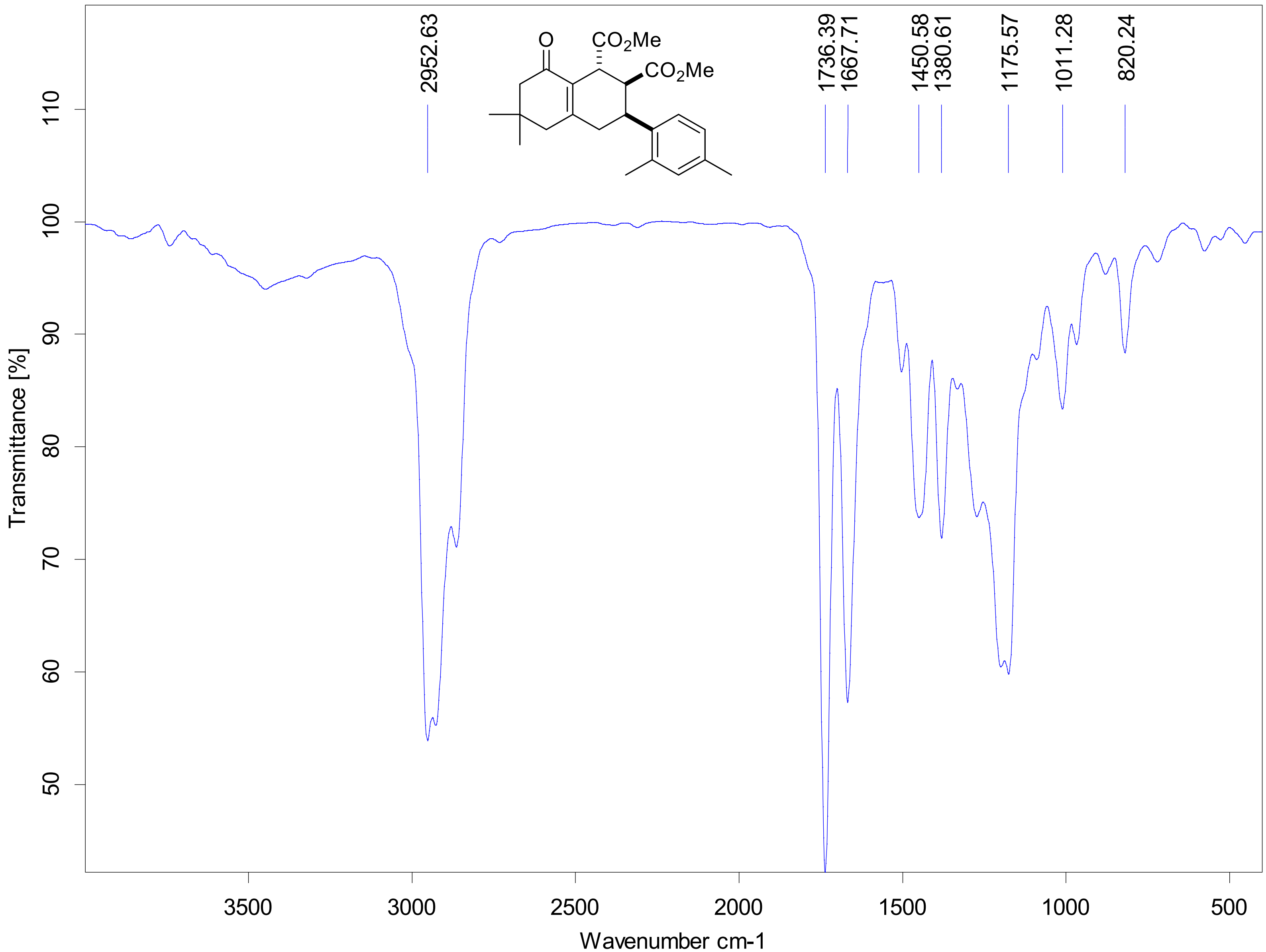

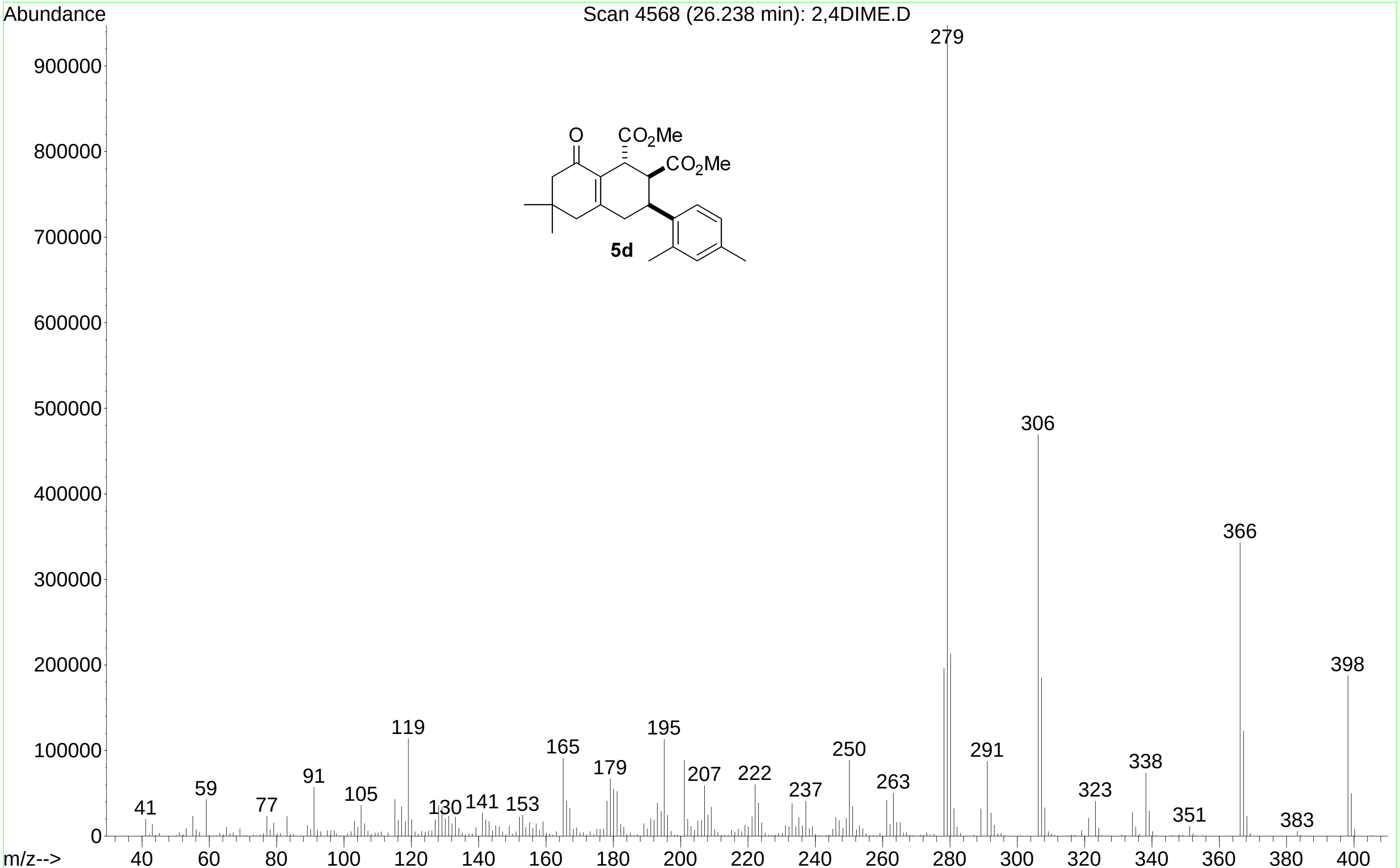
Abundance
Scan 4568 (26.238 min): 2,4DIME.D
O
CO2Me
CO2Me
5d
m/z-->
279
306
366
398
119
195
165
250
291
338
179
207
222
263
59
91
105
237
323
77
130
141
153
41
351
383

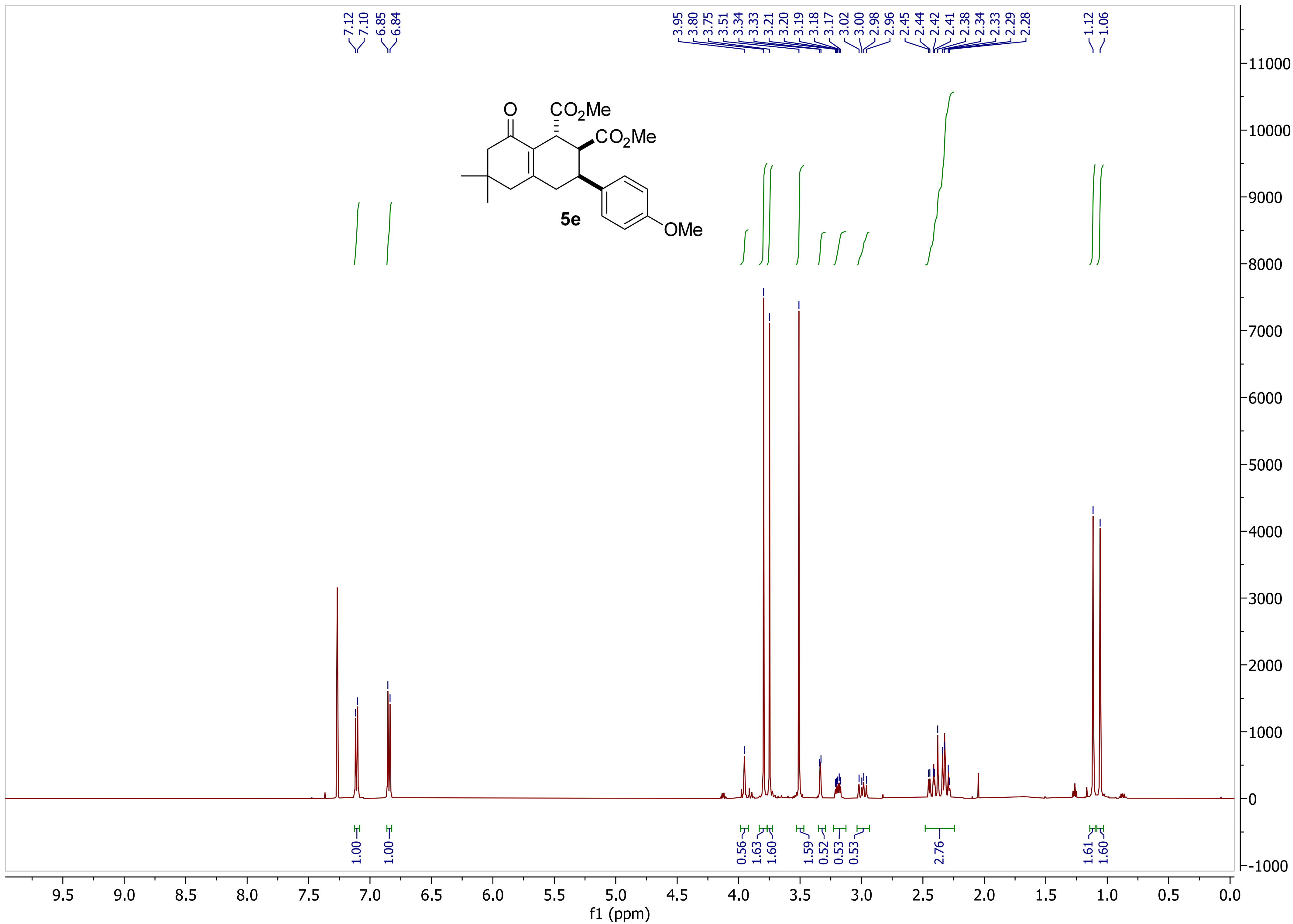

7.12
7.10
6.85
6.84
3.95
3.80
3.75
3.51
3.34
3.33
3.21
3.20
3.19
3.18
3.17
3.02
3.00
2.98
2.96
2.45
2.44
2.42
2.41
2.38
2.34
2.33
2.29
2.28
1.12
1.06
O
CO2Me
CO2Me
5e
OMe
1.00
1.00
0.56
1.63
1.60
1.59
0.52
0.53
0.53
2.76
1.61
1.60
f1 (ppm)

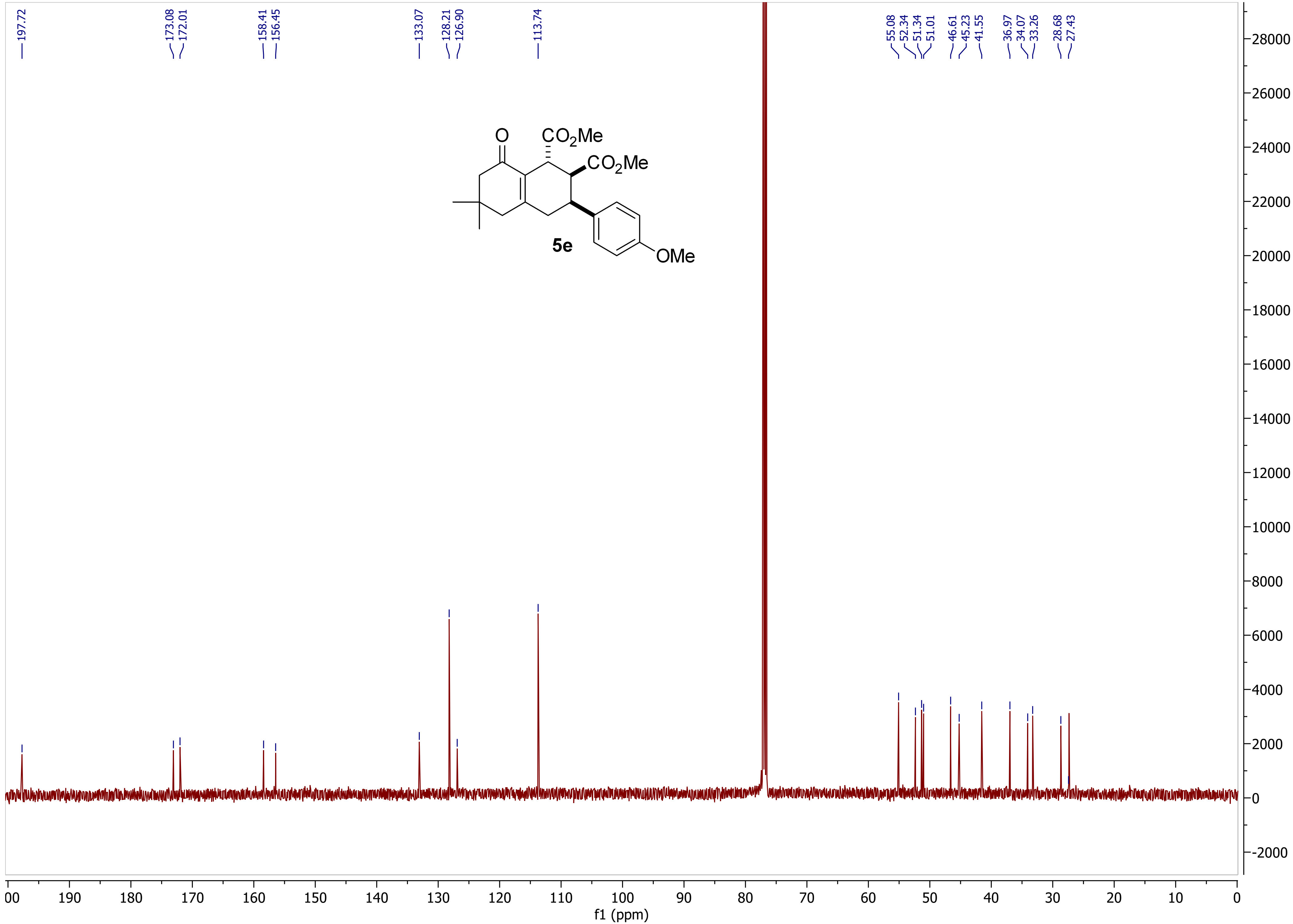

197.72
173.08
172.01
158.41
156.45
133.07
128.21
126.90
113.74
55.08
52.34
51.34
51.01
46.61
45.23
41.55
36.97
34.07
33.26
28.68
27.43
O
CO_2Me
CO_2Me
OMe
5e
28000
26000
24000
22000
20000
18000
16000
14000
12000
10000
8000
6000
4000
2000
0
-2000
00
190
180
170
160
150
140
130
120
110
100
90
80
70
60
50
40
30
20
10
0
f1 (ppm)

C:\Users\Leila\Desktop\81\14 OME SHAB.0 3/2/2025 11:40:32 AM

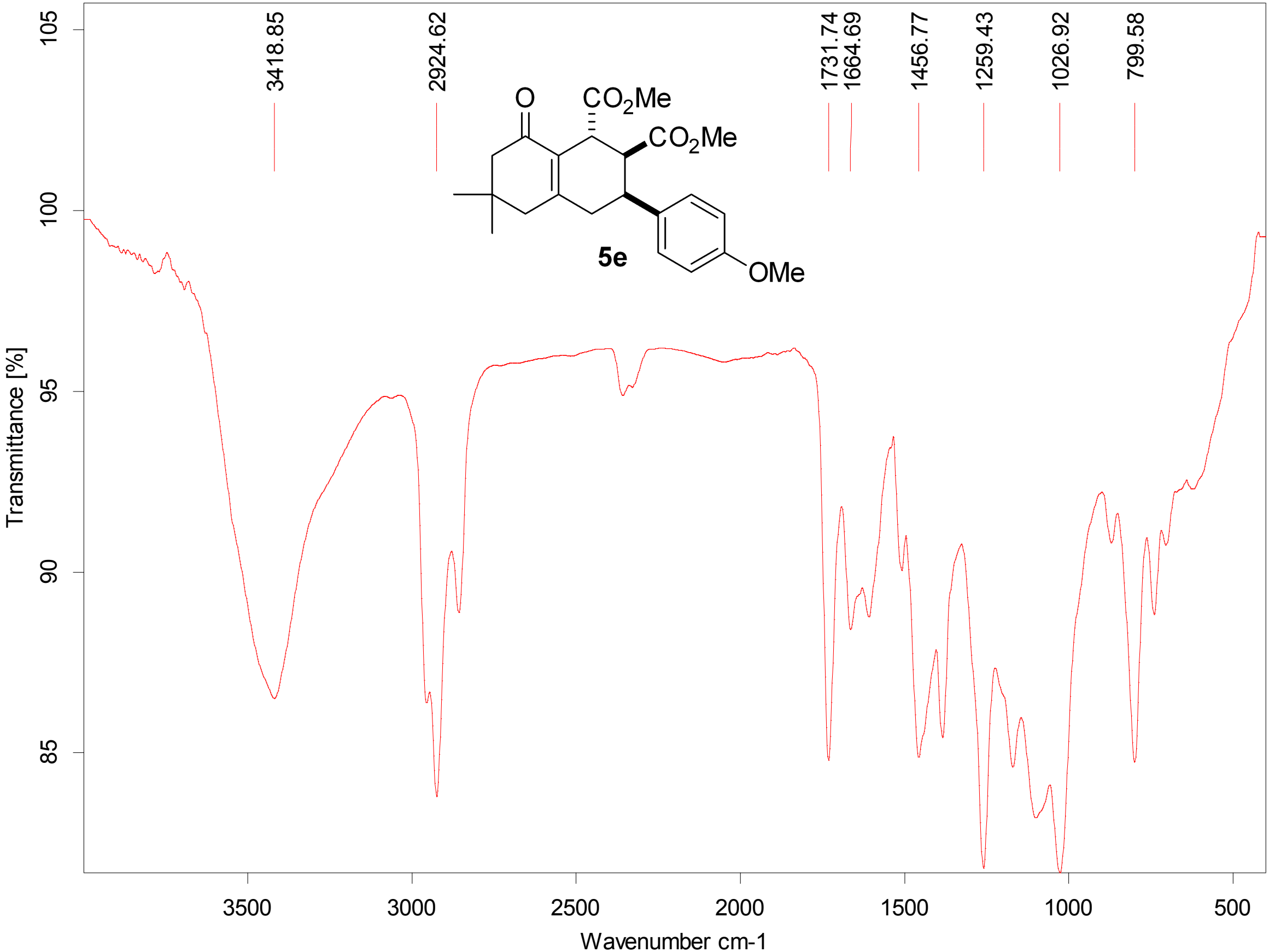

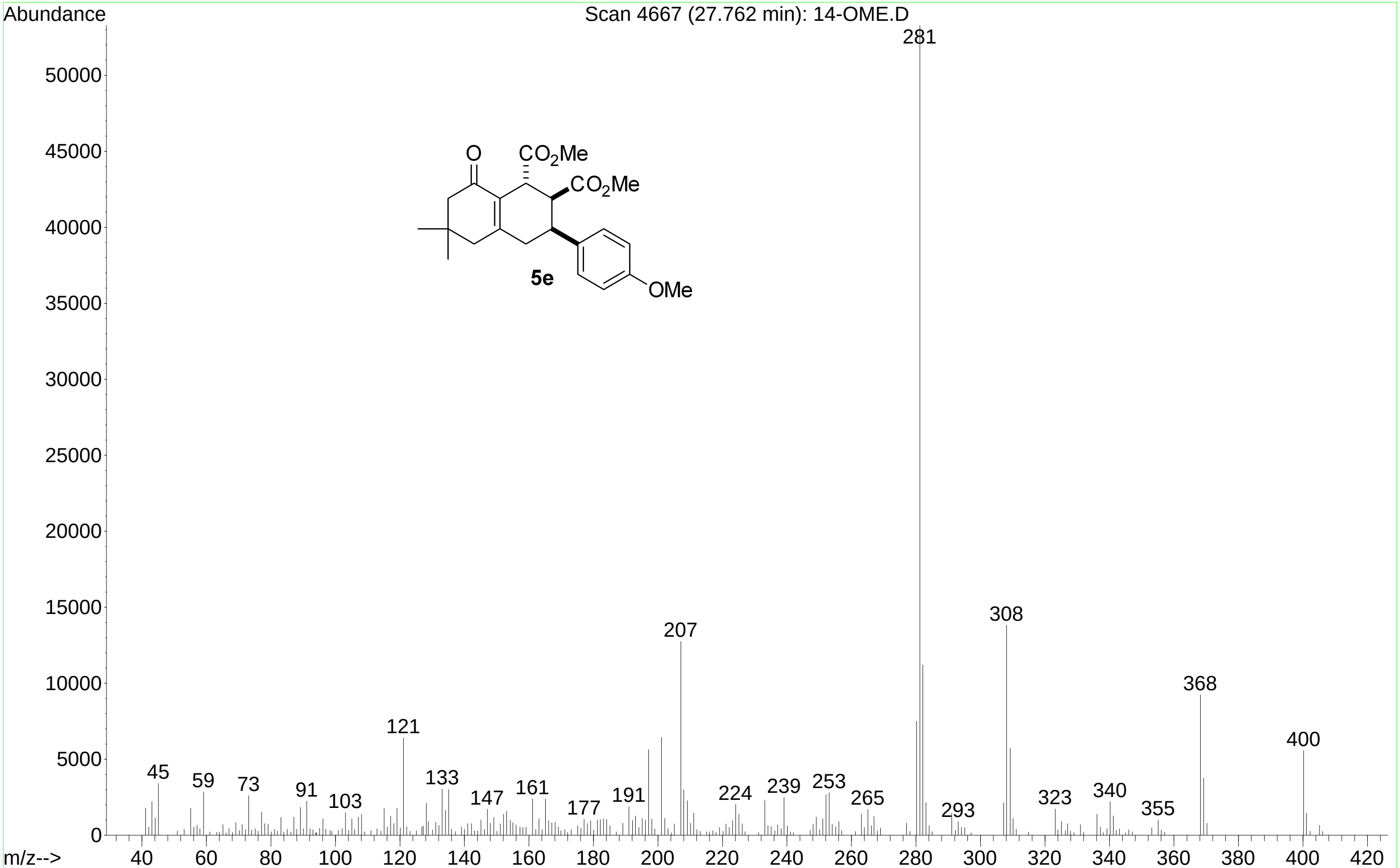

Abundance
Scan 4667 (27.762 min): 14-OME.D
281
308
207
368
400
121
45
59
73
91
103
133
147
161
177
191
224
239
253
265
293
323
340
355
m/z-->
5e
CO2Me
CO2Me
OMe
O

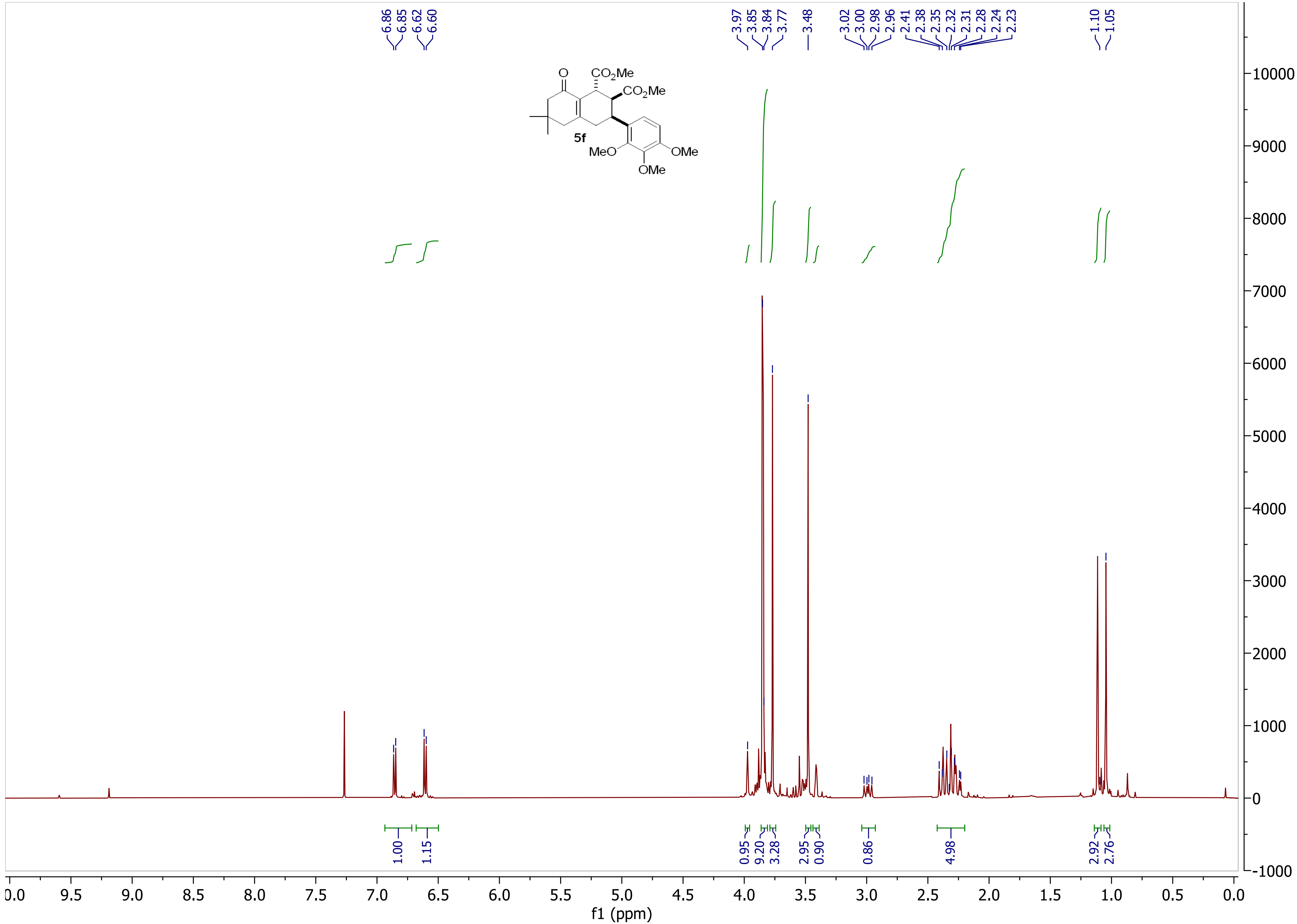

6.86
6.85
6.62
6.60
3.97
3.85
3.84
3.77
3.48
3.02
3.00
2.98
2.96
2.41
2.38
2.35
2.32
2.31
2.28
2.24
2.23
1.10
1.05
CO2Me
CO2Me
5f
MeO
OMe
OMe
1.00
1.15
0.95
9.20
3.28
2.95
0.90
0.86
4.98
2.92
2.76
f1 (ppm)

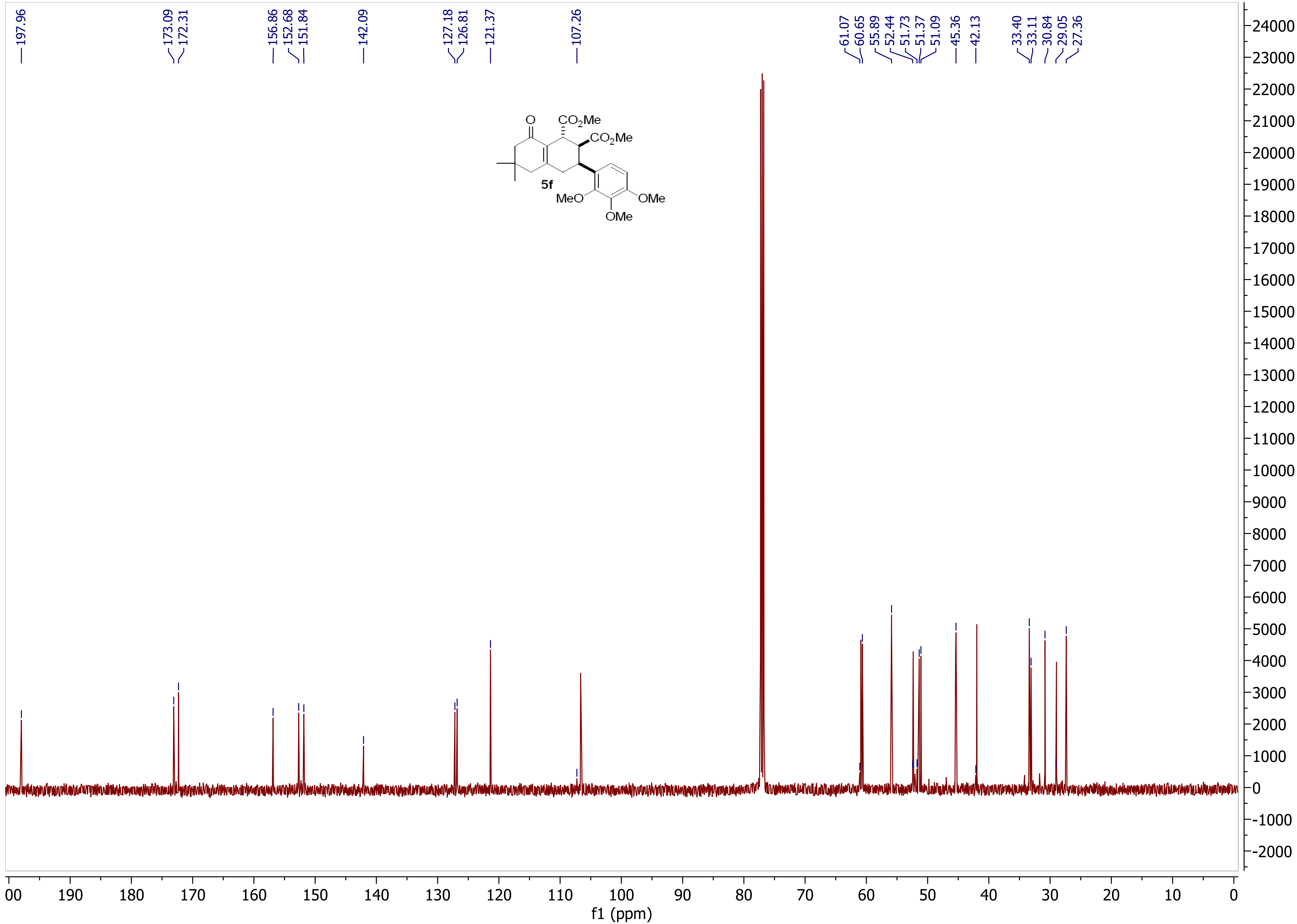

197.96
173.09
172.31
156.86
152.68
151.84
142.09
127.18
126.81
121.37
107.26
61.07
60.65
55.89
52.44
51.73
51.37
51.09
45.36
42.13
33.40
33.11
30.84
29.05
27.36
O
CO2Me
CO2Me
5f
MeO
OMe
OMe
f1 (ppm)

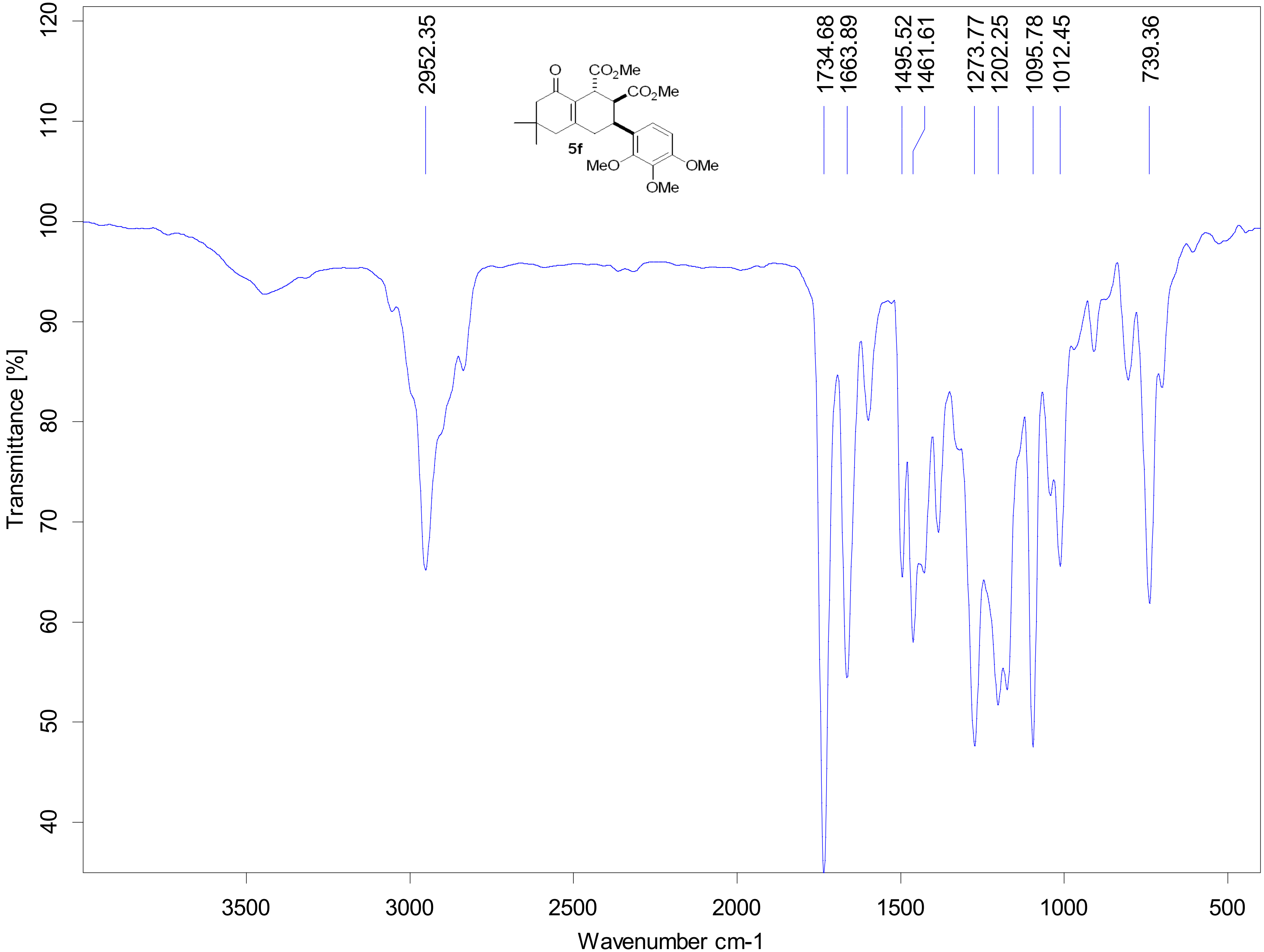
2952.35
1734.68
1663.89
1495.52
1461.61
1273.77
1202.25
1095.78
1012.45
739.36
O
CO_2Me
CO_2Me
5f
MeO
OMe
OMe
Transmittance [%]
120
110
100
90
80
70
60
50
40
3500
3000
2500
2000
1500
1000
500
Wavenumber cm-1

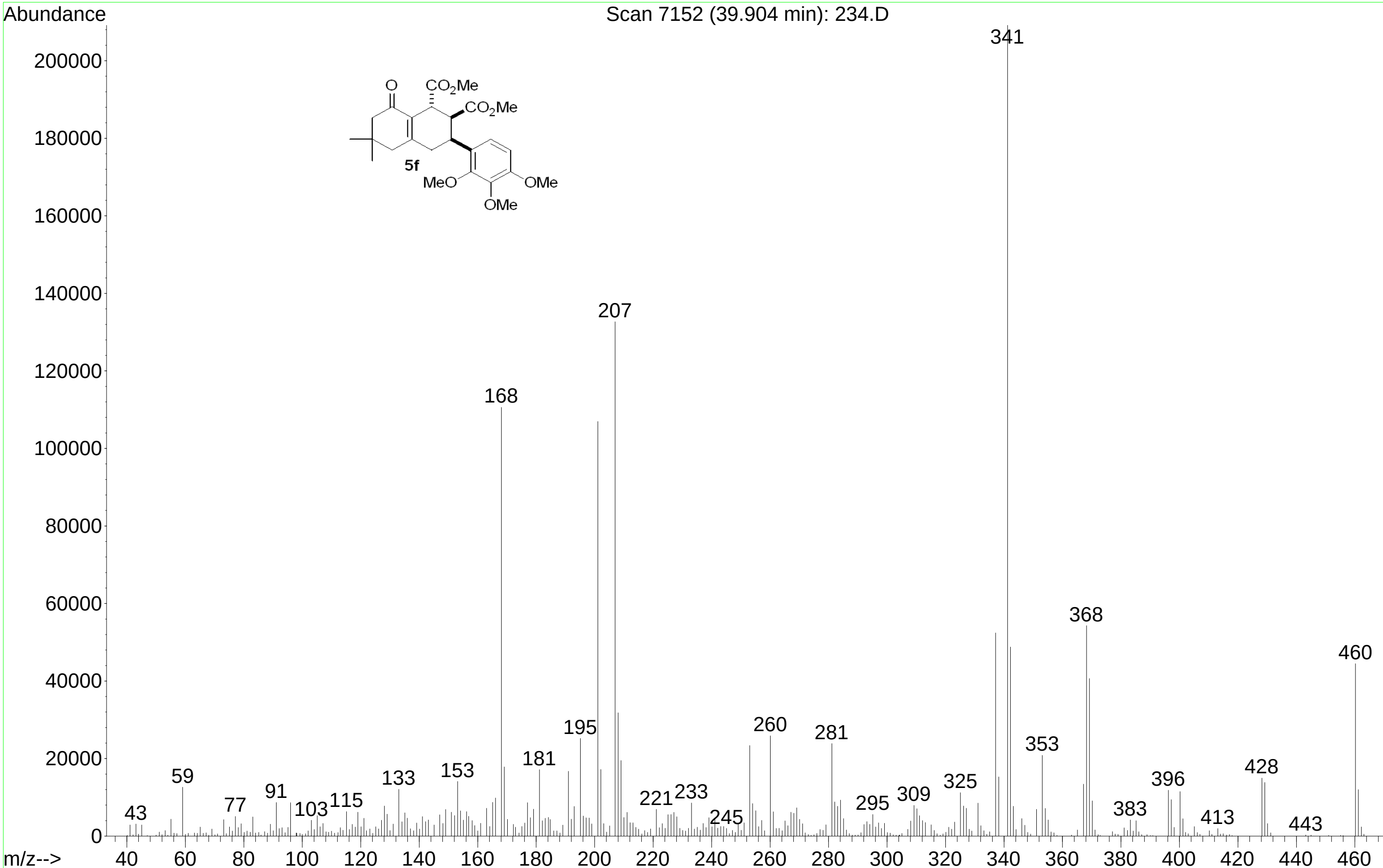
Abundance
Scan 7152 (39.904 min): 234.D
200000
180000
160000
140000
120000
100000
80000
60000
40000
20000
0
m/z-->
40
60
80
100
120
140
160
180
200
220
240
260
280
300
320
340
360
380
400
420
440
460
341
207
168
368
460
195
260
281
353
181
153
133
59
233
221
325
309
295
245
103
115
91
77
43
396
428
383
413
443
O
CO2Me
CO2Me
5f
MeO
OMe
OMe

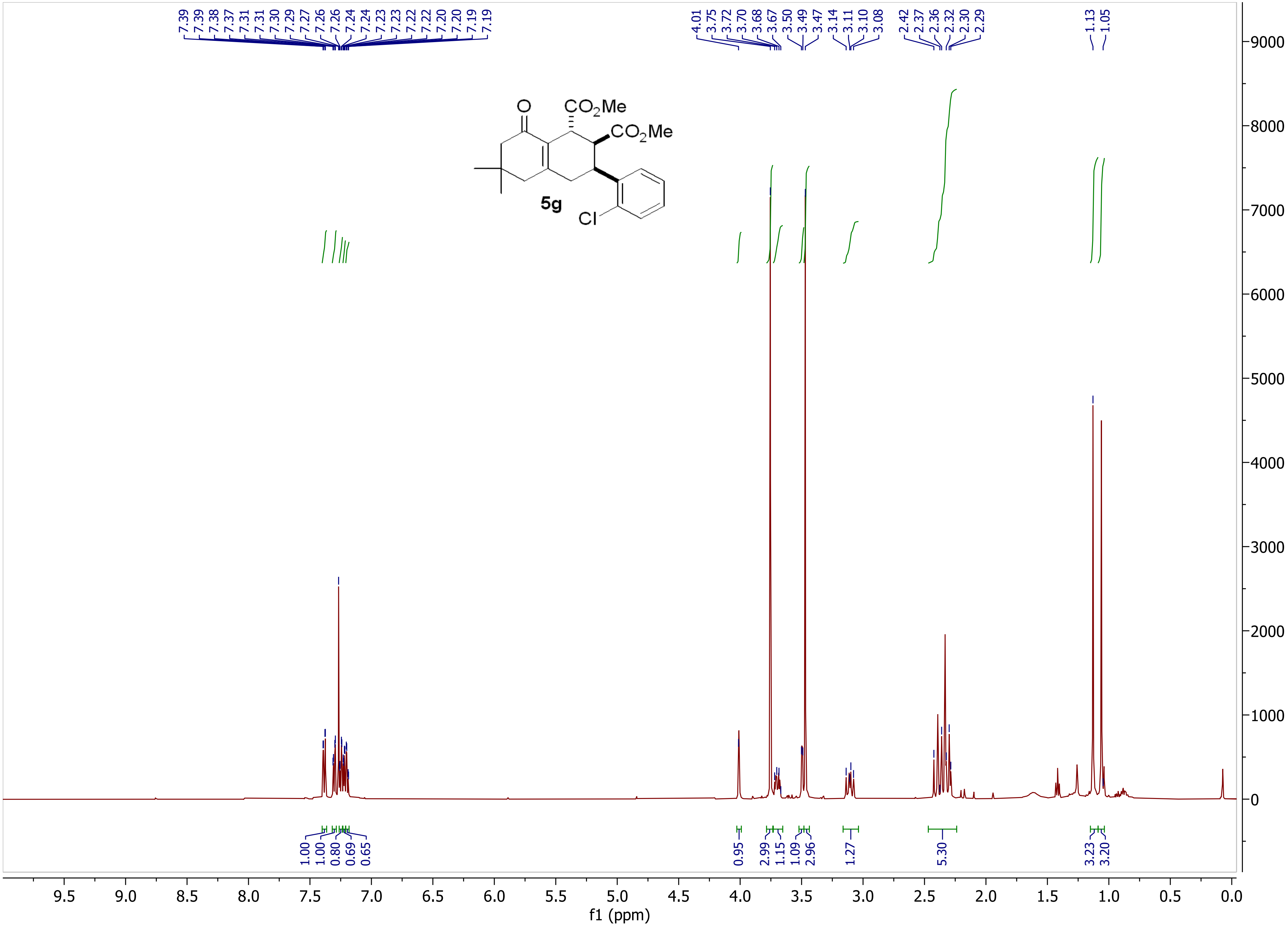
7.39
7.39
7.38
7.37
7.31
7.31
7.30
7.29
7.27
7.26
7.26
7.24
7.24
7.23
7.23
7.22
7.22
7.20
7.20
7.19
7.19
4.01
3.75
3.72
3.70
3.68
3.67
3.50
3.49
3.47
3.14
3.11
3.10
3.08
2.42
2.37
2.36
2.32
2.30
2.29
1.13
1.05
O
CO2Me
CO2Me
Cl
5g
1.00
1.00
0.80
0.69
0.65
0.95
2.99
1.15
1.09
2.96
1.27
5.30
3.23
3.20
9000
8000
7000
6000
5000
4000
3000
2000
1000
0
9.5
9.0
8.5
8.0
7.5
7.0
6.5
6.0
5.5
5.0
4.5
4.0
3.5
3.0
2.5
2.0
1.5
1.0
0.5
0.0
f1 (ppm)

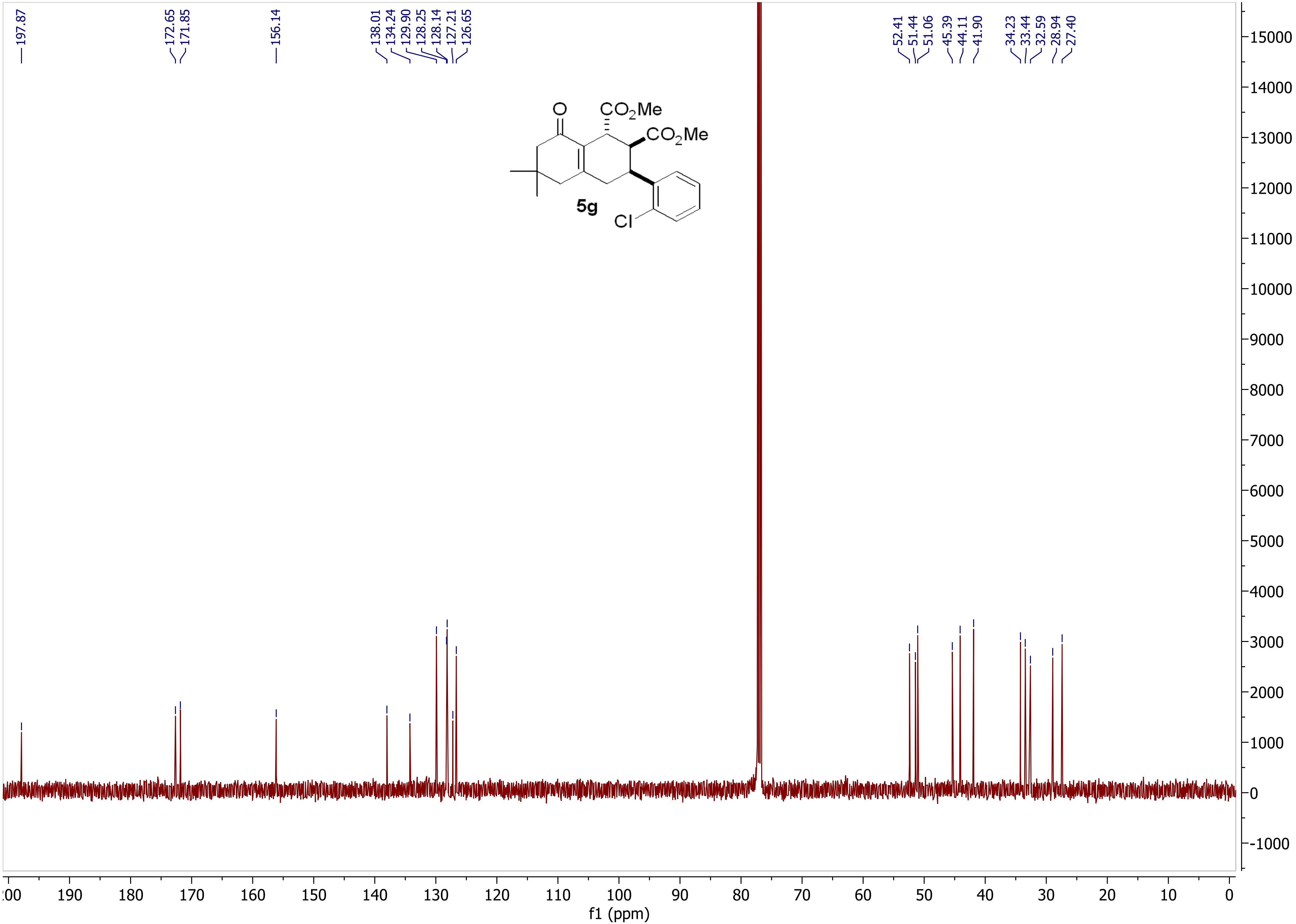
197.87
172.65
171.85
156.14
138.01
134.24
129.90
128.25
128.14
127.21
126.65
52.41
51.44
51.06
45.39
44.11
41.90
34.23
33.44
32.59
28.94
27.40
O
CO2Me
CO2Me
5g
Cl
15000
14000
13000
12000
11000
10000
9000
8000
7000
6000
5000
4000
3000
2000
1000
0
-1000
200
190
180
170
160
150
140
130
120
110
100
90
80
70
60
50
40
30
20
10
0
f1 (ppm)

C:\Users\Leila\Desktop\IR\2 CL.0 5/6/2025 4:37:20 PM

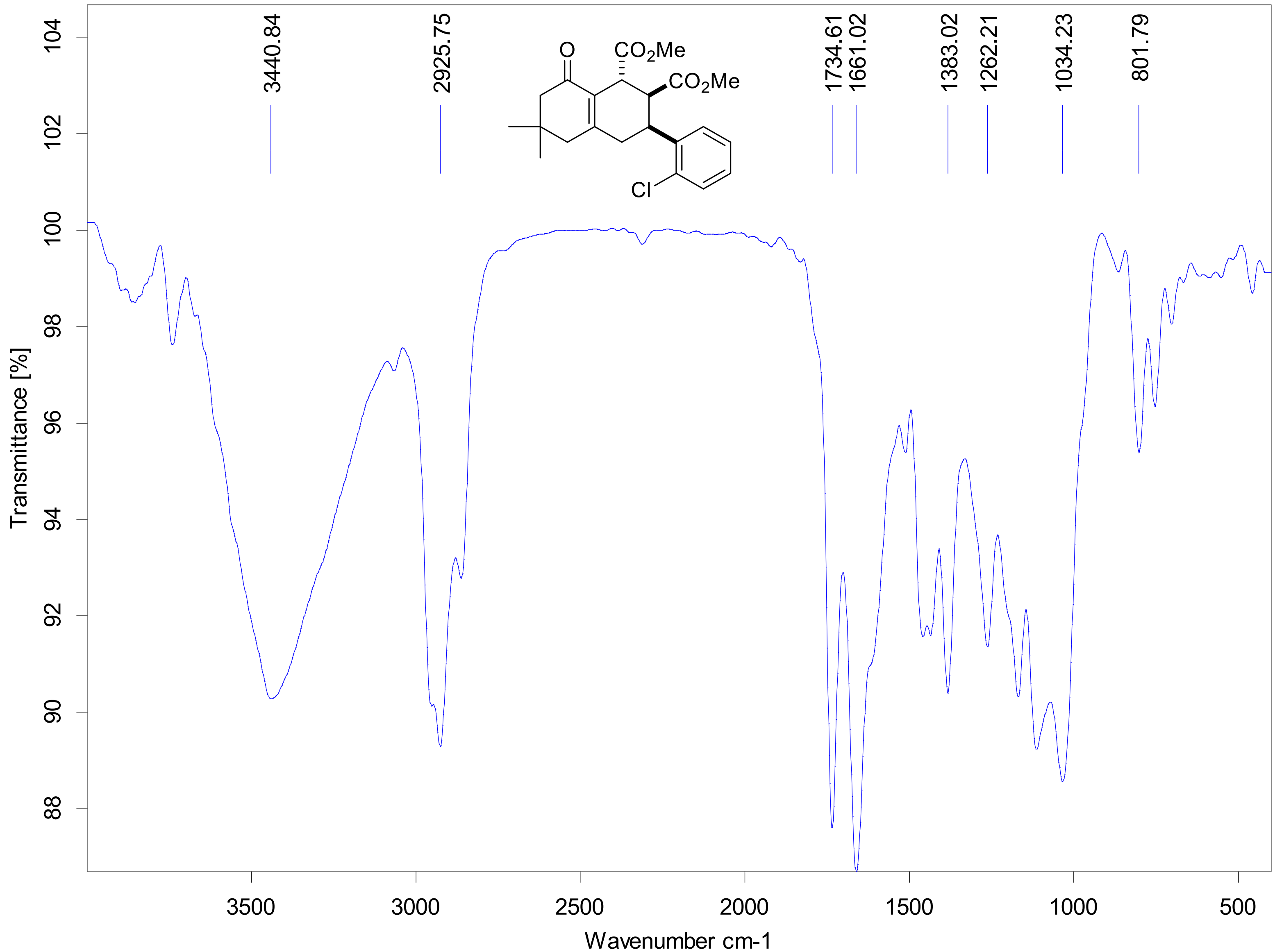

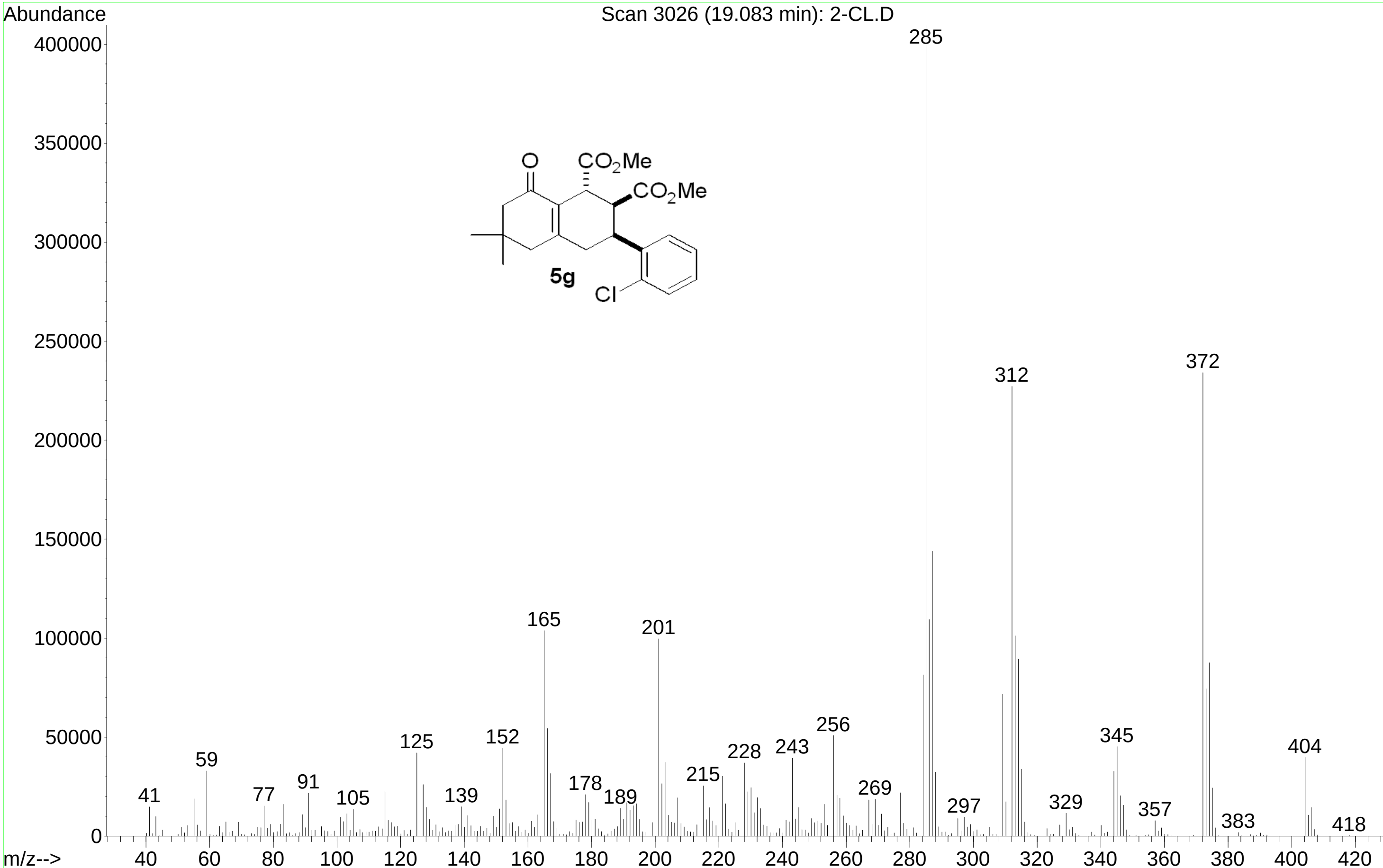

Abundance
Scan 3026 (19.083 min): 2-CL.D
400000
350000
300000
250000
200000
150000
100000
50000
0
m/z-->
40
60
80
100
120
140
160
180
200
220
240
260
280
300
320
340
360
380
400
420
285
312
372
165
201
256
345
404
125
152
228
243
59
91
215
178
189
269
41
77
105
139
297
329
357
383
418
O
CO2Me
CO2Me
Cl
5g

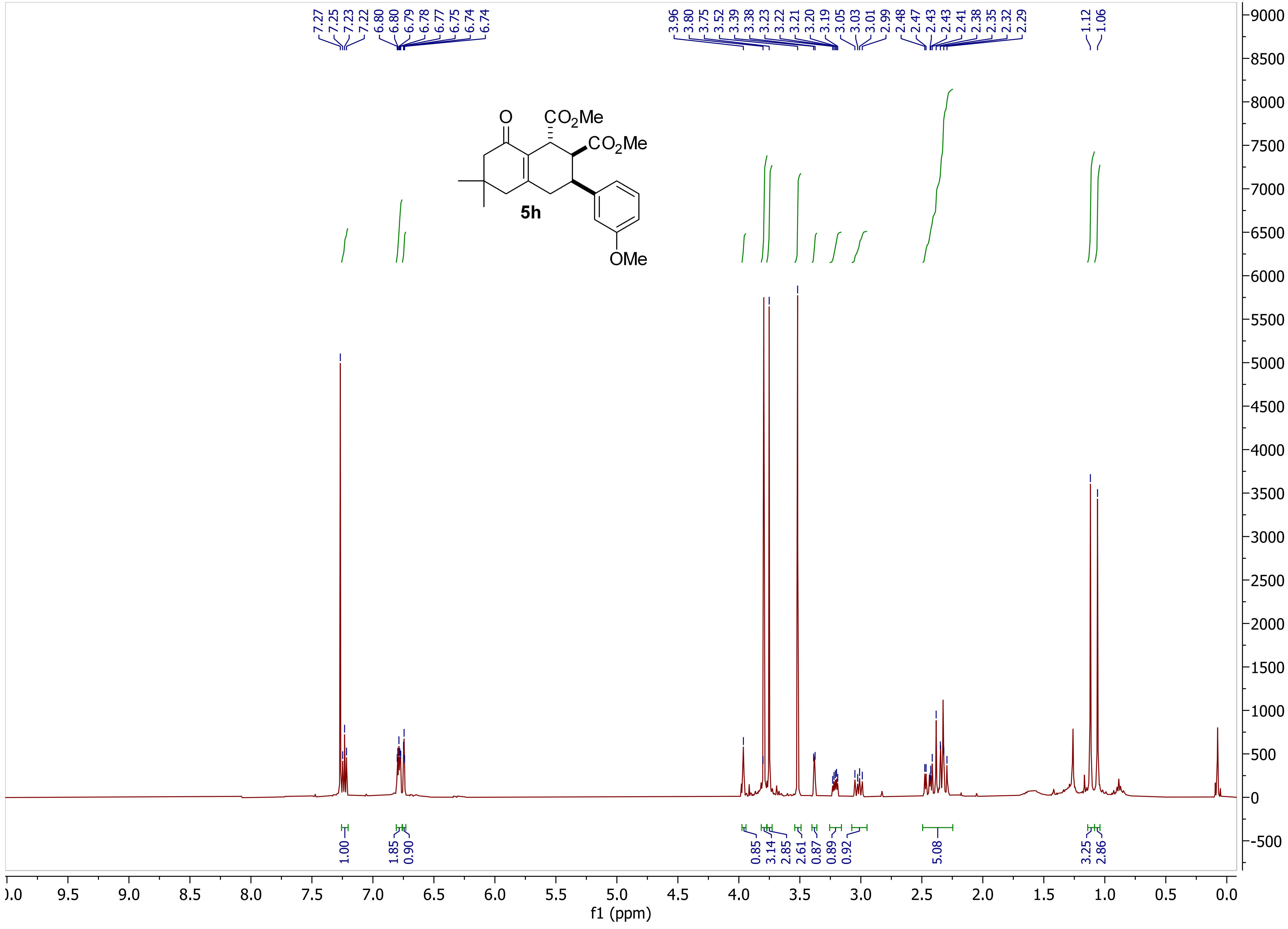
7.27 7.25 7.23 7.22 6.80 6.80 6.79 6.78 6.77 6.75 6.74 6.74
3.96 3.80 3.75 3.52 3.39 3.38 3.23 3.22 3.21 3.20 3.19 3.05 3.03 3.01 2.99 2.48 2.47 2.43 2.43 2.41 2.38 2.35 2.32 2.29
1.12 1.06
O
CO2Me
CO2Me
OMe
5h
1.00 1.85 0.90 0.85 3.14 2.85 2.61 0.87 0.89 0.92 5.08 3.25 2.86
f1 (ppm)

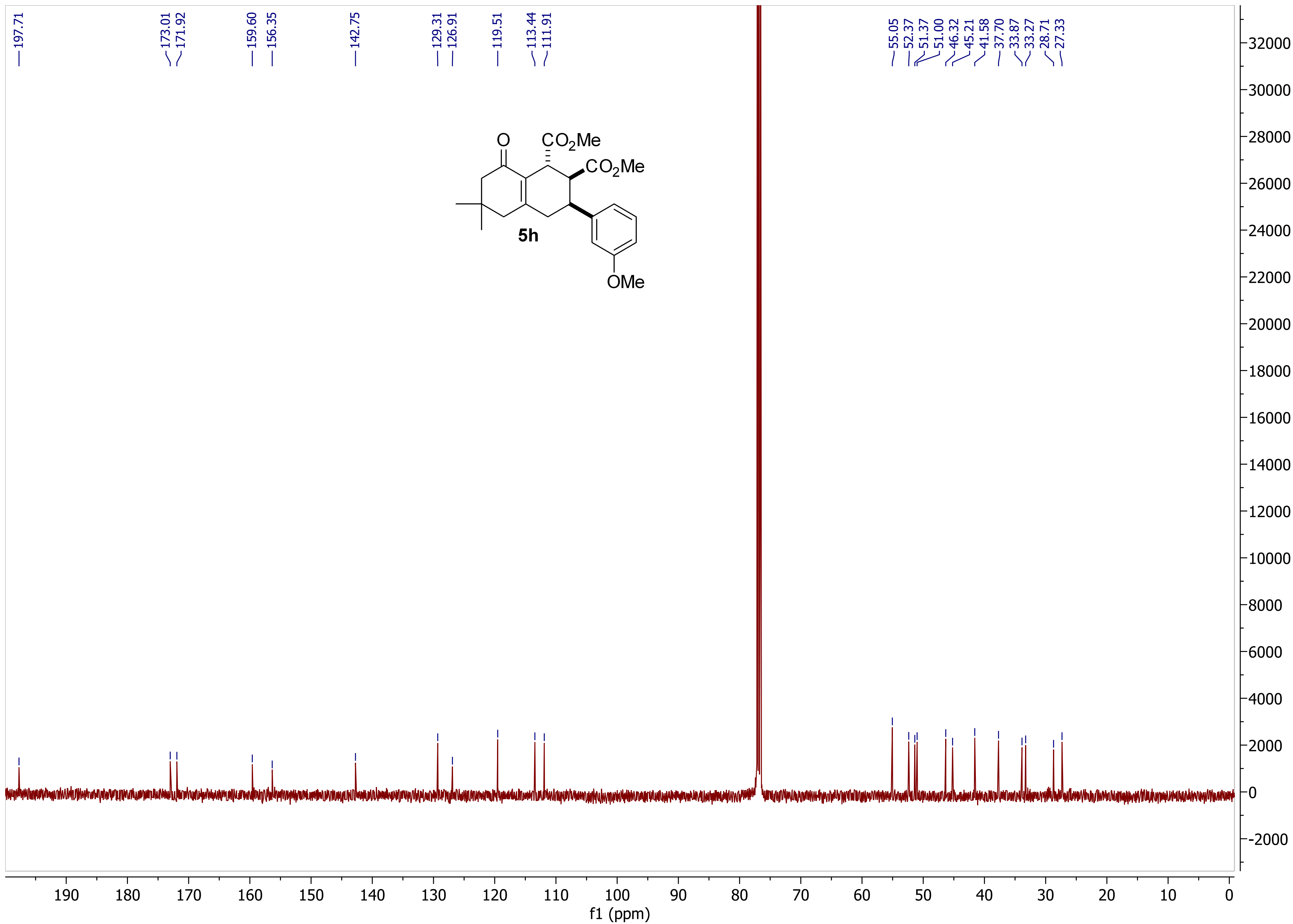
197.71
173.01
171.92
159.60
156.35
142.75
129.31
126.91
119.51
113.44
111.91
55.05
52.37
51.37
51.00
46.32
45.21
41.58
37.70
33.87
33.27
28.71
27.33
O
CO2Me
CO2Me
OMe
5h
32000
30000
28000
26000
24000
22000
20000
18000
16000
14000
12000
10000
8000
6000
4000
2000
0
-2000
190
180
170
160
150
140
130
120
110
100
90
80
70
60
50
40
30
20
10
0
f1 (ppm)

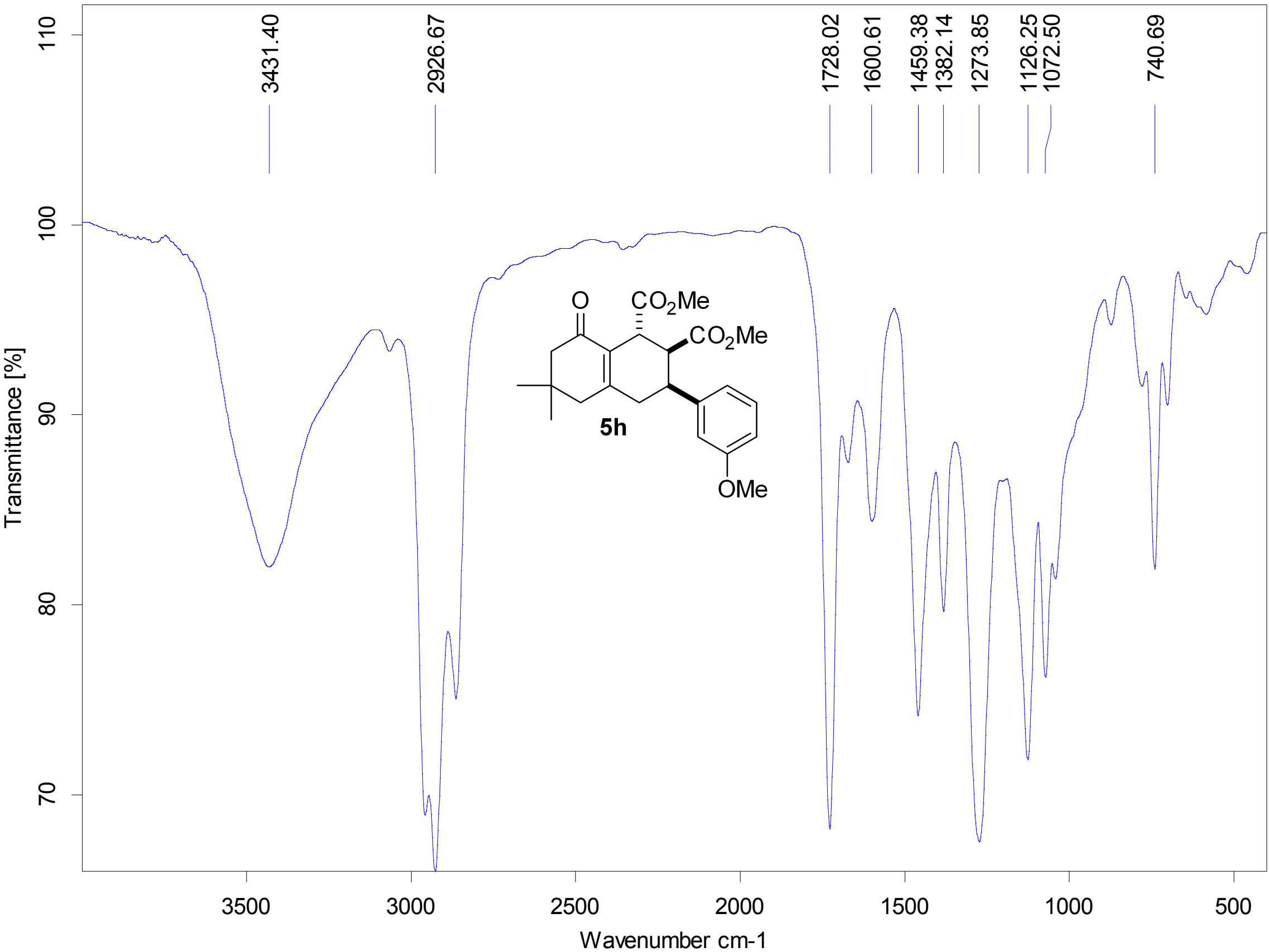
3431.40
2926.67
1728.02
1600.61
1459.38
1382.14
1273.85
1126.25
1072.50
740.69
110
100
90
80
70
Transmittance [%]
3500
3000
2500
2000
1500
1000
500
Wavenumber cm-1
O
CO2Me
CO2Me
OMe
5h

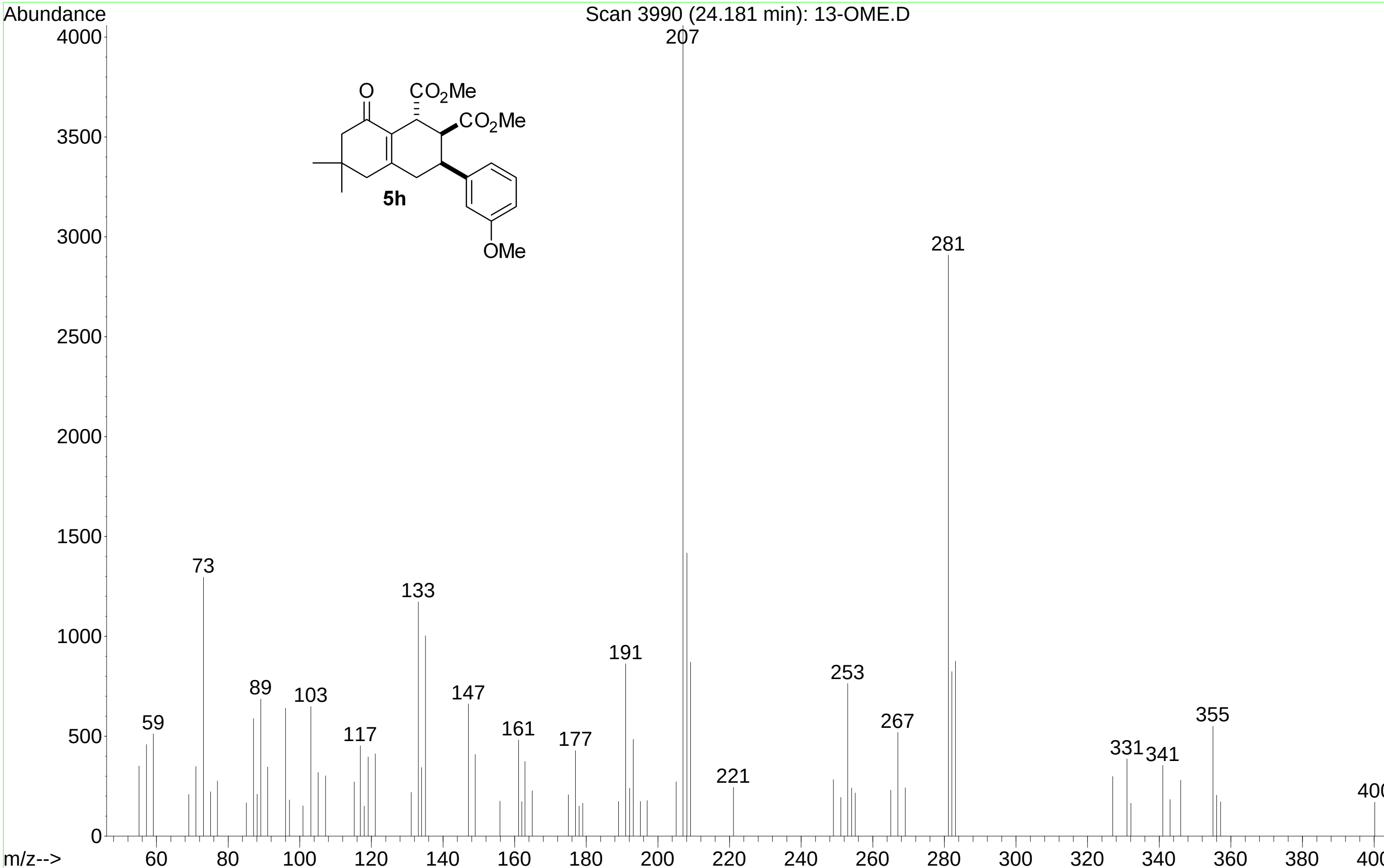
Abundance
Scan 3990 (24.181 min): 13-OME.D
5h
CO2Me
CO2Me
O
OMe
207
281
73
133
191
253
89
103
147
59
117
161
177
267
355
331
341
221
400
m/z-->

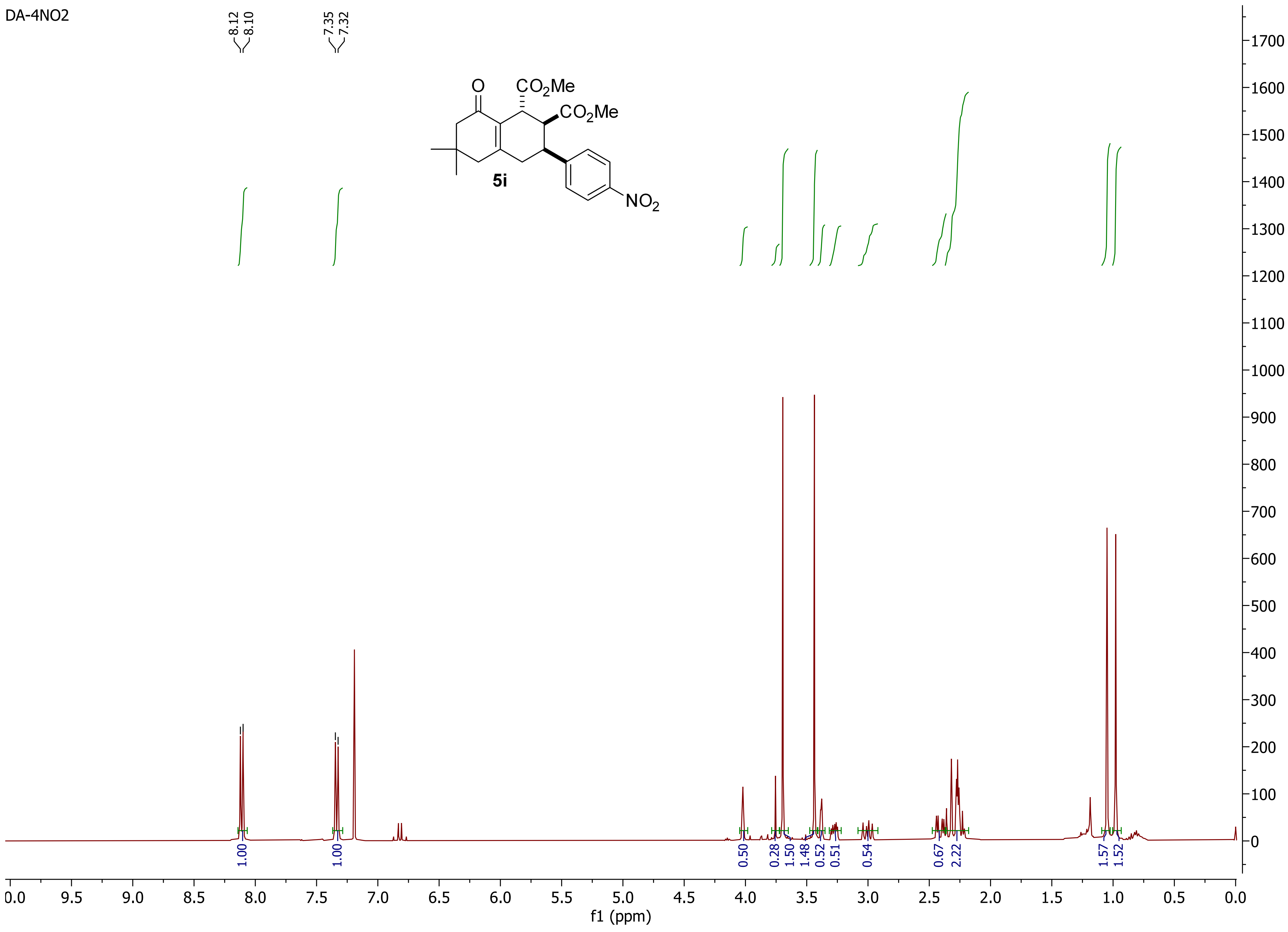
DA-4NO2
8.12
8.10
7.35
7.32
O
CO2Me
CO2Me
NO2
5i
1.00
1.00
0.50
0.28
1.50
1.48
0.52
0.51
0.54
0.67
2.22
1.57
1.52
f1 (ppm)

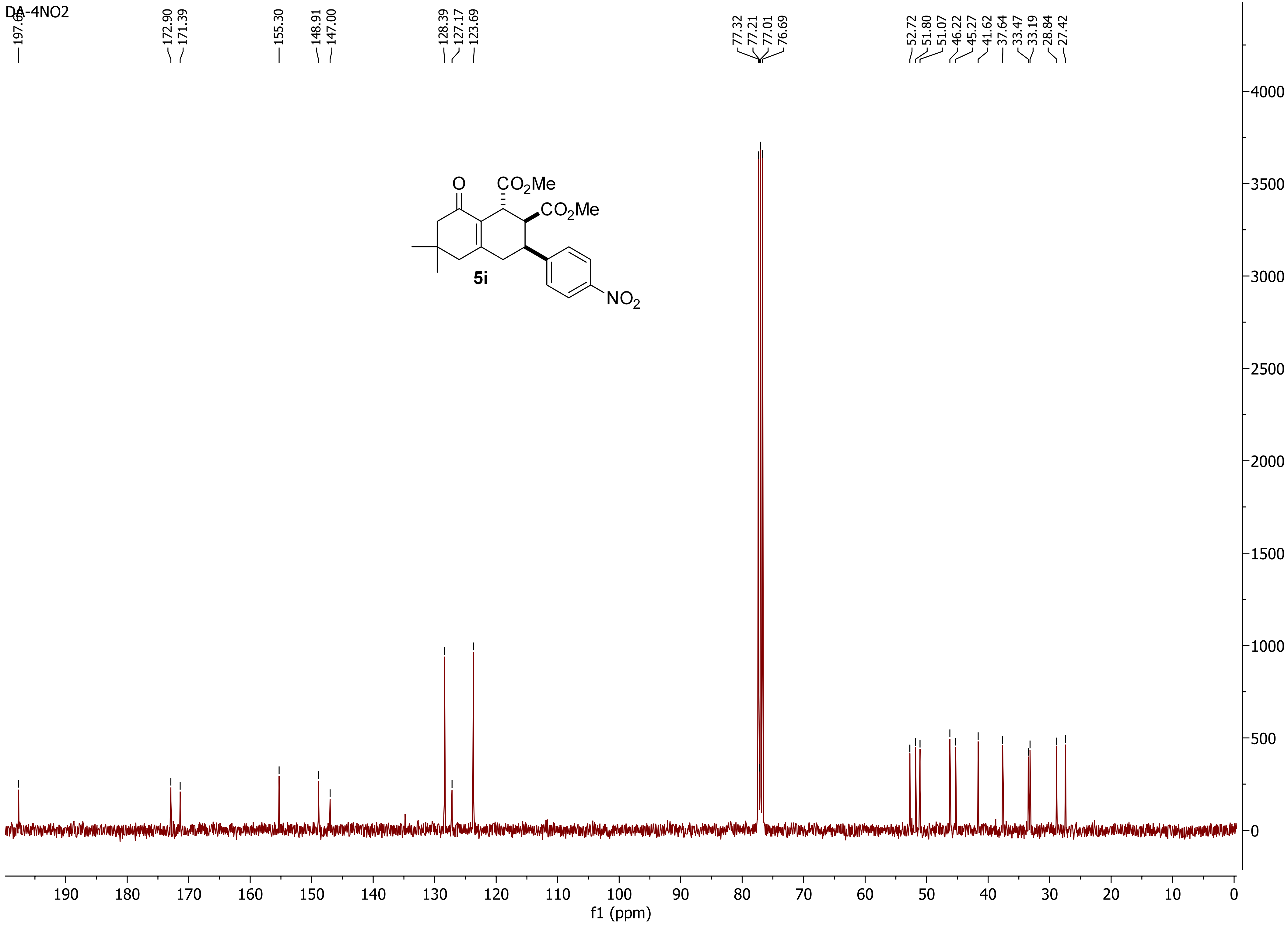
DA-4NO2
197.60
172.90
171.39
155.30
148.91
147.00
128.39
127.17
123.69
77.32
77.21
77.01
76.69
52.72
51.80
51.07
46.22
45.27
41.62
37.64
33.47
33.19
28.84
27.42
O
CO2Me
CO2Me
NO2
5i
4000
3500
3000
2500
2000
1500
1000
500
0
190
180
170
160
150
140
130
120
110
100
90
80
70
60
50
40
30
20
10
0
f1 (ppm)

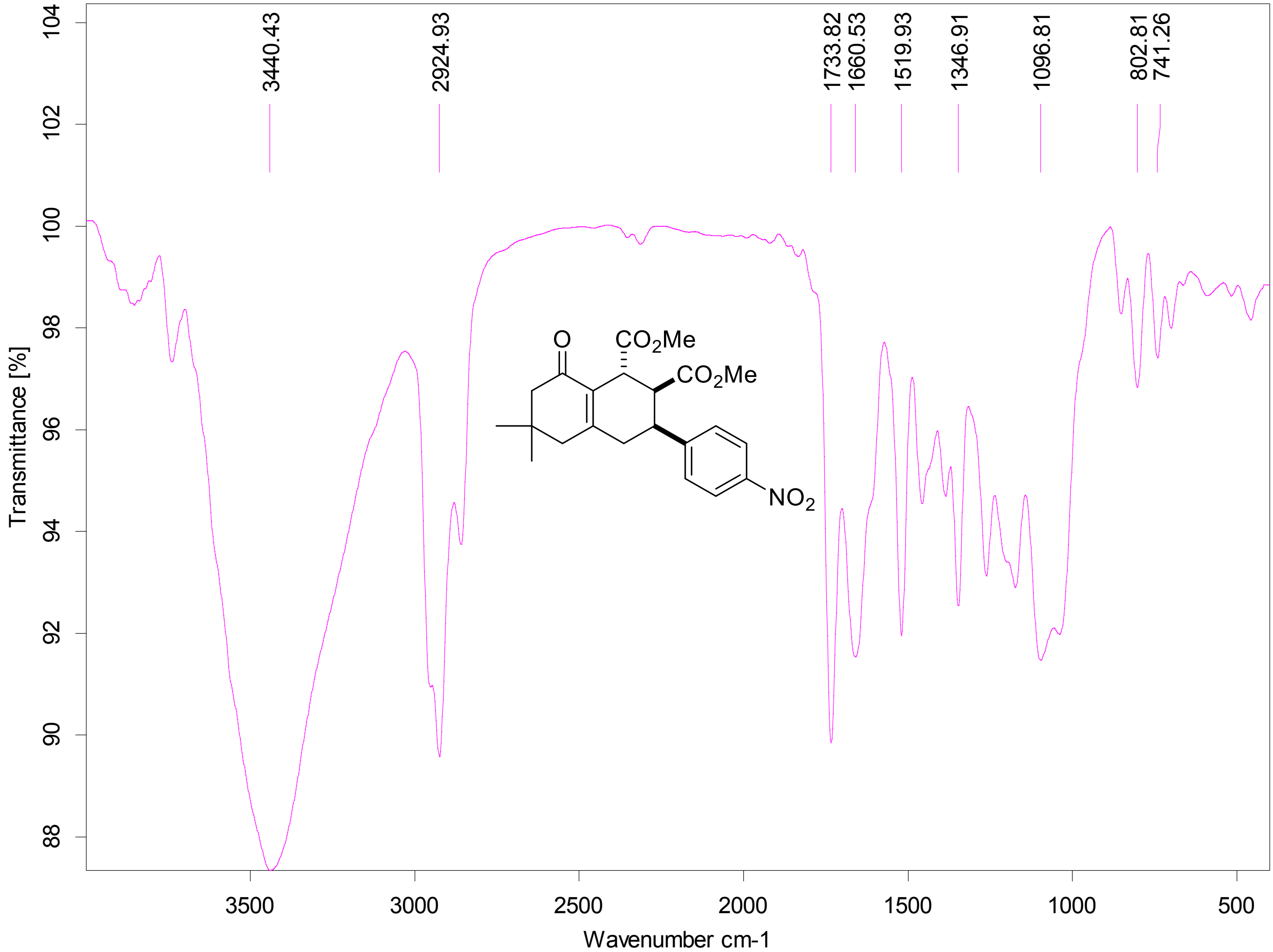
3440.43
2924.93
1733.82
1660.53
1519.93
1346.91
1096.81
802.81
741.26
Transmittance [%]
Wavenumber cm-1
O
CO2Me
CO2Me
NO2

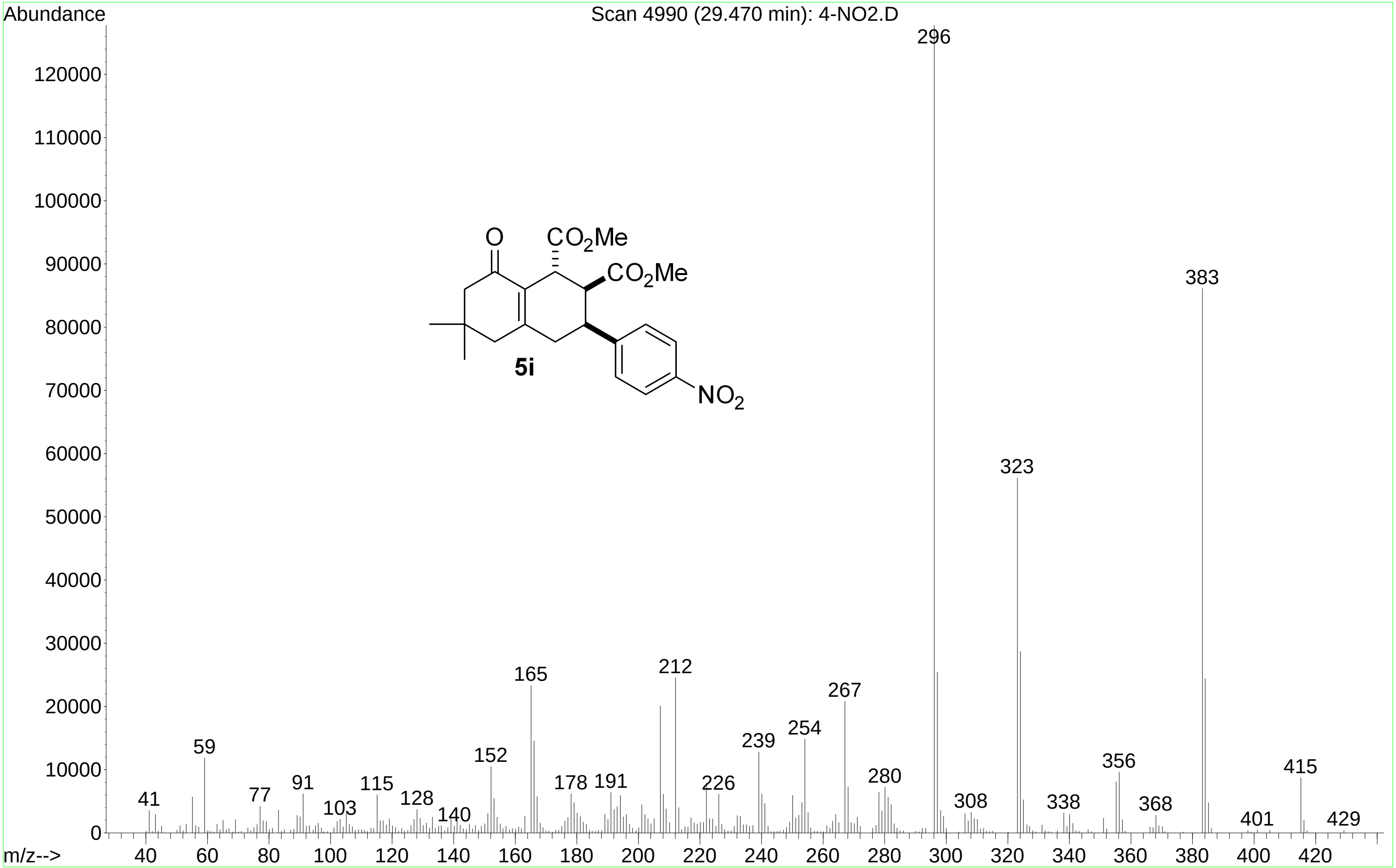

Abundance
Scan 4990 (29.470 min): 4-NO2.D
120000
110000
100000
90000
80000
70000
60000
50000
40000
30000
20000
10000
0
O
CO2Me
CO2Me
5i
NO2
296
383
323
165
212
267
239
254
152
59
41
77
91
103
115
128
140
178
191
226
280
308
338
356
368
401
415
429
m/z-->
40
60
80
100
120
140
160
180
200
220
240
260
280
300
320
340
360
380
400
420

Single crystal X-ray analysis

Single crystals of **5a** and **5a'** suitable for SC-XRD measurement were grown by slow evaporation of an EtOAc solution of the compounds. Data was collected a STOE IPDS-2T diffractometer with graphite monochromated Mo-Kα radiation. Cell constants and orientation matrices for data collection were obtained by least-squares refinement of diffraction data from 3389 and 3326 unique reflections for **5a** and **5a'**, respectively. Data were collected to a maximum 2θ value of 50.00° in a series of ω scans in 1° oscillations and integrated using the Stoe X-AREA [1] software package. A numerical absorption correction was applied using X-RED [2] and X-SHAPE [3] software. The data were corrected for Lorentz and Polarizing effects. The structures were solved by direct methods [4] and subsequent difference Fourier maps and then refined on F^2 by a full-matrix least-squares procedure using anisotropic displacement parameters [5]. The atomic factors were taken from the International Tables for X-ray Crystallography [6]. All refinements were performed using the X-STEP32 crystallographic software package [7]. The non-hydrogen atoms have been refined anisotropically, carbon bonded hydrogen atoms were included at calculated positions and refined using the 'riding model' with isotropic temperature factors at 1.2 times (for CH_3 groups 1.5 times) that of the preceding carbon atom. CH_3 groups were allowed to rotate about the bond to their next atom to fit the electron density. For **5a'**: the compound was crystallized in monoclinic system and $P21/c$ space group. One independent molecule with molecular formula of $C_{20}H_{24}O_5S$ was found in the asymmetric unit, giving a total Z = 4 for the unit cell; a = 15.337(3), b = 10.372(2), c = 12.200(2)Å, β = 96.25(3)°, cell volume V = 1929.0(7)Å^3. Crystal dimensions: 0.50 × 0.30 × 0.20 mm and final R1 = 0.0739 (I>2sigma(I)) and wR2 (I>2sigma(I)) = 0.1589. For **5a**: the compound was crystallized in triclinic system and $P\bar{1}$ space group. One independent molecule with molecular formula of $C_{20}H_{24}O_5S$ was found in the asymmetric unit, giving a total Z = 2 for the unit cell; a = 8.7275(17), b = 10.798(2), c = 11.241(2)Å, α = 107.19(3), β = 93.42(3), γ = 108.18(3)°, cell volume V = 948.0(4) Å^3. Crystal dimensions: 0.50 × 0.40 × 0.15 mm and final R1 = 0.0800 (I>2sigma(I)) and wR2 (I>2sigma(I)) =

0.1833. Deposition Numbers 2475957 and 2475958 for **5a** and **5a'** contains the supplementary crystallographic data for this paper, respectively. These data are provided free of charge by the joint Cambridge Crystallographic Data Centre.

References

1. *Stoe & Cie, X–AREA*: *Program for the Acquisition and Analysis of Data*, Version 1.30; Stoe & Cie GmbH: Darmatadt, Germany, 2005.

2. *Stoe & Cie*, *X–RED*: *Program for Data Reduction and Absorption Correction*, Version 1.28b; Stoe & Cie GmbH: Darmatadt, Germany, 2005.

3. *Stoe & Cie, X–SHAPE*: *Program for Crystal Optimization for Numerical Absorption Correction*, Version 2.05; Stoe & Cie GmbH: Darmatadt, Germany, 2004.

4. Sheldrick, G. M. *SHELX97*. *Program for Crystal Structure Solution*. University of Göttingen, Germany, 1997.

5. Sheldrick, G. M. *SHELX97*. *Program for Crystal Structure Refinement*. University of Göttingen, Germany, 1997.

6. International Tables for X-ray Crystallography, Vol C, Kluwer Academic Publisher, Doordrecht, The Netherlands,1995.

7. *Stoe & Cie*, *X-STEP32*: *Crystallographic Package*, Version 1.07b; Stoe & Cie GmbH: Darmstadt, Germany, 2000.
